# A synapse perspective on the function of the amyloid precursor protein

**DOI:** 10.1177/00368504251360728

**Published:** 2025-07-30

**Authors:** Pia Kruse, Amelie Eichler, Larissa Klukas, Maximilian Lenz

**Affiliations:** 1 Institute of Neuroanatomy and Cell Biology, 9177 Hannover Medical School, Hannover, Germany

**Keywords:** Amyloid precursor protein, Abeta, synaptic plasticity, synaptic transmission, Alzheimer's disease

## Abstract

The amyloid precursor protein (APP) is a transmembrane protein widely expressed throughout the brain, where it plays critical roles in both physiological and pathological states. APP undergoes complex post-translational processing by various secretases, a process that can lead to amyloid plaque formation via its amyloidogenic pathway. Consequently, APP has been extensively studied in the context of Alzheimer's disease (AD). However, emerging evidence highlights its physiological functions and the diverse roles of its cleavage fragments. This review explores the dual role of APP and its fragments, focusing on their contributions to synaptic structure, function, and plasticity. We summarize the mechanisms by which APP and its fragments influence synaptic dynamics and plasticity in the hippocampal CA1 region. These insights underline the importance of APP beyond amyloidogenesis, emphasizing its role in fundamental neurobiological processes and potential implications for understanding early AD-related synaptic dysfunction.

## Introduction

The amyloid precursor protein (APP) has been extensively studied in the context of Alzheimer's disease (AD) due to its role in generating amyloid plaques, which represents a hallmark of AD pathology. However, beyond its pathological implications, recent work has highlighted the physiological role of APP in the central nervous system. Given its dual involvement in physiology and pathology, understanding APP-mediated control of neural circuits may provide broader insights into basic features of synapses and early events that precede neurodegeneration.

Three independent research groups first described the APP in 1987.^1–3^ More than a century after Rudolf Virchow coined the term “amyloid,” and 80 years after Alois Alzheimer linked these deposits to neurodegeneration, researchers identified the precursor of these deposits as a cell-surface receptor. APP is ubiquitously expressed throughout the central nervous system, with high levels in both excitatory and inhibitory neurons, but also glial cells like astrocytes, oligodendrocytes, and microglia.^4–7^ It is a transmembrane protein that can be found particularly in regions associated with learning and memory, such as the hippocampus, cerebral cortex, and cerebellum.^8^ This widespread expression pattern suggests important roles for APP in neural network physiology, including synaptic integrity and plasticity.

APP possesses multiple protein domains with distinct functions, such as the Kunitz protease inhibitor (KPI) domain, which are encoded by individual exons. Both humans and mice express multiple APP isoforms generated by alternative splicing, which differ in their protein domains and expression patterns. The APP695 isoform, which lacks exons 7 and 8, does not contain the KPI domain (exon 7) or the OX2 immunoglobulin-like domain (exon 8). It is the predominant isoform in neurons and was previously linked to synaptic function.^9^ Both APP751 and APP770 contain the KPI domain within their extracellular region but differ in the presence of the OX2 domain. APP751 is expressed in various cell types, specifically in non-neuronal tissues and glial cells. KPI-containing APP was linked to plaque formation, which suggests the hypothesis that glial cells significantly contribute to the pathogenesis of AD.^10^ APP770 is predominantly expressed in peripheral tissues but shows increased expression in the brain upon inflammation and stress.^9^

APP belongs to a conserved family of proteins that includes APP-like proteins 1 and 2 (APLP1 and APLP2), sharing structural similarities that suggest overlapping and distinct functions in the nervous system. In neurons, which are morphologically complex cells, APP can be localized at synapses, both pre- and postsynaptically, with immediate implications for APP transport along neurites or local protein synthesis near synaptic sites.^11–14^ Once integrated into the plasma membrane, APP exhibits functional versatility through interactions in both *cis-* and *trans-*configurations. In the *cis-*configuration, APP molecules within the same cellular membrane can form homodimers or associate with other membrane proteins, influencing intracellular signaling pathways and the generation of cleavage products through membrane proteases.^12^ In the *trans-*configuration, APP on the surface of one cell interacts with APP or other ligands on adjacent cells.^15,16^ Such *trans*-cellular interactions are crucial for cell adhesion, synapse formation, and neural network integrity, facilitating communication and connectivity.^16–21^

Beyond its localization in the plasma membrane, APP can localize to mitochondria. In neurons and cell culture models, a fraction of full-length APP associates with the mitochondrial import machinery (TOMM40/TIM23), which can modulate the import of nuclear-encoded mitochondrial proteins and alter mitochondrial function.^22,23^ APP signaling can acutely regulate mitochondrial respiration and ATP production, potentially to meet activity-dependent energy demands.^24^ Moreover, APP and its fragments interact with axonal transport motors, of which kinesin-1 is an essential mediator of anterograde axonal transport.^25^ Thus, APP is capable of influencing the transport of crucial components for synaptic transmission, such as mitochondria,^26^ vesicles containing synaptic proteins,^27^ and secretase complexes.^28^ APP-mediated control of mitochondrial function and synaptic localization fuels synaptic transmission and contributes to synaptic calcium homeostasis, which is a well-established feature of mitochondria near synaptic sites.^29^

Physiologically, APP has been linked to the regulation of synaptic transmission through modulation of neurotransmitter release and synaptic vesicle recycling.^30–32^ Studies have demonstrated that APP can influence the probability of synaptic vesicle exocytosis, thereby affecting synaptic strength and efficacy.^12,33^ In recent work, we have demonstrated that APP restrains excitatory neurotransmission at medial perforant path synapses onto dentate granule cells.^34^ These results suggested distinct roles of pre- and postsynaptic APP, since only the presynaptic loss of APP was accompanied by a strengthening of excitatory neurotransmission. In line with a substantial body of evidence in the literature, this study supports the regulatory role of APP in synaptic transmission.

Beyond baseline synaptic transmission, a role for APP in stimulus-driven synaptic dynamics is well documented. APP is involved in both Hebbian synaptic plasticity, that is, long-term potentiation (LTP) and long-term depression (LTD), as well as homeostatic synaptic plasticity, since both knock-out and knock-in models targeting APP or its protein domains exhibit significant changes in plasticity.^35–37^ These findings suggest that APP is not merely a passive precursor for amyloid plaques but actively participates in the modulation of synaptic function and subsequent cognitive processes.

This review aims to provide a comprehensive overview of the current understanding of how APP and APP cleavage products regulate synaptic transmission and plasticity. First, we will summarize current knowledge on APP processing and molecular targets of APP and its fragments, which might recruit downstream signaling pathways. Subsequently, we provide a detailed overview of APP-mediated control of synaptic function in the CA1 region of the hippocampus, which is most frequently investigated in synaptic plasticity research. This review is based on a structured literature research (for overview and methodology, please see Supplemental Tables 1 to 3) and supplemented with additional references that provide further details in understanding the role of APP and its fragments. Moreover, this review is guided by the Scale for the Assessment of Narrative Review Articles (SANRA).^38^

## APP processing and molecular targets

**Figure 1.**
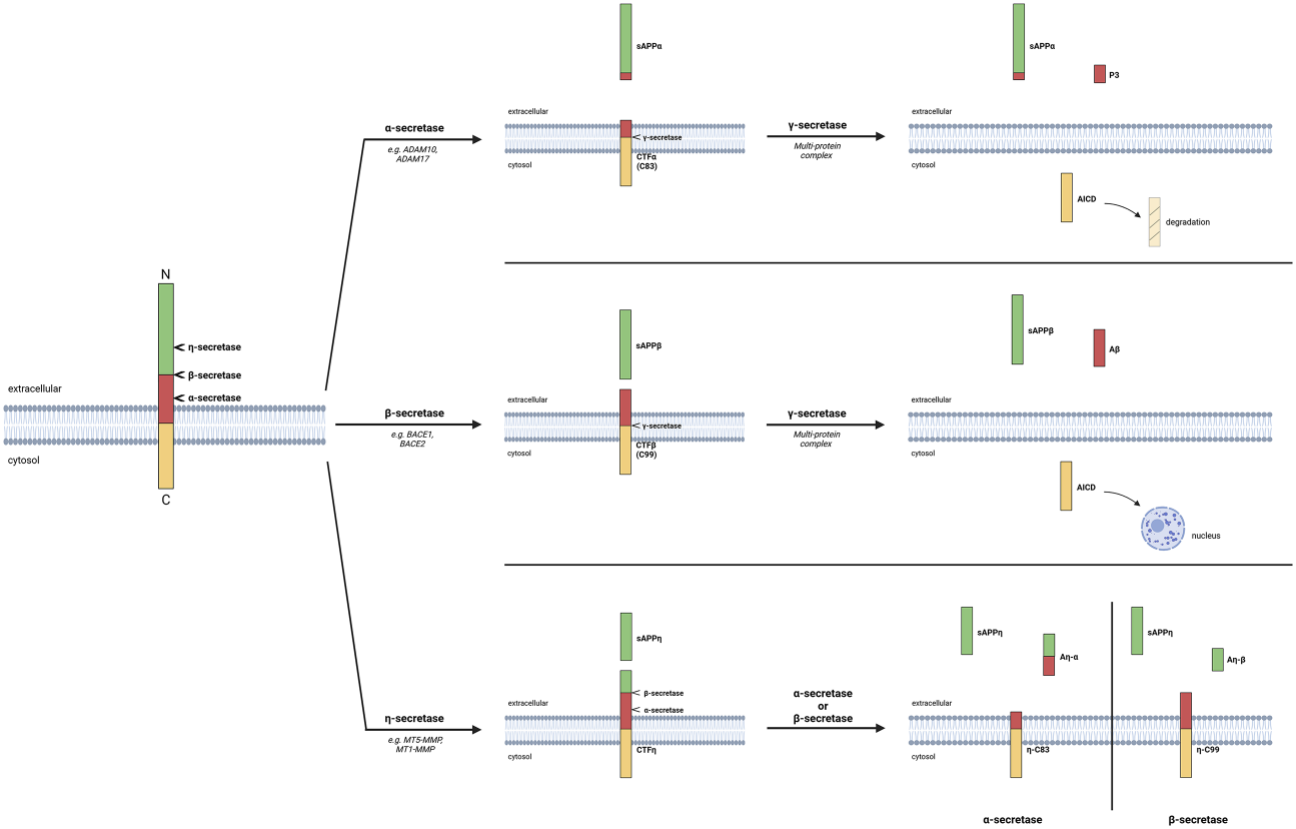
The non-amyloidogenic and amyloidogenic pathways in amyloid precursor protein (APP) processing. APP is a transmembrane protein with distinct intra-, trans-, and extracellular protein domains. It is a target of various secretases that determine the subsequent processing of APP. Initial APP cleavage with α-secretases leads to processing via the non-amyloidogenic pathway (upper panel). After α-secretase cleavage, the soluble APPα fragment is released. Then, the remaining transmembrane domain is cleaved by a γ-secretase, resulting in the extracellular release of the short P3 fragment and the intracellular release of the APP intracellular domain (AICD). Although degradation of this fragment is frequently reported, transcriptional regulatory effects might contribute to cellular effects related to APP processing. When full-length APP is initially cleaved by a β-secretase, it will further be processed via the amyloidogenic pathway (middle panel). Upon β-secretase cleavage, the soluble APPβ is released. The remaining transmembrane domain is cleaved by a γ-secretase, resulting in the extracellular release of Aβ and the intracellular release of the AICD, which can be translocated to the nucleus and influence gene transcription. Recently, a third pathway has been described, where APP is initially cleaved by a η-secretase (lower panel). Thereby, the soluble APPη is released extracellularly. Depending on a subsequent cleavage via α- or β-secretases, either Aη-α or Aη-β is released extracellularly. It is a matter of ongoing investigation whether the remaining transmembrane protein can further be processed by γ-secretases and might thereby play a role in extracellular Aβ release. Created in BioRender. Lenz, M. (2025) https://BioRender.com/e80s539.

APP is a transmembrane protein with different intra- and extracellular domains representing targets for membrane-bound proteases (Figure 1). The cleavage of APP is a highly regulated process that mainly occurs via two principal pathways: the non-amyloidogenic and the amyloidogenic pathway. Each of these is mediated by specific secretases, resulting in distinct cleavage products with specific functions.

In the non-amyloidogenic pathway, APP is initially cleaved by α-secretases, predominantly identified as members of the ADAM (A disintegrin and metalloproteinase) family, for example, ADAM10. In addition to ADAM10, which is considered the principal α-secretase, similar functions were reported for ADAM9, ADAM17, and ADAM19.^39,40^ This cleavage occurs within the Aβ region of APP, precluding the formation of amyloidogenic peptides. The result is the extracellular release of the large soluble ectodomain sAPPα and the generation of a membrane-bound C-terminal fragment (CTF), C83.^41^ Subsequent cleavage of C83 by the γ-secretase complex, which includes presenilin as the catalytic core, produces the APP intracellular domain (AICD) and a small non-amyloidogenic p3 peptide.^42,43^ AICD can be degraded or serve as a transcriptional regulator by translocating to the nucleus. However, nuclear signaling by AICD occurs predominantly upon amyloidogenic APP processing.^44^

Conversely, the amyloidogenic pathway is initiated by the action of β-secretases, primarily BACE1 (β-site APP cleaving enzyme 1^45^). BACE1 cleaves APP at the N-terminus of the Aβ domain, resulting in the secretion of sAPPβ and the membrane-bound fragment C99.^43,46^ Subsequent cleavage of C99 by the γ-secretase complex releases Aβ peptides in the extracellular space, predominantly Aβ_1–40_ and the more aggregation-prone Aβ_1–42_, along with the AICD in the cytoplasm.^43^ The AICD is capable of translocating to the nucleus, where it can influence gene expression.^47,48^ However, the relevance of endogenous AICD-mediated transcriptional regulation and target genes remains a controversial topic in the field.^49,50^

Recently, a novel processing of APP has been described, which relies on the cleavage of APP by η-secretases such as MT5-MMP,^51,52^ leading to a soluble sAPPη and a remaining transmembrane fragment CTF-η.^52,53^ CTF-η can be further processed through α- and β-secretases, resulting in two soluble cleavage products: Aη-β and Aη-α, and a remaining, shortened CTF-η domain. Apart from α-, β-, γ-, and η-secretases, recent studies discovered other processing pathways, including the meprin-pathway^54,55^ and the intracellular cleavage through the rhomboid-like protease RHBDL4.^51,56^

The cleavage products of APP, both extracellular and intracellular, exhibit fragment-specific functions and influence multiple signaling pathways with partially counteracting effects. Specifically, the balance of extracellular fragments originating from amyloidogenic or non-amyloidogenic APP processing determines critical functions of neuronal circuits, such as neuronal viability or signal integration. Notably, amyloidogenic cleavage products might mediate physiological functions as well, despite their involvement in plaque formation.^57,58^ Aβ_1–40_, for example, can bind to the E1 domain of APP and can induce intracellular G-protein-dependent pathways in presynaptic boutons.^12^ On the intracellular side, AICD translocating to the nucleus, which can influence gene expression on the genomic level by forming a transcriptionally active complex with Fe65 and Tip60,^59^ can be involved in feedback mechanisms. Although still a matter of debate, genes regulated by AICD target a variety of cellular functions that are crucial for network homeostasis, such as calcium signaling and cytoskeletal organization.^60^ Moreover, the essential role of CTFs in regulating cellular processes has recently gained further attention. Recent studies revealed that CTFs crucially regulate synaptic function, for example, through presynaptic mechanisms. CTFs can interact with synaptotagmin 7 (Syt7) in presynaptic terminals leading to impaired synaptic facilitation and replenishment of synaptic vesicles.^61^ This interaction at presynaptic terminals might be of particular interest in the context of presynaptic failure in AD models, where CTFs are overexpressed alongside other APP fragments.^62^

APP cleavage can be regulated through various mechanisms, including cellular lipids, with cholesterol playing a major role. Cholesterol-rich membrane microdomains, that is, lipid rafts, promote the β-secretase pathway by accumulating BACE1, and high membrane cholesterol enhances BACE1 activity and APP β-cleavage.^63^ Conversely, cholesterol depletion increases α-secretase cleavage of APP (raising sAPPα levels) while inhibiting β-cleavage, thereby lowering Aβ production.^64^ Moreover, lysolipids that accumulate under oxidative stress modulate APP processing. Lysolipids released by activated phospholipase A₂ in the brain have been shown to alter APP cleavage, as astrocyte-secreted group IIA sPLA₂ promotes α-secretase-mediated sAPPα release in neurons.^65^ With advancing age, cholesterol homeostasis in the brain becomes dysregulated (e.g. reduced efflux and oxidized cholesterol accumulation), and this is associated with a shift toward amyloidogenic APP processing.^66^ During aging and oxidative stress, elevated lysolipids and oxidized cholesterol derivatives can induce the clustering of APP and secretases in rafts, increasing Aβ generation in neuronal cultures.^67^

## APP-mediated regulation of synaptic transmission and plasticity in the hippocampal CA1 region

### APP-mediated regulation of synaptic structure

APP is widely recognized as a modifier of synaptic structure, which plays a role in functional aspects of neurotransmission. Upon release from the cell surface, soluble APP fragments can affect synapses over long distances, suggesting a significant impact on various cell types, which we here review with a focus on the hippocampal CA1 region. Notably, previous studies have reported that different cleavage products, fragment lengths of APP, and their polymerization state can have specific concentration-dependent effects.

**Figure 2.**
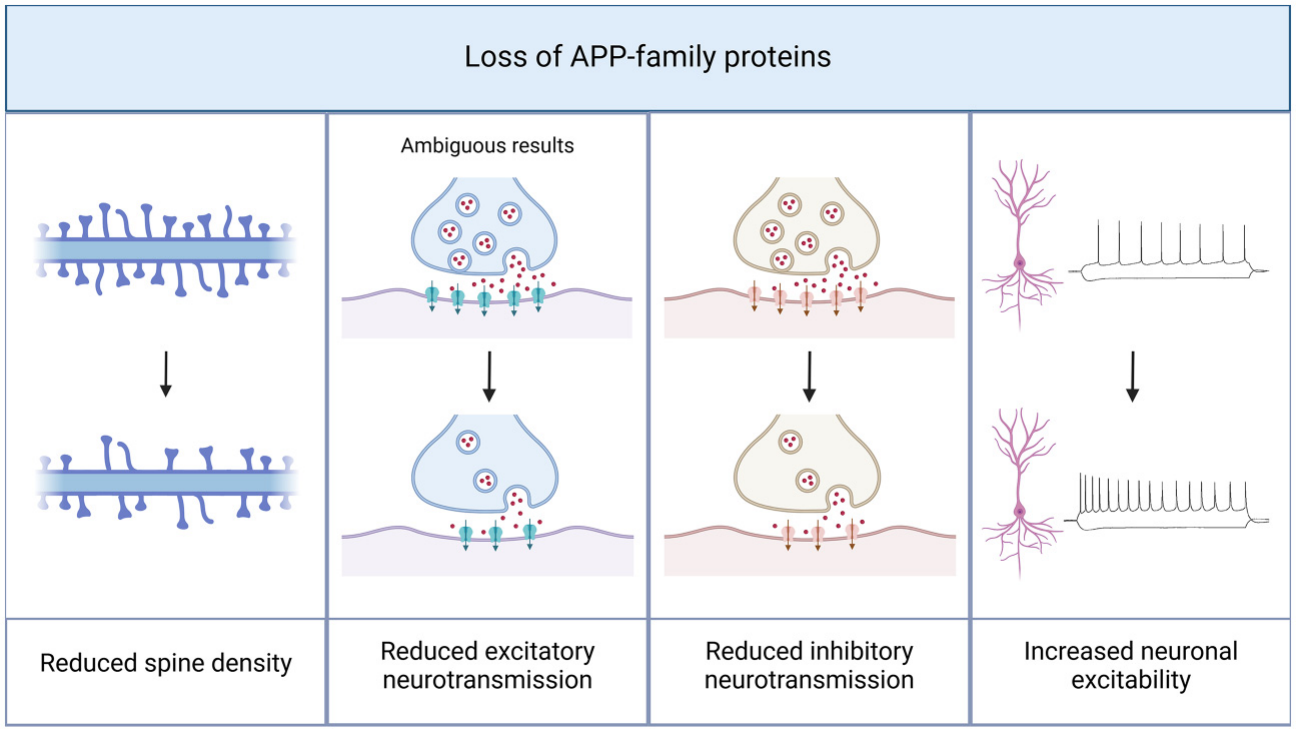
Amyloid precursor protein (APP) deficiency leads to structural and functional alterations in CA1 pyramidal neurons. The presence of APP is necessary to maintain functional neurotransmission. In APP knock-out models, structural changes like a decrease in spine density have been reported. In line with these observations, a crucial role of APP in synaptic transmission can be assumed. A knock-out of APP and other proteins of the APP family results in a reduction of both excitatory and inhibitory neurotransmission, with ambiguous results concerning excitatory neurotransmission. Furthermore, excitability of CA1 pyramidal cells is increased in APP knock-out models. Created in BioRender. Lenz, M. (2025) https://BioRender.com/q02l297.

The presence of APP and other members of the APP gene family is essential for maintaining the structural integrity of dendritic spines in CA1 pyramidal neurons. In APP/APLP2-deficient mice, reduced spine densities were found (Figure 2^37^). These alterations could be rescued by the exogenous substitution of sAPPα but not sAPPβ. This suggests a crucial role for orchestrated APP processing and sAPPα in the regulation of synaptic structure.

Contrary to the reported effects of sAPPα on spine structure, several studies suggest that APP fragments of the amyloidogenic pathway impair the structural integrity of synapses in CA1 pyramidal neurons by targeting both pre- and postsynaptic components, such as PSD95, GluR1, synapsin, and synaptophysin.^68–70^ In this context, the polymerization state of Aβ-fragments, that is, monomers, dimers, and oligomers, was identified as a crucial factor that regulates Aβ-function.^71–73^ While previous studies might not focus on this aspect specifically, future studies should address this variable in more detail.

**Figure 3.**
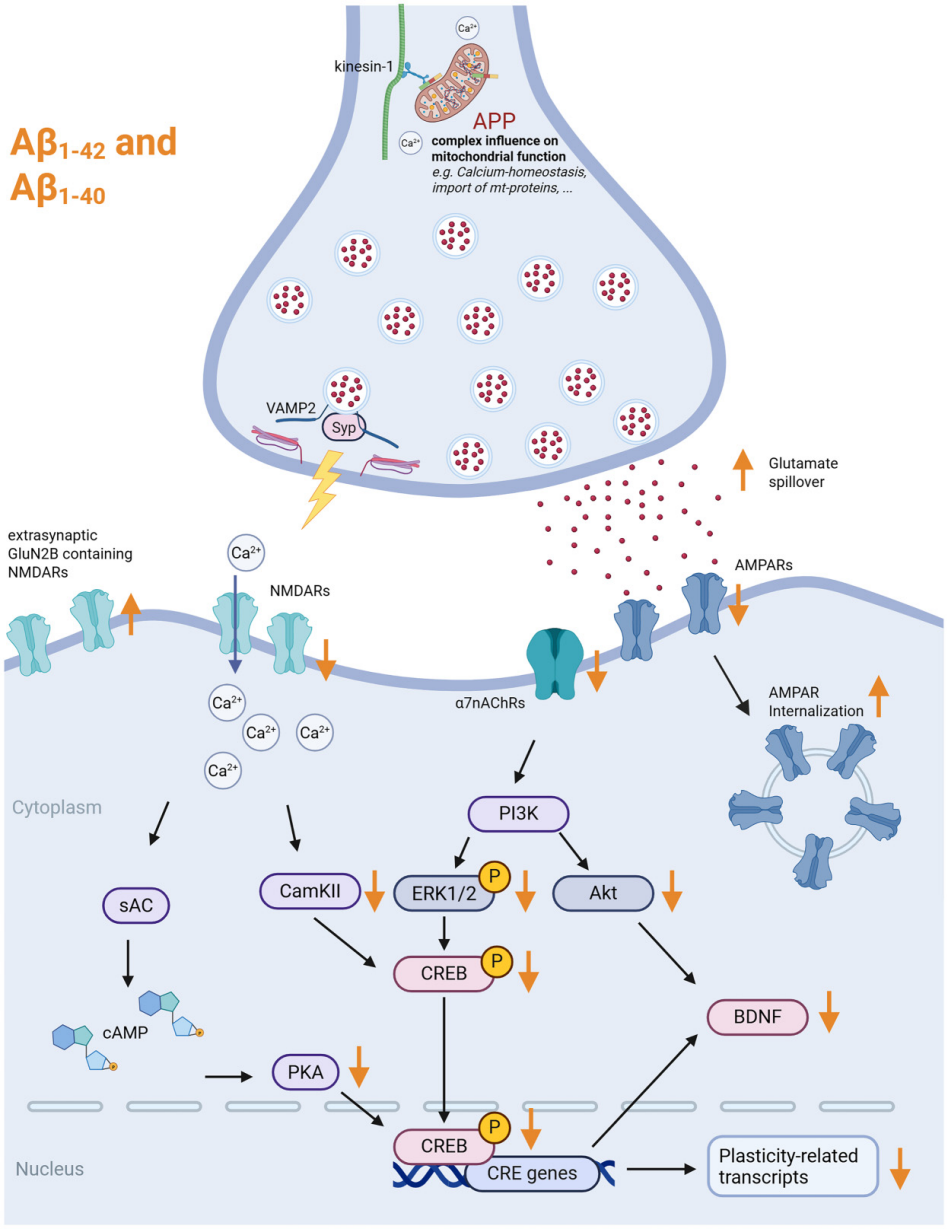
Effects of full-length APP, Aβ_1−40_, and Aβ_1−42_ on excitatory neurotransmission. Exogenous Aβ has various effects on excitatory synapses. On the presynaptic site, it can bind to Synaptophysin (Syp) and disrupt the Syp-VAMP2 complex. It leads to a downregulation of glutamatergic neurotransmission (AMPAR- and NMDAR-related), which leads to several downstream effects that result in the reduction of CREB activity and CREB-related transcripts. On the other hand, extrasynaptic receptors such as GluN2B-containing NMDARs can be upregulated and activated through glutamate spillover. This leads to an activation of excitotoxicity-related pathways (not shown). Furthermore, full-length APP can influence mitochondrial function, thereby, for example, impacting Ca^2+^-homeostasis at synaptic sites. Created in BioRender. Lenz, M. (2025) https://BioRender.com/a75j921. APP: amyloid precursor protein; VAMP2: vesicle-associated membrane protein 2; AMPA: α-amino-3-hydroxy-5-methyl-4-isoxazolepropionic acid; NMDA: N-methyl-D-aspartate; CREB: cAMP response element-binding protein; NMDARs: N-methyl-D-aspartate receptors.

For various APP fragments (Aβ_1–42_, Aβ_1–40_, and nitrated Aβ), a reduction in spine density has been described.^70,74,75^ In vivo injections of Aβ_1–42_ in adult mice led to a Ras-dependent loss of dendritic spines in CA1 pyramidal neurons, which was accompanied by a reduction of the postsynaptic marker PSD95 and the presynaptic marker synaptophysin.^74^ At the presynaptic site, Aβ_1–42_ can bind to synaptophysin and, after internalization in presynaptic boutons, disrupt synaptophysin/vesicle-associated membrane protein (VAMP) complexes, hampering synaptic integrity (Figure 3^76^). The reduction in dendritic spine density can coincide with decreased α7nAChR-densities, an effect also observed upon Aβ_1–40_ injection.^77^ These findings further support a strong link between amyloidogenic APP processing and cholinergic signaling. Given that α7nAChR signaling can recruit PI3K/Akt- and ERK-dependent BDNF release, Aβ-induced changes to BDNF levels might account for alterations in synaptic structure (Figure 3^74^). This aligns with the observed internalization of surface GluR1-containing AMPA receptors upon Aβ treatment,^78^ which might elicit functional changes (Figure 3). There are various targets of Aβ oligomers at postsynaptic sites, including essential components of both synaptic (neuroligin-1) and extrasynaptic (GluN2B-containing NMDA receptors) compartments (Figure 3^79^). Other studies, however, reported Aβ-driven enhancements of synaptic integrity. These included increased numbers of docked vesicles and activity in presynaptic boutons,^80,81^ increased lengths of PSDs, and altered plasticity resources in postsynaptic compartments.^82^ Conversely, experimental settings have been described, in which Aβ_1–42_ treatment was not associated with alterations in dendritic spine densities (200 nM, intracellular injections).^83^ Here, the polymerization state of APP processing products and their localization (intracellular vs. extracellular) might account for differential effects on synaptic structure. Comparing monomers, dimers, and oligomers of APP processing, previous reports show that oligomers but not monomers decrease dendritic spine densities after prolonged treatment periods.^71^ These changes required calcium-dependent signaling, including NMDAR, calcineurin, and cofilin. In line with structural changes, oligomers also reduced excitatory neurotransmission as discussed in the next section.^71^

While specific experimental conditions might account for the heterogeneity of Aβ-induced effects, we found that high concentrations of Aβ are mostly associated with synaptic disintegration, whereas low concentrations promote synapse integrity. Although the identity of Aβ-receptors remains widely unknown, we emphasize the dual role of Aβ in regulating synaptic integrity. We therefore suggest that changes in (optimal) Aβ-levels contribute to neural network homeostasis by promoting synaptic plasticity.

Apart from Aβ-driven regulations of synaptic structure, distinct functions for Aη have been described as well. Interestingly, Aη-fragments (100 nM) were able to alter the directionality of structural synaptic plasticity. While high-frequency glutamate uncaging typically results in dendritic spine growth, dendritic spine shrinkage was observed in the presence of Aη.^84^ Future studies will further elucidate Aη-induced switches between associative and non-associative structural plasticity, thereby improving our understanding of APP-mediated plasticity control.

### APP-mediated regulation of synaptic function

APP exerts its influence on synaptic function through a variety of mechanisms that involve extracellular and intracellular protein domains in both their cleaved and uncleaved forms. Previous studies have reported that APP can form homodimers at presynaptic boutons, thereby regulating presynaptic release probability through the N-terminal extracellular E1 (growth factor-like domain (GFLD^12^)). The intracellular JCasp domain has been shown to enable direct interactions with various presynaptic proteins.^85^ In this context, it has been demonstrated that JCasp-dependent signaling can limit excitatory neurotransmission and plasticity. These findings align with observations indicating that APP overexpression depresses excitatory neurotransmission, while inhibitory transmission remains unchanged.^30^ In contrast, APP-deficient neural tissue exhibits higher excitability (Figure 2^86^), accompanied by increases in excitatory neurotransmission involving changes in AMPA and NMDA receptor currents, and larger ready-to-releasable pools in presynaptic boutons.^31^ However, altered expression of other APP gene family members with similar functions might compensate for the monogenetic loss of APP.^85^ Therefore, distinct effects on excitatory neurotransmission, plasticity, and cellular excitability have only been demonstrated in triple-knockout (Triple-KO) tissues lacking APP, APLP1, and APLP2.^87^

Previous studies have identified the cleavage products of APP, such as Aβ, sAPPα, Aη, and CTFs, as crucial regulators of synaptic transmission. Depending on region- or compartment-specific secretase activity, the availability of APP cleavage products can be finely tuned, exerting immediate effects on neurotransmission at both glutamatergic and GABAergic synapses. Several studies have demonstrated that secretases themselves can be regulated by neural network activity, suggesting a physiological role for APP fragments in maintaining network homeostasis.^88^

At excitatory synapses, endogenous Aβ peptides of variable lengths have been shown to increase presynaptic transmitter release while leaving postsynaptic AMPA receptor density unaffected.^89^ However, viral overexpression of Aβ was found to be associated with excitatory synaptic depression attributable to a reduction in GluR2-containing AMPA receptors.^90^ Further studies have confirmed that Aβ fragments can depress excitatory neurotransmission, and have also explored a potential crosstalk between Aβ fragments and neuroligin-1.^79^ This leads us to the conclusion that the effect of soluble Aβ fragments might depend on multiple variables, including concentration, length of exposure, aggregation state, synapse type, brain region, and experimental model. As a general principle, shorter exposure times and lower concentrations seem to enhance synaptic transmission and plasticity, whereas higher concentrations and longer exposure times impair synaptic integrity.

Further studies have focused on the synaptic impact of distinct Aβ-isoforms. The Aβ_1–42_ fragment was initially linked to neurotoxicity and cell death. At presynaptic sites, Aβ_1–42_ affects synaptic vesicle recycling, with accumulation of recycled vesicles and an increase in the fraction of functional vesicles at individual terminals.^91^ Additionally, studies reporting increased fiber volley amplitudes, higher frequencies of miniature excitatory postsynaptic currents (mEPSCs), and enhanced presynaptic vesicle release support a robust presynaptic action of this fragment (Figure 3^92^). Notably, the increase in mEPSC frequency developed slowly (within 12 hours) and required p38MAPK-dependent actin polymerization.^81^

Moreover, postsynaptic effects of Aβ_1–42_ are well documented. Using in vivo injections, a Ras-dependent reduction in EPSP slopes could be detected in previous studies.^74^ Further ex vivo studies using higher Aβ_1–42_ concentrations (0.01–1 µM) have likewise demonstrated depressed excitatory neurotransmission, affecting both AMPA^93,94^ and NMDA receptors.^95,96^ These observations relied on calcium-dependent^97,98^ and microRNA signaling.^69^ They can result either from direct neuronal effects via intracellular actions of Aβ_1–42_^83,99^ or from cell-cell interactions, as demonstrated by Csf1-dependent synapse elimination by microglia.^93^ Intracellular oligomeric Aβ_1–42_ rapidly increases AMPA receptor-mediated EPSCs in a calcium- and PKA-dependent manner via the GluA1 subunit,^100^ but prolonged exposure results in decreased mEPSC amplitude and frequency.^75^ Notably, despite multiple reports of Aβ_1–42_-driven plasticity in synaptic compartments, encompassing both enhancement and depression, some studies provide evidence for plasticity impairment after Aβ_1–42_ exposure.^101–103^ Although these studies do not rule out metaplastic changes—that is, the ability of synapses to express plasticity—the inherent heterogeneity of Aβ_1–42_-induced synaptic effects should be taken into account in data interpretation.

Synaptic effects were also reported for the shorter Aβ_1–40_ fragment, which is less prone to aggregation than the Aβ_1–42_ fragment. Here, previous studies demonstrated that exposure to Aβ_1–40_ leads to a dose-dependent depression of EPSCs, affecting both pre- and postsynaptic mechanisms. The effects were reversible by phosphatase inhibitors targeting PP2B and partially by PP1/PP2A inhibitors.^104^ In vivo intraventricular infusion of Aβ_1–40_ was associated with decreased presynaptic activation. These changes correlated with a reduction in α7β2nAChR function, whereas no changes were observed for α4β2nAChRs.^105^ In contrast, other experimental conditions revealed enhancing effects on presynaptic function, leading to increased frequencies of excitatory postsynaptic events,^12,106^ potentially involving alterations in the presynaptic vesicle cycle and glutamate reuptake. Regardless of these considerations and due to a substantial heterogeneity in current literature, Aβ_1–40_-driven effects on postsynaptic glutamatergic function remain poorly understood.

The non-amyloidogenic APP processing pathway also contributes to the regulation of synaptic features. sAPPα can bind to metabotropic GABA_B_R1α at presynaptic sites,^107^ which was also demonstrated for distinct protein domains of full-length APP.^108^ While some results indicate that this interaction might suppress presynaptic vesicle release probability and consequently enhance short-term plasticity,^107^ others could not confirm that sAPPα has an effect on receptor signaling and synaptic transmission.^108^ Thus, sAPPα-mediated effects on synaptic transmission remain controversial. Further studies revealed that sAPPα can rescue LTP in APP/APLP2-deficient mice.^37^ In this context, the α7nACh receptor represents a target for the plasticity-restoring effects of sAPPα.

The Aη fragment, produced by η-secretase action on APP, primarily influences excitatory neurotransmission by directly altering NMDA receptor function. Previous studies have shown that physiological Aη levels are required for normal NMDA receptor function.^84^ Increasing Aη levels (e.g. 100 nM pharmacological treatment) alter NMDA receptor conformation and reduce calcium influx following receptor activation.^84^ Similar changes have been observed related to increased levels of endogenous Aη.

In addition to synaptic effects caused by extracellular fragments, distinct functions have also been described for the intracellular cleavage product of APP, AICD, and CTFs.^61^ The AICD fragment, which is primarily acting upon amyloidogenic APP processing,^44^ can increase NMDAR transmission by promoting the transcription of the GluN2B NMDAR subunit.^109^ At the same time, AMPAR-mediated excitatory synaptic transmission remains unchanged. GluN2B-containing NMDARs are enriched at extrasynaptic sites, exhibit slow kinetics, and are associated with excitotoxicity, a phenomenon observed in AD.^110^ Given these findings, it is interesting to speculate that extensive APP processing in neurodegenerative diseases might lead to AICD-driven increases in cellular vulnerability and resulting cell death.

### The APP regulates synaptic plasticity

**Figure 4.**
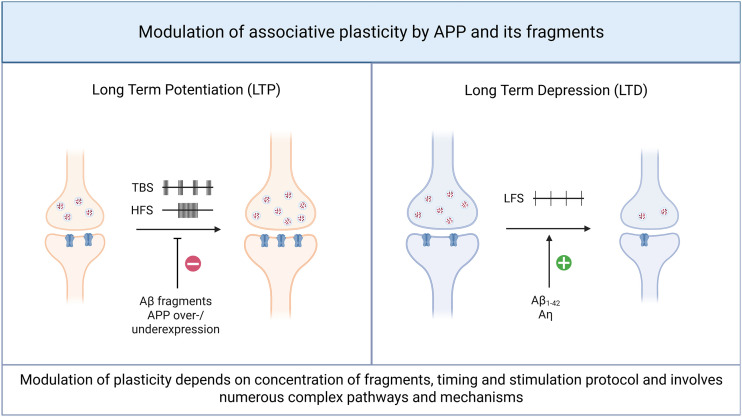
Amyloid precursor protein (APP) and its fragments modulate associative plasticity in the hippocampal CA1 region. Overall, various Aβ fragments, along with the dysregulated expression of full-length APP (either overexpression or underexpression), have been shown to impair LTP induced by TBS or HFS (left panel). Conversely, LTD induction by LFS appears to be facilitated by specific Aβ fragments and Aη. However, the precise mechanisms underlying these effects remain incompletely understood. The modulatory influence seems to depend on multiple factors, including the timing, concentration of the fragments, the specific stimulation protocols, and the experimental model employed. Created in BioRender. Lenz, M. (2025) https://BioRender.com/t25r054. APP: amyloid precursor protein; LTP: long-term potentiation; TBS: theta-burst stimulation; HFS: high-frequency stimulation; LTD: long-term depression; LFS: low-frequency stimulation

The expression of synaptic plasticity is a fundamental prerequisite for the physiological function of neural circuits.^111^ Over the past decades, numerous efforts have revealed that mechanisms of synaptic plasticity involve both positive (Hebbian) and negative (homeostatic) feedback loops. The expression of long-term potentiation, an associative form of synaptic plasticity often assessed by electrical pathway stimulation, is impaired in both the overexpression^94^ and genetic deletion of full-length APP (Figure 4). Impaired LTP in tissue that lacks essential genes of the APP gene family, such as triple-mutant APP/APLP1/APLP2 mice, can coincide with a decrease in spine density and altered basal excitatory synaptic transmission.^87^ Notably, LTP-diminishing effects of APP-deletion were primarily reported following burst stimulation, while plasticity induction with high-frequency stimulation remained unchanged.^35,86,112^ This observation coincides with increased paired-pulse facilitation in APP-deficient tissue,^86^ suggesting that presynaptic changes, such as changes in ready-to-releasable vesicle pools or calcium buffering capacities, might account for APP-related plasticity defects.

Besides changes in short-term transmitter release dynamics at presynaptic terminals,^74,80,105,113^ amyloidogenic APP processing also interferes with the ability of CA1 pyramidal cells to express long-lasting changes in excitatory synaptic strength, for example, LTP.^114^ It is commonly observed that Aβ-peptides inhibit the expression of LTP upon high-frequency stimulation (HFS),^78,101,114^ or theta-burst stimulation (TBS; Figure 4),^115,116^ which can be associated with alterations in baseline synaptic transmission.^117^

The inhibitory action of Aβ-peptides on LTP seems to involve various pathways, including their interaction with calcium-permeable surface receptors, such as GluA1 homotetramers^118^ and GluN2B-containing NMDA receptors.^70,119^ Accumulating evidence for Aβ-actions in extrasynaptic compartments, that is, the activation of GluN2B-containing NMDA receptors through glutamate spillover,^101^ further allows indirect correlations between inflammation,^120^ neurotoxicity, and impaired synaptic plasticity. Of note, these findings have been linked to compartment-specific vulnerabilities of neurons due to differential actions on apical and basal dendrites of pyramidal neurons.^120^ Further studies have revealed even more mechanistic insights in Aβ-induced impairment/modulation of LTP, such as alterations in ubiquitination,^121^ GIRK-mediated cellular excitability,^122^ endocannabinoid signaling,^123^ glucocorticoid receptors,^82^ Ras-^74^ and PKA/CREB-signaling pathways,^121^ mGluR5 signaling,^124^ AMPK-, GLP1-, and eEF2K-activity,^125^ as well as Csf1R-dependent microglial actions on synaptic transmission.^93^ Taken together, these observations unravel numerous pathways, highlighting the complexity and temporal sensitivity of Aβ-mediated effects on synaptic function.

In general, the importance of Aβ in the regulation of synaptic plasticity is demonstrated by plasticity defects in the absence of Aβ. Multiple studies, therefore, suggest a physiological function of Aβ peptides in defining plasticity thresholds. Aβ-related regulation of plasticity, encompassing both enhancement and impairment, depends on the respective fragment type, concentration, treatment time, as well as the polymerization state. Aβ_1–42_ peptides appear to have a higher efficiency in impairing LTP than shorter Aβ_1–40_ peptides (IC50 values for LTP-impairment: Aβ_1–42_: 2 nM, Aβ_1–40_: 9 nM^70^). While detrimental effects on plasticity predominate with prolonged treatment (>3 hours), shorter treatment periods seem to facilitate the expression of LTP (<1 hour at 200 pM^81^). TBS-induced LTP was consistently enhanced in the presence of Aβ_1–42_ oligomers at low concentrations (200 pM) while high concentrations (200 nM) exerted detrimental effects.^126^ However, the impairing effect on LTP expression has been reported across a broad concentration range (pM-µM^103,127^). Other studies have confirmed plasticity impairments upon Aβ_1–42_ oligomer exposure associated with subsequent intracellular accumulation.^99^ In contrast, when applied in monomeric form, Aβ_1–42_ at low concentrations (200 pM) exhibited only minor effects on TBS-induced LTP, with a moderate impairment at high concentrations.^92^ Since the influence of Aβ polymerization is not consistently analyzed throughout the literature, future studies should address this essential point in more detail.

Again, the α7nAChR appears to take a prominent role in orchestrating the concentration-dependent effects of Aβ_1–42_, since plasticity enhancement relies on the functionality of α7nAChR.^102^ In addition, 10 nM of Aβ_1–42_ (monomor/oligomer-mixture) impaired α7nAChR-driven LTP.^113^ However, cholinergic LTP through mAChR was not affected by Aβ_1–42_.^128^

For Aβ_1–40_, which is the shorter fragment of amyloidogenic APP processing, a consensus on LTP-inhibiting actions has emerged from current literature.^92,129^ Similar mechanisms for the longer Aβ_1–42_ fragment also apply to Aβ_1–40_, such as interference with cholinergic transmission through nAChRs.^105,130^ Aβ_1–40_ treatment caused a reduction in α7nAChR currents, which was linked to deficits at Schaffer collateral-CA1 synaptic transmission and plasticity, including LTP, post-tetanic potentiation (PTP), and paired-pulse facilitation (PPF^105^). Despite these commonalities, LTP impairment by either Aβ_1–40_ or Aβ_1–42_ showed distinct mechanistic differences, as demonstrated by their NMDAR dependence. While radiprodil (GluN2B-selective antagonist) and xenon were able to restore impaired LTP in CA1 in Aβ_1–42_ oligomer pre-incubated acute slices, no ameliorating effects were detected in Aβ_1–40_ pre-incubated slices.^119^^,^^131^.

Since both Aβ_1–40_ and Aβ_1–42_ modulate plasticity in CA1 neuronal networks, a substantial body of studies has investigated whether distinct parts of Aβ-peptides are crucial for mediating these effects. For Aβ_15–25_, no LTP impairment was detected.^132^ In contrast, Aβ_25–35_ exposure impaired LTP induction,^95,129,133^ which was time- and concentration-dependent.^132,134^ Aβ_25–35_-induced LTP impairments were rescued, for example, by JNK-signaling pathway inhibition,^135^ erythropoietin supplementation,^136^ inhibition of cholinergic transmission,^137^ and blockade of calcium channels.^138^ Interestingly, both sequence reversal (Aβ_35–25_) and shortening to Aβ_31–35_ impaired LTP expression as well.^130,132,134^ Therefore, it can be concluded that specific peptide regions within Aβ fragments are important for modulating synaptic plasticity.

Moreover, the C-terminal domain of APP and AICD-fragments have been shown to further modulate the ability of neuronal networks to express LTP.^109,139,140^ LTP-enhancing effects, which may facilitate the restoration of age- or degeneration-induced deficits, have also been described for sAPPα.^141–144^ Therefore, a substantial body of evidence emphasizes that APP processing is a potent modulator of LTP induction.

LTD, a sustained reduction in synaptic strength upon appropriate stimuli, can also be affected by Aβ-peptides. Aβ_1–42_ oligomers facilitate LTD in a frequency-dependent fashion (Figure 4^116,139^), with evidence of stronger facilitation in aged neural circuits.^78^ In vivo, Aβ_1–42_ enhanced a distinct form of LTD that is independent of muscarinic AChRs and NMDA receptors, relying instead on mGluR5 activation and the recruitment of prion protein (PrPc^124^). Moreover, Aβ_1–42_ oligomer exposure can induce tau hyperphosphorylation, which was linked to enhanced glutamate release probability and further enhancement of LTD.^145^ Similar LTD-enhancing effects have been reported for Aη (Figure 4^84^) and CTFs.^139^ In contrast, Aβ_1–40_ had not effect on LTD.^131^

Collectively, these observations reveal that APP processing can modulate both directions of synaptic plasticity, recruiting complex networks of signaling pathways.

### APP regulates inhibitory neurotransmission

**Figure 5.**
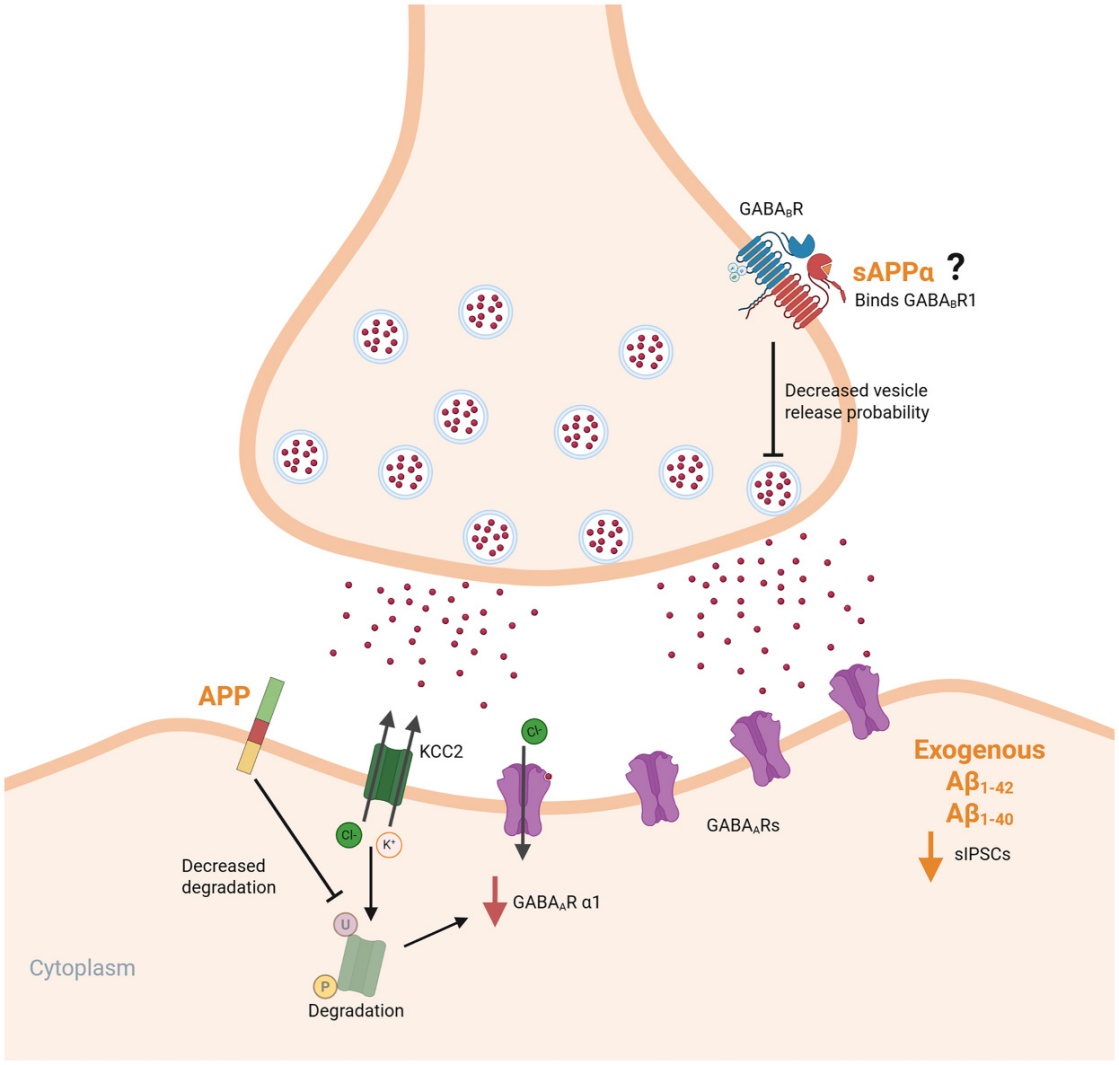
The role of amyloid precursor protein (APP) and its fragments in regulating inhibitory neurotransmission. Full-length APP stabilizes the K-Cl cotransporter 2 (KCC2) at the postsynaptic site, preventing its degradation, which maintains the appropriate chloride gradient necessary for GABAergic signaling. This stabilization of KCC2 also prevents a reduction in the GABA_A_R α1 subunit. Additionally, soluble APPα (sAPPα) might bind to presynaptic GABA_B_ receptors, decreasing the probability of vesicle release and modulating inhibitory transmission. However, this is still controversially discussed in the field. Exogenous application of amyloid-β peptides (Aβ_1−40_ and Aβ_1−42_) has been shown to reduce the frequency of spontaneous inhibitory postsynaptic currents (sIPSCs), although the underlying mechanisms remain unclear. Created in BioRender. Lenz, M. (2025) https://BioRender.com/j97s244.

Inhibitory synapses are regulatory elements in neuronal networks, orchestrating memory formation and network oscillations.^146^ Recent studies have identified a role for APP in modulating inhibitory synapses at the structural and functional level. In APP-deficient tissues, inhibitory synaptic strength was reduced.^35,112,147^ This effect may be due to a decreased expression of GABA_A_ receptor subunits, such as GABA receptor subunit α1, mediated by changes in KCC2 membrane levels.^147^ Here, full-length APP stabilizes KCC2 in membranes by suppressing its ubiquitination and phosphorylation (Figure 5). Moreover, APP can regulate inhibitory synapses through its binding to the synaptic adhesion molecule MDGA1.^148^ MDGA1, which is located at postsynaptic sites, negatively regulates GABAergic synaptic function via *trans*-synaptic interactions with presynaptic APP. The functionality of presynaptic APP is required for inhibitory synaptic integrity, as demonstrated for compartmental knock-outs that show a reduction in the density of the synaptic scaffolding protein gephyrin and diminished inhibitory postsynaptic currents (IPSCs).^148^ These functions of APP in interneuron compartments are associated with altered interneuron excitability, connectivity, and morphology in APP-deficient tissues,^148,149^ which aligns with a reduction of inhibitory neurotransmission. However, in APP/APLP1/APLP2 triple mutant mice, increased inhibitory synaptic transmission has been reported, indicating that complex interactions exist within the APP gene family.^87^

In addition to actions of the full-length protein, APP processing can influence inhibitory neurotransmission and the induction of inhibition-related plasticity.^150^ Exposure to amyloidogenic cleavage products, including both Aβ_1–40_ and Aβ_1–42_, leads to alterations of GABAergic transmission across synaptic and extrasynaptic compartments.^77,106,151^ Increased levels of Aβ_1–42_ are associated with a downregulation of spontaneous IPSCs (sIPSCs), which was also observed upon APP overexpression, potentially resulting from increased APP processing.^151^ Moreover, recent findings suggest that Aβ-driven changes at inhibitory synapses might involve retrograde CB1R signaling.^123^ Upon intracerebral Aβ-injections, previous reports demonstrated a reduction in the number of interneurons that specifically express α7nACh receptors.^77^ Thus, a strong interrelation of Aβ and inhibitory synaptic function is implied.

Furthermore, inhibitory synaptic function is also a target for sAPPα. sAPPα directly binds to the GABA_B_Rα1, thereby suppressing presynaptic vesicle release probability. As a result, frequencies of both inhibitory and excitatory synaptic events are reduced (Figure 5^107^), although these findings remain controversial.^108^

A crosstalk between inhibitory synapses and APP has been demonstrated in transgenic mouse models, mostly investigating the pathogenesis of AD. These models present a heterogeneous picture of inhibitory synaptic transmission: While some models show reduced inhibitory synaptic transmission,^117,152,153^ others display no effects.^154,155^ Notably, in some mouse models, both synaptic transmission and the expression of LTP were restored upon inhibition of astrocytic GABA synthesis.^156^ The heterogeneity of findings can be explained by multiple factors, including differences in experimental designs, genetic models, and/or the age of animals.^157^ These variations may also point towards the complex pathophysiology underlying AD.

## Perspectives

A substantial body of evidence supports the role of APP in regulating neurotransmission across glutamatergic, GABAergic, and cholinergic synapses, each involving distinct downstream signaling pathways. Its influence includes the modulation of synaptic strength, plasticity, and overall network activity, thereby maintaining neural circuit function under physiological conditions.

However, several challenges impede a comprehensive understanding of the physiological role of APP. A significant portion of the current knowledge is derived from disease models carrying genetic mutations or alterations associated with AD. These mouse models usually overexpress human APP carrying disease-associated mutations for promoting plaque formation. Thus, higher levels of APP and its fragments, including Aβ and CTFs, are prevalent in these models. Recent efforts established knock-in mouse models that overcome the limitations of non-physiological overexpression of APP, which is a substantial advancement in the field of AD transgenic mouse models.^158^ However, the heterogeneity of AD models reflects the complexity of AD pathophysiology, making it difficult to identify normal functions of APP from pathological manifestations. Nevertheless, a consensus can be found for its critical involvement in synaptic adhesion, neurotransmitter release modulation, and synaptic plasticity—all essential for proper network function.

Knock-out models lacking APP exhibit deficits in synaptic plasticity mechanisms such as LTP and LTD, as well as homeostatic plasticity, which are foundational processes for learning and memory. However—to the best of our knowledge—no human genetic disease with a loss of APP has been described. Conversely, accumulating the APP gene as observed in Down syndrome (Trisomy 21) leads to pathological brain states with increased risks for the development of AD. In line with these observations, the extracellular accumulation of APP fragments, particularly Aβ peptides, can disrupt plasticity by interfering with synaptic signaling and receptor function. However, Aβ peptides at physiological concentrations enable synaptic plasticity and network homeostasis. These observations demonstrate that both insufficient and excessive levels of APP or its metabolites can impair synaptic function, suggesting dose-dependent regulatory mechanisms. The mechanisms underlying dose-dependent effects, however, warrant further investigation. Therefore, it is a major challenge to understand how APP transitions from a physiological regulator to a contributor to pathology.

In addition to its overall expression levels and regulated processing, the physiological function of APP relies on the appropriate subcellular localization, such as pre- and postsynaptic sites or in synaptic and extrasynaptic compartments. Future work will demonstrate how changes in APP in either pre- or postsynaptic sites influence neural circuits. Disruptions in APP transport mechanisms, such as defects in axonal transport or local protein synthesis, could hamper synaptic function through imbalanced protein contents in pre- and postsynaptic sites, regardless of overall expression levels. Investigating these processes may reveal how altered APP localization, which might come along with protein accumulation or depletion, contributes to synaptic deficits observed in neurodegenerative conditions.

## Conclusion

In conclusion, APP plays a vital role in maintaining synaptic physiology. Its involvement in neurotransmission and plasticity underscores its importance in normal brain function. By further exploring the mechanisms of synaptic localization, distinct pre- and postsynaptic functions, integration into neural circuits, and the network effects of its processing, future work will provide a more comprehensive understanding of synaptic function under both physiological and pathological conditions.

## Supplemental Material

sj-pdf-1-sci-10.1177_00368504251360728 - Supplemental material for A synapse perspective on the function of the amyloid precursor protein Supplemental material, sj-pdf-1-sci-10.1177_00368504251360728 for A synapse perspective on the function of the amyloid precursor protein by Pia Kruse, Amelie Eichler, Larissa Klukas and Maximilian Lenz in Science Progress

sj-pdf-2-sci-10.1177_00368504251360728 - Supplemental material for A synapse perspective on the function of the amyloid precursor protein Supplemental material, sj-pdf-2-sci-10.1177_00368504251360728 for A synapse perspective on the function of the amyloid precursor protein by Pia Kruse, Amelie Eichler, Larissa Klukas and Maximilian Lenz in Science Progress

sj-pdf-3-sci-10.1177_00368504251360728 - Supplemental material for A synapse perspective on the function of the amyloid precursor protein Supplemental material, sj-pdf-3-sci-10.1177_00368504251360728 for A synapse perspective on the function of the amyloid precursor protein by Pia Kruse, Amelie Eichler, Larissa Klukas and Maximilian Lenz in Science Progress
