# Supplementary material for: A synapse perspective on the function of the amyloid precursor protein
Source: Sci Prog. 2025 Jul 30;108(3):00368504251360728. doi: 10.1177/00368504251360728 (PMC12317227; doi:10.1177/00368504251360728)
Supplement: sj-pdf-1-sci-10.1177_00368504251360728 - Supplemental material for A synapse perspective on the function of the amyloid precursor protein [file sj-pdf-1-sci-10.1177_00368504251360728.pdf]

Literature Research for Hippocampus

General information				Investigated Region(s)							Used Model				Investigated Neurotransmission		Experimental Modality			Electrophysiological Assessment		Genetic Modification(s)	Substance or Intervention Investigated
Author	Title	Year	Journal	CA1	CA2	CA3	DG	EC	Subiculum	Primary Hippocampal Cells	In Vivo/Ex Vivo Mouse	In Vivo/Ex Vivo Rat	In Vitro	Human	Excitation	Inhibition	Functional Assessment	Structural Assessment	Behavioral Experiments	Plasticity	Transmission only		
E. Abramov, I. Dolev, H. Fogel, G. D. Ciccotosto, E. Ruff and I. Slutsky	Amyloid-beta as a positive endogenous regulator of release probability at hippocampal synapses	2009	Nat Neurosci 2009 Vol. 12 Issue 12 Pages 1567-76	x								x	x		x	x	x			x		x	x
S. Alfonso, H. W. Kessels, C. C. Banos, T. R. Chan, E. T. Lin, G. Kumaravel, et al.	Synapto-depressive effects of amyloid beta require PICK1	2014	Eur J Neurosci 2014 Vol. 39 Issue 7 Pages 1225-33	x							x		x		x		x	x			x	x	x
A. H. Alhebshi, M. Gotoh and I. Suzuki	Thymoquinone protects cultured rat primary neurons against amyloid β-induced neurotoxicity	2013	Biochem Biophys Res Commun 2013 Vol. 433 Issue 4 Pages 362-7							x			x		x		x				x		x
S. Aliakbari, M. Sayyah, H. Mirzapourdelavar, N. Amini, N. Naghdi and H. G. Pourbadie	Overexpression of protein kinase Mζ in the hippocampal dentate gyrus rescues amyloid-β-induced synaptic dysfunction within entorhinal-hippocampal circuit	2021	Neurobiol Aging 2021 Vol. 101 Pages 160-171				x					x			x		x		x	x			
S. Alves, G. Churlaud, M. Audrain, K. Michaelsen-Preusse, R. Fol, B. Souchet, et al.	Interleukin-2 improves amyloid pathology, synaptic failure and memory in Alzheimer's disease mice	2017	Brain 2017 Vol. 140 Issue 3 Pages 826-842	x							x			x	x		x	x	x	x		x	
A. Androuin, B. Potier, U. V. Nägerl, D. Cattaert, L. Danglot, M. Thierry, et al.	Evidence for altered dendritic spine compartmentalization in Alzheimer's disease and functional effects in a mouse model	2018	Acta Neuropathol 2018 Vol. 135 Issue 6 Pages 839-854	x							x			x	x		x	x	x	x		x	
S. L. Angulo, R. Orman, S. A. Neymotin, L. Liu, L. Buitrago, E. Cepeda-Prado, et al.	Tau and amyloid-related pathologies in the entorhinal cortex have divergent effects in the hippocampal circuit	2017	Neurobiol Dis 2017 Vol. 108 Pages 261-276					x	x		x				x		x	x		x		x	
J. Apelt, A. Kumar and R. Schliebs	Impairment of cholinergic neurotransmission in adult and aged transgenic Tg2576 mouse brain expressing the Swedish mutation of human beta-amyloid precursor protein	2002	Brain Res 2002 Vol. 953 Issue 1-2 Pages 17-30					x			x				x			x			x	x	
J. Arévalo-Serrano, J. M. Sanz-Anquela and A. Gonzalo-Ruiz	Beta-amyloid peptide-induced modifications in alpha7 nicotinic acetylcholine receptor immunoreactivity in the hippocampus of the rat: relationship with GABAergic and calcium-binding proteins perikarya	2008	Brain Res Bull 2008 Vol. 75 Issue 5 Pages 533-44	x	x	x	x					x				x		x			x		
M. Asadbegi, A. Komaki, I. Salehi, P. Yaghmaei, A. Ebrahim-Habibi, S. Shahidi, et al.	Effects of thymol on amyloid-β-induced impairments in hippocampal synaptic plasticity in rats fed a high-fat diet	2018	Brain Res Bull 2018 Vol. 137 Pages 338-350				x					x			x		x			x			x
E. Aso, P. Andrés-Benito and I. Ferrer	Genetic deletion of CB(1) cannabinoid receptors exacerbates the Alzheimer-like symptoms in a transgenic animal model	2018	Biochem Pharmacol 2018 Vol. 157 Pages 210-216		x						x				x	x		x	x		x	x	
A. E. Barry, I. Klyubin, J. M. Mc Donald, A. J. Mably, M. A. Farrell, M. Scott, et al.	Alzheimer's disease brain-derived amyloid-β-mediated inhibition of LTP in vivo is prevented by immunotargeting cellular prion protein	2011	J Neurosci 2011 Vol. 31 Issue 20 Pages 7259-63	x								x			x		x			x			

General information				Investigated Region(s)							Used Model				Investigated Neurotransmission		Experimental Modality			Electrophysiological Assessment		Genetic Modification(s)	Substance or Intervention Investigated
Author	Title	Year	Journal	CA1	CA2	CA3	DG	EC	Subiculum	Primary Hippocampal Cells	In Vivo/Ex Vivo Mouse	In Vivo/Ex Vivo Rat	In Vitro	Human	Excitation	Inhibition	Functional Assessment	Structural Assessment	Behavioral Experiments	Plasticity	Transmission only		
G. Barthet, T. Jordà-Siquier, J. Rumi-Masante, F. Bernadou, U. Müller and C. Mulle	Presenilin-mediated cleavage of APP regulates synaptotagmin-7 and presynaptic plasticity	2018	Nat Commun 2018 Vol. 9 Issue 1 Pages 4780			x	x				x				x		x	x		x		x	
L. Bellingacci, J. Canonichesi, M. Sciaccaluga, A. Megaro, P. Mazzocchetti, M. Di Mauro, et al.	Locally Synthetized 17-β-Estradiol Reverses Amyloid-β-42-Induced Hippocampal Long-Term Potentiation Deficits	2024	Int J Mol Sci 2024 Vol. 25 Issue 3				x					x			x		x			x			x
L. Bellingacci, M. Tallarico, A. Mancini, A. Megaro, C. De Caro, R. Citraro, et al.	Non-competitive AMPA glutamate receptors antagonism by perampanel as a strategy to counteract hippocampal hyper-excitability and cognitive deficits in cerebral amyloidosis	2023	Neuropharmacology 2023 Vol. 225 Pages 109373				x				x				x		x		x	x			x
L. Biasetti, S. Rey, M. Fowler, A. Ratnayaka, K. Fennell, C. Smith, et al.	Elevated amyloid beta disrupts the nanoscale organization and function of synaptic vesicle pools in hippocampal neurons	2023	Cereb Cortex 2023 Vol. 33 Issue 4 Pages 1263-1276	x		x					x		x		x		x	x			x	x	
J. M. Billard and T. Freret	Improved NMDA Receptor Activation by the Secreted Amyloid-Protein Precursor-α in Healthy Aging: A Role for D-Serine?	2022	Int J Mol Sci 2022 Vol. 23 Issue 24	x							x				x		x			x			
Z. Bozso, B. Penke, D. Simon, I. Laczkó, G. Juhász, V. Szegedi, et al.	Controlled in situ preparation of A beta(1-42) oligomers from the isopeptide "iso-A beta(1-42)", physicochemical and biological characterization	2010	Peptides 2010 Vol. 31 Issue 2 Pages 248-56	x								x			x		x		x	x			
H. Breyhan, O. Wirths, K. Duan, A. Marcello, J. Rettig and T. A. Bayer	APP/PS1KI bigenic mice develop early synaptic deficits and hippocampus atrophy	2009	Acta Neuropathol 2009 Vol. 117 Issue 6 Pages 677-85	x							x				x		x			x		x	
J. Brouillette, R. Caillierez, N. Zommer, C. Alves-Pires, I. Benilova, D. Blum, et al.	Neurotoxicity and memory deficits induced by soluble low-molecular-weight amyloid-β1-42 oligomers are revealed in vivo by using a novel animal model	2012	J Neurosci 2012 Vol. 32 Issue 23 Pages 7852-61				x						x		x			x	x		x		
M. Bürge, S. Kratzer, C. Mattusch, C. Hofmann, M. Kreuzer, C. G. Parsons and G. Rammes	The anaesthetic xenon partially restores an amyloid beta-induced impairment in murine hippocampal synaptic plasticity	2019	Neuropharmacology 2019 Vol. 151 Pages 21-32	x							x				x		x			x			x
C. Cai, L. Wang, S. Li, S. Lou, J. L. Luo, D. Y. Fu and T. Chen	Ras Inhibitor Lonafarnib Rescues Structural and Functional Impairments of Synapses of Aβ(1-42) Mice via α7nAChR-Dependent BDNF Upregulation	2022	J Neurosci 2022 Vol. 42 Issue 31 Pages 6090-6107	x							x				x		x	x	x	x			x
H. Y. Cai, C. Hölscher, X. H. Yue, S. X. Zhang, X. H. Wang, F. Qiao, et al.	Lixisenatide rescues spatial memory and synaptic plasticity from amyloid β protein-induced impairments in rats	2014	Neuroscience 2014 Vol. 277 Pages 6-13	x								x			x		x		x	x			x
B. Calabrese, G. M. Shaked, I. V. Tabarean, J. Braga, E. H. Koo and S. Halpain	Rapid, concurrent alterations in pre- and postsynaptic structure induced by naturally-secreted amyloid-beta protein	2007	Mol Cell Neurosci 2007 Vol. 35 Issue 2 Pages 183-93							x			x		x	x	x	x		x			

General information				Investigated Region(s)							Used Model				Investigated Neurotransmission		Experimental Modality			Electrophysiological Assessment		Genetic Modification(s)	Substance or Intervention Investigated
Author	Title	Year	Journal	CA1	CA2	CA3	DG	EC	Subiculum	Primary Hippocampal Cells	In Vivo/Ex Vivo Mouse	In Vivo/Ex Vivo Rat	In Vitro	Human	Excitation	Inhibition	Functional Assessment	Structural Assessment	Behavioral Experiments	Plasticity	Transmission only		
B. Calvo-Flores Guzmán, S. Kim, B. Chawdhary, K. Peppercorn, W. P. Tate, H. J. Waldvogel, et al.	Amyloid-Beta(1-42) -Induced Increase in GABAergic Tonic Conductance in Mouse Hippocampal CA1 Pyramidal Cells	2020	Molecules 2020 Vol. 25 Issue 3	x							x					x	x	x	x		x		
V. Cavallucci, N. Berretta, A. Nobili, R. Nisticò, N. B. Mercuri and M. D'Amelio	Calcineurin inhibition rescues early synaptic plasticity deficits in a mouse model of Alzheimer's disease	2013	Neuromolecular Med 2013 Vol. 15 Issue 3 Pages 541-8	x					x		x				x		x			x		x	x
K. Ceyzériat, L. Ben Haim, A. Denizot, D. Pommier, M. Matos, O. Guillemaud, et al.	Modulation of astrocyte reactivity improves functional deficits in mouse models of Alzheimer's disease	2018	Acta Neuropathol Commun 2018 Vol. 6 Issue 1 Pages 104	x		x					x				x		x	x	x	x		x	
S. Chakroborty, C. Briggs, M. B. Miller, I. Goussakov, C. Schneider, J. Kim, et al.	Stabilizing ER Ca2+ channel function as an early preventative strategy for Alzheimer's disease	2012	PLoS One 2012 Vol. 7 Issue 12 Pages e52056	x							x				x		x	x		x		x	x
S. Chakroborty, J. Kim, C. Schneider, A. R. West and G. E. Stutzmann	Nitric oxide signaling is recruited as a compensatory mechanism for sustaining synaptic plasticity in Alzheimer's disease mice	2015	J Neurosci 2015 Vol. 35 Issue 17 Pages 6893-902	x							x				x		x			x		x	x
L. Chang, W. Cui, Y. Yang, S. Xu, W. Zhou, H. Fu, et al.	Protection against β-amyloid-induced synaptic and memory impairments via altering β-amyloid assembly by bis(heptyl)-cognitin	2015	Sci Rep 2015 Vol. 5 Pages 10256				x			x	x		x		x		x	x	x	x			
L. Chen, K. Yamada, T. Nabeshima and M. Sokabe	alpha7 Nicotinic acetylcholine receptor as a target to rescue deficit in hippocampal LTP induction in beta-amyloid infused rats	2006	Neuropharmacology 2006 Vol. 50 Issue 2 Pages 254-68	x								x			x		x			x			
L. Chen, Z. Zhuang, H. Duan, D. Lv, S. Hong, P. Chen, et al.	Corilagin improves cognitive impairment in APP/PS1 mice by reducing Aβ generation and enhancing synaptic plasticity	2024	Eur J Pharmacol 2024 Vol. 981 Pages 176893	x		x	x				x				x			x	x		x	x	x
M. Chen, J. Wang, J. Jiang, X. Zheng, N. J. Justice, K. Wang, et al.	APP modulates KCC2 expression and function in hippocampal GABAergic inhibition	2017	Elife 2017 Vol. 6	x							x		x			x	x	x			x	x	
Y. Chen and T. Behnisch	The role of γ-secretase in hippocampal synaptic transmission and activity-dependent synaptic plasticity	2013	Neurosci Lett 2013 Vol. 554 Pages 16-21	x								x			x		x			x			x
S. A. Chong, I. Benilova, H. Shaban, B. De Strooper, H. Devijver, D. Moechars, et al.	Synaptic dysfunction in hippocampus of transgenic mouse models of Alzheimer's disease: a multi-electrode array study	2011	Neurobiol Dis 2011 Vol. 44 Issue 3 Pages 284-91	x		x					x				x		x			x		x	
C. Colussi, G. Aceto, C. Ripoli, A. Bertozzi, D. D. Li Puma, E. Paccosi, et al.	Cytoplasmic HDAC4 recovers synaptic function in the 3×Tg mouse model of Alzheimer's disease	2023	Neuropathol Appl Neurobiol 2023 Vol. 49 Issue 1 Pages e12861	x									x		x		x	x			x	x	
D. A. Costello and C. E. Herron	The role of c-Jun N-terminal kinase in the A beta-mediated impairment of LTP and regulation of synaptic transmission in the hippocampus	2004	Neuropharmacology 2004 Vol. 46 Issue 5 Pages 655-62	x								x			x		x			x			x

General information				Investigated Region(s)							Used Model				Investigated Neurotransmission		Experimental Modality			Electrophysiological Assessment		Genetic Modification(s)	Substance or Intervention Investigated
Author	Title	Year	Journal	CA1	CA2	CA3	DG	EC	Subiculum	Primary Hippocampal Cells	In Vivo/Ex Vivo Mouse	In Vivo/Ex Vivo Rat	In Vitro	Human	Excitation	Inhibition	Functional Assessment	Structural Assessment	Behavioral Experiments	Plasticity	Transmission only		
D. A. Costello, D. M. O'Leary and C. E. Herron	Agonists of peroxisome proliferator-activated receptor-gamma attenuate the Abeta-mediated impairment of LTP in the hippocampus in vitro	2005	Neuropharmacology 2005 Vol. 49 Issue 3 Pages 359-66	x								x			x		x			x			x
M. E. Cuevas, H. Haensgen, F. J. Sepúlveda, G. Zegers, J. Roa, C. Opazo and L. G. Aguayo	Soluble Aβ(1-40) peptide increases excitatory neurotransmission and induces epileptiform activity in hippocampal neurons	2011	J Alzheimers Dis 2011 Vol. 23 Issue 4 Pages 673-87							x			x		x	x	x	x			x		
D. M. Cummings, W. Liu, E. Portelius, S. Bayram, M. Yasvoina, S. H. Ho, et al.	First effects of rising amyloid-β in transgenic mouse brain: synaptic transmission and gene expression	2015	Brain 2015 Vol. 138 Issue Pt 7 Pages 1992-2004	x		x	x	x			x				x		x	x		x		x	
A. T. Dao, M. A. Zagaar and K. A. Alkadhi	Moderate Treadmill Exercise Protects Synaptic Plasticity of the Dentate Gyrus and Related Signaling Cascade in a Rat Model of Alzheimer's Disease	2015	Mol Neurobiol 2015 Vol. 52 Issue 3 Pages 1067-1076				x					x			x		x	x		x		x	
D. Del Prete, F. Lombino, X. Liu and L. D'Adamio	APP is cleaved by Bace1 in pre-synaptic vesicles and establishes a pre-synaptic interactome, via its intracellular domain, with molecular complexes that regulate pre-synaptic vesicles functions	2014	PLoS One 2014 Vol. 9 Issue 9 Pages e108576	x							x				x			x			x		
G. Di Rosa, T. Odrijin, R. A. Nixon and O. Arancio	Calpain inhibitors: a treatment for Alzheimer's disease	2002	J Mol Neurosci 2002 Vol. 19 Issue 1-2 Pages 135-41							x	x		x		x		x	x	x		x	x	x
M. C. Dinamarca, M. Di Luca, J. A. Godoy and N. C. Inestrosa	The soluble extracellular fragment of neuroligin-1 targets Aβ oligomers to the postsynaptic region of excitatory synapses	2015	Biochem Biophys Res Commun 2015 Vol. 466 Issue 1 Pages 66-71	x								x	x		x		x	x		x			
G. H. Doherty, D. Beccano-Kelly, S. D. Yan, F. J. Gunn-Moore and J. Harvey	Leptin prevents hippocampal synaptic disruption and neuronal cell death induced by amyloid β	2013	Neurobiol Aging 2013 Vol. 34 Issue 1 Pages 226-37	x						x		x	x		x		x	x		x			x
H. Dong, M. V. Martin, S. Chambers and J. G. Csernansky	Spatial relationship between synapse loss and beta-amyloid deposition in Tg2576 mice	2007	J Comp Neurol 2007 Vol. 500 Issue 2 Pages 311-21	x			x	x			x				x			x			x	x	
H. Dong, C. M. Yuede, C. A. Coughlan, K. M. Murphy and J. G. Csernansky	Effects of donepezil on amyloid-beta and synapse density in the Tg2576 mouse model of Alzheimer's disease	2009	Brain Res 2009 Vol. 1303 Pages 169-78				x				x				x			x			x	x	x
J. Dunot, S. Moreno, C. Gandin, P. A. Pousinha, M. Amici, J. Dupuis, et al.	APP fragment controls both ionotropic and non-ionotropic signaling of NMDA receptors	2024	Neuron 2024 Vol. 112 Issue 16 Pages 2708-2720.e9	x							x	x	x		x		x	x	x	x		x	
M. S. Durakoglugil, Y. Chen, C. L. White, E. T. Kavalali and J. Herz	Reelin signaling antagonizes beta-amyloid at the synapse	2009	Proc Natl Acad Sci U S A 2009 Vol. 106 Issue 37 Pages 15938-43	x							x				x		x			x			x
P. Dutar and B. Potier	Susceptibility to Aβo and TBOA of LTD and Extrasynaptic NMDAR-Dependent Tonic Current in the Aged Rat Hippocampus	2019	Neurochem Res 2019 Vol. 44 Issue 3 Pages 692-702	x								x			x		x			x			
P. Esmaeili Tazangi, S. M. Moosavi, M. Shabani and M. Haghani	Erythropoietin improves synaptic plasticity and memory deficits by decrease of the neurotransmitter release probability in the rat model of Alzheimer's disease	2015	Pharmacol Biochem Behav 2015 Vol. 130 Pages 15-21	x								x			x		x		x	x			x

General information				Investigated Region(s)							Used Model				Investigated Neurotransmission		Experimental Modality			Electrophysiological Assessment		Genetic Modification(s)	Substance or Intervention Investigated
Author	Title	Year	Journal	CA1	CA2	CA3	DG	EC	Subiculum	Primary Hippocampal Cells	In Vivo/Ex Vivo Mouse	In Vivo/Ex Vivo Rat	In Vitro	Human	Excitation	Inhibition	Functional Assessment	Structural Assessment	Behavioral Experiments	Plasticity	Transmission only		
E. Faldini, T. Ahmed, L. Bueé, D. Blum and D. Balschun	Tau- but not Aß -pathology enhances NMDAR-dependent depotentiation in AD-mouse models	2019	Acta Neuropathol Commun 2019 Vol. 7 Issue 1 Pages 202	x							x				x		x			x		x	
T. Fanutza, D. Del Prete, M. J. Ford, P. E. Castillo and L. D'Adamio	APP and APLP2 interact with the synaptic release machinery and facilitate transmitter release at hippocampal synapses	2015	Elife 2015 Vol. 4 Pages e09743	x		x					x				x		x	x		x		x	
I. Ferrer, E. Martí, A. Tortosa and J. Blasi	Dystrophic neurites of senile plaques are defective in proteins involved in exocytosis and neurotransmission	1998	J Neuropathol Exp Neurol 1998 Vol. 57 Issue 3 Pages 218-25		x		x							x	x			x			x		
S. M. Fitzjohn, F. Kuenzi, R. A. Morton, T. W. Rosahl, H. Lewis, D. Smith, et al.	A study of long-term potentiation in transgenic mice over-expressing mutant forms of both amyloid precursor protein and presenilin-1	2010	Mol Brain 2010 Vol. 3 Issue 1 Pages 21	x							x				x		x			x		x	
S. M. Fitzjohn, R. A. Morton, F. Kuenzi, C. H. Davies, G. R. Seabrook and G. L. Collingridge	Similar levels of long-term potentiation in amyloid precursor protein -null and wild-type mice in the CA1 region of picrotoxin treated slices	2000	Neurosci Lett 2000 Vol. 288 Issue 1 Pages 9-12	x							x				x	x	x			x		x	
S. M. Fitzjohn, R. A. Morton, F. Kuenzi, T. W. Rosahl, M. Shearman, H. Lewis, et al	Age-related impairment of synaptic transmission but normal long-term potentiation in transgenic mice that overexpress the human APP695SWE mutant form of amyloid precursor protein	2001	J Neurosci 2001 Vol. 21 Issue 13 Pages 4691-8	x							x				x		x			x		x	
H. Fogel, S. Frere, O. Segev, S. Bharill, I. Shapira, N. Gazit, et al.	APP homodimers transduce an amyloid-β-mediated increase in release probability at excitatory synapses	2014	Cell Rep 2014 Vol. 7 Issue 5 Pages 1560-1576	x		x	x				x		x		x		x	x			x	x	
G. Fonar, B. Polis, D. S. Sams, A. Levi, A. Malka, N. Bal, et al.	Modified Snake α-Neurotoxin Averts β-Amyloid Binding to α7 Nicotinic Acetylcholine Receptor and Reverses Cognitive Deficits in Alzheimer's Disease Mice	2021	Mol Neurobiol 2021 Vol. 58 Issue 5 Pages 2322-2341	x							x		x		x		x		x	x		x	x
D. B. Freir, D. A. Costello and C. E. Herron	A beta 25-35-induced depression of long-term potentiation in area CA1 in vivo and in vitro is attenuated by verapamil	2003	J Neurophysiol 2003 Vol. 89 Issue 6 Pages 3061-9	x								x			x		x			x			x
D. B. Freir, C. Holscher and C. E. Herron	Blockade of long-term potentiation by beta-amyloid peptides in the CA1 region of the rat hippocampus in vivo	2001	J Neurophysiol 2001 Vol. 85 Issue 2 Pages 708-13	x								x			x		x			x			
A. R. Fusilier, J. A. Davis, J. R. Paul, S. D. Yates, L. J. McMeekin, L. K. Goode, et al.	Dysregulated clock gene expression and abnormal diurnal regulation of hippocampal inhibitory transmission and spatial memory in amyloid precursor protein transgenic mice	2021	Neurobiol Dis 2021 Vol. 158 Pages 105454	x							x					x	x	x	x		x	x	
V. A. Gault and C. Hölscher	GLP-1 agonists facilitate hippocampal LTP and reverse the impairment of LTP induced by beta-amyloid	2008	Eur J Pharmacol 2008 Vol. 587 Issue 1-3 Pages 112-7	x								x			x		x			x			x

General information				Investigated Region(s)							Used Model				Investigated Neurotransmission		Experimental Modality			Electrophysiological Assessment		Genetic Modification(s)	Substance or Intervention Investigated
**Author**	**Title**	**Year**	**Journal**	**CA1**	**CA2**	**CA3**	**DG**	**EC**	**Subiculum**	**Primary Hippocampal Cells**	**In Vivo/Ex Vivo Mouse**	**In Vivo/Ex Vivo Rat**	**In Vitro**	**Human**	**Excitation**	**Inhibition**	**Functional Assessment**	**Structural Assessment**	**Behavioral Experiments**	**Plasticity**	**Transmission only**		
V. A. Gault and C. Hölscher	Protease-resistant glucose-dependent insulinotropic polypeptide agonists facilitate hippocampal LTP and reverse the impairment of LTP induced by beta-amyloid	2008	J Neurophysiol 2008 Vol. 99 Issue 4 Pages 1590-5	x								x			x		x			x			x
C. Gauthier-Umaña, J. Muñoz-Cabrera, M. Valderrama, A. Múnera and M. O. Nava-Mesa	Acute Effects of Two Different Species of Amyloid-β on Oscillatory Activity and Synaptic Plasticity in the Commissural CA3-CA1 Circuit of the Hippocampus	2020	Neural Plast 2020 Vol. 2020 Pages 8869526	x		x						x			x		x			x			
S. Gelman, J. Palma, G. Tombaugh and A. Ghavami	Differences in Synaptic Dysfunction Between rTg4510 and APP/PS1 Mouse Models of Alzheimer's Disease	2018	J Alzheimers Dis 2018 Vol. 61 Issue 1 Pages 195-208	x							x				x		x			x		x	
S. Gengler, V. A. Gault, P. Harriott and C. Hölscher	Impairments of hippocampal synaptic plasticity induced by aggregated beta-amyloid (25-35) are dependent on stimulation-protocol and genetic background	2007	Exp Brain Res 2007 Vol. 179 Issue 4 Pages 621-30	x								x			x		x			x			
S. Gengler, A. Hamilton and C. Hölscher	Synaptic plasticity in the hippocampus of a APP/PS1 mouse model of Alzheimer's disease is impaired in old but not young mice	2010	PLoS One 2010 Vol. 5 Issue 3 Pages e9764	x							x				x		x			x		x	
J. Giacchino, J. R. Criado, D. Games and S. Henriksen	In vivo synaptic transmission in young and aged amyloid precursor protein transgenic mice	2000	Brain Res 2000 Vol. 876 Issue 1-2 Pages 185-90	x			x				x				x		x			x		x	
B. Gong, Z. Cao, P. Zheng, O. V. Vitolo, S. Liu, A. Staniszewski, et al.	Ubiquitin hydrolase Uch-L1 rescues beta-amyloid-induced decreases in synaptic function and contextual memory	2006	Cell 2006 Vol. 126 Issue 4 Pages 775-88	x							x				x		x	x	x	x		x	x
B. Gong, O. V. Vitolo, F. Trinchese, S. Liu, M. Shelanski and O. Arancio	Persistent improvement in synaptic and cognitive functions in an Alzheimer mouse model after rolipram treatment	2004	J Clin Invest 2004 Vol. 114 Issue 11 Pages 1624-34	x							x				x		x	x	x	x		x	x
Y. Goto, T. Niidome, H. Hongo, A. Akaike, T. Kihara and H. Sugimoto	Impaired muscarinic regulation of excitatory synaptic transmission in the APPswe/PS1dE9 mouse model of Alzheimer's disease	2008	Eur J Pharmacol 2008 Vol. 583 Issue 1 Pages 84-91	x							x				x		x			x		x	x
Z. Gu and J. L. Yakel	Timing-dependent septal cholinergic induction of dynamic hippocampal synaptic plasticity	2011	Neuron 2011 Vol. 71 Issue 1 Pages 155-65	x								x			x		x			x			
W. Gulisano, M. Melone, D. D. Li Puma, M. R. Tropea, A. Palmeri, O. Arancio, et al.	The effect of amyloid-β peptide on synaptic plasticity and memory is influenced by different isoforms, concentrations, and aggregation status	2018	Neurobiol Aging 2018 Vol. 71 Pages 51-60	x							x				x		x		x	x			
W. Gulisano, M. Melone, C. Ripoli, M. R. Tropea, D. D. Li Puma, S. Giunta, et al.	Neuromodulatory Action of Picomolar Extracellular Aβ42 Oligomers on Presynaptic and Postsynaptic Mechanisms Underlying Synaptic Function and Memory	2019	J Neurosci 2019 Vol. 39 Issue 30 Pages 5986-6000	x							x		x		x		x	x	x	x			

General information				Investigated Region(s)							Used Model				Investigated Neurotransmission		Experimental Modality			Electrophysiological Assessment		Genetic Modification(s)	Substance or Intervention Investigated
Author	Title	Year	Journal	CA1	CA2	CA3	DG	EC	Subiculum	Primary Hippocampal Cells	In Vivo/Ex Vivo Mouse	In Vivo/Ex Vivo Rat	In Vitro	Human	Excitation	Inhibition	Functional Assessment	Structural Assessment	Behavioral Experiments	Plasticity	Transmission only		
I. Gureviciene, S. Ikonen, K. Gurevicius, A. Sarkaki, T. van Groen, R. Pussinen, et al.	Normal induction but accelerated decay of LTP in APP + PS1 transgenic mice	2004	Neurobiol Dis 2004 Vol. 15 Issue 2 Pages 188-95	x			x				x				x		x		x	x		x	
W. N. Han, C. Hölscher, L. Yuan, W. Yang, X. H. Wang, M. N. Wu and J. S. Qi	Liraglutide protects against amyloid-β protein-induced impairment of spatial learning and memory in rats	2013	Neurobiol Aging 2013 Vol. 34 Issue 2 Pages 576-88	x								x			x		x		x	x			x
A. Harmeier, C. Wozny, B. R. Rost, L. M. Munter, H. Hua, O. Georgiev, et al.	Role of amyloid-beta glycine 33 in oligomerization, toxicity, and neuronal plasticity	2009	J Neurosci 2009 Vol. 29 Issue 23 Pages 7582-90	x								x	x		x		x			x			
N. Hashemi-Firouzi, S. Shahidi and S. Soleimani Asl	Chronic stimulation of the serotonergic 5-HT4 receptor modulates amyloid-beta-related impairments in synaptic plasticity and memory deficits in male rats	2021	Brain Res 2021 Vol. 1773 Pages 147701				x					x			x		x	x	x	x			x
Z. He, H. Zhang, X. Li, S. Tu, Z. Wang, S. Han, et al.	The protective effects of Esculentoside A through AMPK in the triple transgenic mouse model of Alzheimer's disease	2023	Phytomedicine 2023 Vol. 109 Pages 154555			x				x	x		x		x			x	x		x	x	x
D. Hefter, M. Kaiser, S. W. Weyer, I. E. Papageorgiou, M. Both, O. Kann, et al.	Amyloid Precursor Protein Protects Neuronal Network Function after Hypoxia via Control of Voltage-Gated Calcium Channels	2016	J Neurosci 2016 Vol. 36 Issue 32 Pages 8356-71	x		x					x				x		x			x		x	x
D. Hermann, M. Both, U. Ebert, G. Gross, H. Schoemaker, A. Draguhn, et al.	Synaptic transmission is impaired prior to plaque formation in amyloid precursor protein-overexpressing mice without altering behaviorally-correlated sharp wave-ripple complexes	2009	Neuroscience 2009 Vol. 162 Issue 4 Pages 1081-90	x							x				x		x			x		x	
D. Hermann, M. Mezler, M. K. Müller, K. Wicke, G. Gross, A. Draguhn, et al.	Synthetic Aβ oligomers (Aβ(1-42) globulomer) modulate presynaptic calcium currents: prevention of Aβ induced synaptic deficits by calcium channel blockers	2013	Eur J Pharmacol 2013 Vol. 702 Issue 1-3 Pages 44-55	x									x		x		x						
M. Hick, U. Herrmann, S. W. Weyer, J. P. Mallm, J. A. Tschäpe, M. Borgers, et al.	Acute function of secreted amyloid precursor protein fragment APPsα in synaptic plasticity	2015	Acta Neuropathol 2015 Vol. 129 Issue 1 Pages 21-37	x							x				x		x	x	x	x		x	
H. Hillen, S. Barghorn, A. Striebinger, B. Labkovsky, R. Müller, V. Nimmrich, et al.	Generation and therapeutic efficacy of highly oligomer-specific beta-amyloid antibodies	2010	J Neurosci 2010 Vol. 30 Issue 31 Pages 10369-79	x						x	x		x		x		x	x	x		x	x	x
E. Hidisoglu, G. Chiantia, C. Franchino, G. Tomagra, M. Giustetto, E. Carbone, et al.	The ryanodine receptor-calstabin interaction stabilizer S107 protects hippocampal neurons from GABAergic synaptic alterations induced by Abeta42 oligomers	2022	J Physiol 2022 Vol. 600 Issue 24 Pages 5295-5309							x			x			x	x			x			
C. Holscher, S. Gengler, V. A. Gault, P. Harriott and H. A. Mallot	Soluble beta-amyloid[25-35] reversibly impairs hippocampal synaptic plasticity and spatial learning	2007	Eur J Pharmacol 2007 Vol. 561 Issue 1-3 Pages 85-90	x								x			x		x		x	x			
J. B. Hoppe, M. Haag, B. J. Whalley, C. G. Salbego and H. Cimarosti	Curcumin protects organotypic hippocampal slice cultures from Aβ1-42-induced synaptic toxicity	2013	Toxicol In Vitro 2013 Vol. 27 Issue 8 Pages 2325-30							x			x		x		x			x			x

General information				Investigated Region(s)							Used Model				Investigated Neurotransmission		Experimental Modality			Electrophysiological Assessment		Genetic Modification(s)	Substance or Intervention Investigated
Author	Title	Year	Journal	CA1	CA2	CA3	DG	EC	Subiculum	Primary Hippocampal Cells	In Vivo/Ex Vivo Mouse	In Vivo/Ex Vivo Rat	In Vitro	Human	Excitation	Inhibition	Functional Assessment	Structural Assessment	Behavioral Experiments	Plasticity	Transmission only		
A. Y. Hsia, E. Masliah, L. McConlogue, G. Q. Yu, G. Tatsuno, K. Hu, et al.	Plaque-independent disruption of neural circuits in Alzheimer's disease mouse models	1999	Proc Natl Acad Sci U S A 1999 Vol. 96 Issue 6 Pages 3228-33	x							x				x		x	x		x		x	
N. W. Hu, I. Klyubin, R. Anwyl and M. J. Rowan	GluN2B subunit-containing NMDA receptor antagonists prevent Abeta-mediated synaptic plasticity disruption in vivo	2009	Proc Natl Acad Sci U S A 2009 Vol. 106 Issue 48 Pages 20504-9	x								x			x		x			x			
N. W. Hu, A. J. Nicoll, D. Zhang, A. J. Mably, T. O'Malley, S. A. Purro, et al.	mGlu5 receptors and cellular prion protein mediate amyloid-β-facilitated synaptic long-term depression in vivo	2014	Nat Commun 2014 Vol. 5 Pages 3374	x								x			x		x			x			x
Y. S. Hu, P. Xu, G. Pigino, S. T. Brady, J. Larson and O. Lazarov	Complex environment experience rescues impaired neurogenesis, enhances synaptic plasticity, and attenuates neuropathology in familial Alzheimer's disease-linked APPswe/PS1DeltaE9 mice	2010	Faseb j 2010 Vol. 24 Issue 6 Pages 1667-81	x			x				x				x		x			x		x	x
S. Huang, H. Tong, M. Lei, M. Zhou, W. Guo, G. Li, et al.	Astrocytic glutamatergic transporters are involved in Aβ-induced synaptic dysfunction	2018	Brain Res 2018 Vol. 1678 Pages 129-137	x							x				x		x			x			x
C. F. M. Huffels, L. M. Osborn, L. A. Hulshof, L. Kooijman, L. Henning, C. Steinhäuser and E. M. Hol	Amyloid-β plaques affect astrocyte Kir4.1 protein expression but not function in the dentate gyrus of APP/PS1 mice	2022	Glia 2022 Vol. 70 Issue 4 Pages 748-767				x				x				x		x				x	x	
N. C. Inestrosa, C. Tapia-Rojas, T. N. Griffith, F. J. Carvajal, M. J. Benito, A. Rivera-Dictter, et al.	Tetrahydrohyperforin prevents cognitive deficit, Aβ deposition, tau phosphorylation and synaptotoxicity in the APPswe/PSEN1ΔE9 model of Alzheimer's disease: a possible effect on APP processing	2011	Transl Psychiatry 2011 Vol. 1 Issue 7 Pages e20							x	x				x		x			x		x	x
T. Isono, N. Yamashita, M. Obara, T. Araki, F. Nakamura, Y. Kamiya, et al.	Amyloid-β_{25-35} induces impairment of cognitive function and long-term potentiation through phosphorylation of collapsin response mediator protein 2	2013	Neurosci Res 2013 Vol. 77 Issue 3 Pages 180-5	x							x				x		x		x	x		x	
P. Jedlicka, M. Owen, M. Vnencak, J. A. Tschäpe, M. Hick, U. C. Müller and T. Deller	Functional consequences of the lack of amyloid precursor protein in the mouse dentate gyrus in vivo	2012	Exp Brain Res 2012 Vol. 217 Issue 3-4 Pages 441-7				x				x				x		x			x		x	
N. Jiang, D. Cupolillo, N. Grosjean, E. Muller, S. Deforges, C. Mulle and T. Amédée	Impaired plasticity of intrinsic excitability in the dentate gyrus alters spike transfer in a mouse model of Alzheimer's disease	2021	Neurobiol Dis 2021 Vol. 154 Pages 105345				x				x				x		x		x	x		x	
S. X. Jin, L. Liu, S. Li, A. L. Meunier and D. J. Selkoe	Aβ oligomers from human brain impair mossy fiber LTP in CA3 of hippocampus, but activating cAMP-PKA and cGMP-PKG prevents this	2022	Neurobiol Dis 2022 Vol. 172 Pages 105816			x					x				x		x			x			
W. Jing, F. Guo, L. Cheng, J. F. Zhang and J. S. Qi	Arginine vasopressin prevents amyloid beta protein-induced impairment of long-term potentiation in rat hippocampus in vivo	2009	Neurosci Lett 2009 Vol. 450 Issue 3 Pages 306-10	x								x			x		x			x			x
T. Jolas, X. S. Zhang, Q. Zhang, G. Wong, R. Del Vecchio, L. Gold and T. Priestley	Long-term potentiation is increased in the CA1 area of the hippocampus of APP(swe/ind) CRND8 mice	2002	Neurobiol Dis 2002 Vol. 11 Issue 3 Pages 394-409	x							x				x	x	x	x		x		x	

General information				Investigated Region(s)							Used Model				Investigated Neurotransmission		Experimental Modality			Electrophysiological Assessment		Genetic Modification(s)	Substance or Intervention Investigated
Author	Title	Year	Journal	CA1	CA2	CA3	DG	EC	Subiculum	Primary Hippocampal Cells	In Vivo/Ex Vivo Mouse	In Vivo/Ex Vivo Rat	In Vitro	Human	Excitation	Inhibition	Functional Assessment	Structural Assessment	Behavioral Experiments	Plasticity	Transmission only		
J. H. Jung, K. An, O. B. Kwon, H. S. Kim and J. H. Kim	Pathway-specific alteration of synaptic plasticity in Tg2576 mice	2011	Mol Cells 2011 Vol. 32 Issue 2 Pages 197-201	x		x					x				x		x			x		x	
K. S. Kaleka and N. Z. Gerges	Neurogranin restores amyloid β-mediated synaptic transmission and long-term potentiation deficits	2016	Exp Neurol 2016 Vol. 277 Pages 115-123	x									x		x		x			x		x	x
F. Kamenetz, T. Tomita, H. Hsieh, G. Seabrook, D. Borchelt, T. Iwatsubo, et al.	APP processing and synaptic function	2003	Neuron 2003 Vol. 37 Issue 6 Pages 925-37	x									x		x		x	x		x		x	
J. Kim, S. Kim, H. Kim, I. W. Hwang, S. Bae, S. Karki, et al.	MDGA1 negatively regulates amyloid precursor protein-mediated synapse inhibition in the hippocampus	2022	Proc Natl Acad Sci U S A 2022 Vol. 119 Issue 4	x							x		x		x	x	x	x	x		x	x	x
J. H. Kim, R. Anwyl, Y. H. Suh, M. B. Djamgoz and M. J. Rowan	Use-dependent effects of amyloidogenic fragments of (beta)-amyloid precursor protein on synaptic plasticity in rat hippocampus in vivo	2001	J Neurosci 2001 Vol. 21 Issue 4 Pages 1327-33	x								x			x		x			x			
R. Kimura, L. Devi and M. Ohno	Partial reduction of BACE1 improves synaptic plasticity, recent and remote memories in Alzheimer's disease transgenic mice	2010	J Neurochem 2010 Vol. 113 Issue 1 Pages 248-61	x							x				x		x		x	x		x	
R. Kimura, D. MacTavish, J. Yang, D. Westaway and J. H. Jhamandas	Beta amyloid-induced depression of hippocampal long-term potentiation is mediated through the amylin receptor	2012	J Neurosci 2012 Vol. 32 Issue 48 Pages 17401-6	x							x				x		x			x		x	x
R. Kimura, D. MacTavish, J. Yang, D. Westaway and J. H. Jhamandas	Pramlintide Antagonizes Beta Amyloid (Aβ)- and Human Amylin-Induced Depression of Hippocampal Long-Term Potentiation	2017	Mol Neurobiol 2017 Vol. 54 Issue 1 Pages 748-754	x							x				x		x			x		x	x
R. Kimura and M. Ohno	Impairments in remote memory stabilization precede hippocampal synaptic and cognitive failures in 5XFAD Alzheimer mouse model	2009	Neurobiol Dis 2009 Vol. 33 Issue 2 Pages 229-35	x							x				x		x		x	x		x	
M. Klevanski, U. Herrmann, S. W. Weyer, R. Fol, N. Cartier, D. P. Wolfer, et al.	The APP Intracellular Domain Is Required for Normal Synaptic Morphology, Synaptic Plasticity, and Hippocampus-Dependent Behavior	2015	J Neurosci 2015 Vol. 35 Issue 49 Pages 16018-33	x							x				x		x	x	x	x		x	
I. Klyubin, T. Ondrejcak, J. Hayes, W. K. Cullen, A. J. Mably, D. M. Walsh and M. J. Rowan	Neurotransmitter receptor and time dependence of the synaptic plasticity disrupting actions of Alzheimer's disease Aβ in vivo	2014	Philos Trans R Soc Lond B Biol Sci 2014 Vol. 369 Issue 1633 Pages 20130147	x								x			x		x			x			x
I. Klyubin, Q. Wang, M. N. Reed, E. A. Irving, N. Upton, J. Hofmeister, et al.	Protection against Aβ-mediated rapid disruption of synaptic plasticity and memory by memantine	2011	Neurobiol Aging 2011 Vol. 32 Issue 4 Pages 614-23				x					x	x		x		x			x			x
M. Knobloch, M. Farinelli, U. Konietzko, R. M. Nitsch and I. M. Mansuy	Abeta oligomer-mediated long-term potentiation impairment involves protein phosphatase 1-dependent mechanisms	2007	J Neurosci 2007 Vol. 27 Issue 29 Pages 7648-53	x							x				x		x			x		x	x
H. Komaki, N. Faraji, A. Komaki, S. Shahidi, F. Etaee, S. Raoufi and F. Mirzaei	Investigation of protective effects of coenzyme Q10 on impaired synaptic plasticity in a male rat model of Alzheimer's disease	2019	Brain Res Bull 2019 Vol. 147 Pages 14-21				x					x			x		x			x			x

General information				Investigated Region(s)							Used Model				Investigated Neurotransmission		Experimental Modality			Electrophysiological Assessment		Genetic Modification(s)	Substance or Intervention Investigated
Author	Title	Year	Journal	CA1	CA2	CA3	DG	EC	Subiculum	Primary Hippocampal Cells	In Vivo/Ex Vivo Mouse	In Vivo/Ex Vivo Rat	In Vitro	Human	Excitation	Inhibition	Functional Assessment	Structural Assessment	Behavioral Experiments	Plasticity	Transmission only		
S. Kootar, M. L. Frandemiche, G. Dhib, X. Mouska, T. Lorivel, G. Poupon-Silvestre, et al.	Identification of an acute functional cross-talk between amyloid-β and glucocorticoid receptors at hippocampal excitatory synapses	2018	Neurobiol Dis 2018 Vol. 118 Pages 117-128	x							x		x		x		x	x		x			x
P. Koppensteiner, F. Trinchese, M. Fà, D. Puzzo, W. Gulisano, S. Yan, et al.	Time-dependent reversal of synaptic plasticity induced by physiological concentrations of oligomeric Aβ42: an early index of Alzheimer's disease	2016	Sci Rep 2016 Vol. 6 Pages 32553	x		x					x		x		x		x	x	x	x			x
D. J. Koss, B. D. Drever, S. Stoppelkamp, G. Riedel and B. Platt	Age-dependent changes in hippocampal synaptic transmission and plasticity in the PLB1Triple Alzheimer mouse	2013	Cell Mol Life Sci 2013 Vol. 70 Issue 14 Pages 2585-601	x							x				x		x	x		x		x	
F. R. Kurudenkandy, M. Zilberter, H. Biverstål, J. Presto, D. Honcharenko, R. Strömberg, et al.	Amyloid-β-induced action potential desynchronization and degradation of hippocampal gamma oscillations is prevented by interference with peptide conformation change and aggregation	2014	J Neurosci 2014 Vol. 34 Issue 34 Pages 11416-25			x					x				x	x	x				x		
S. Lam, A. S. Hérard, S. Boluda, F. Petit, S. Eddarkaoui, K. Cambon, et al.	Pathological changes induced by Alzheimer's brain inoculation in amyloid-beta plaque-bearing mice	2022	Acta Neuropathol Commun 2022 Vol. 10 Issue 1 Pages 112	x				x			x				x			x	x		x	x	
F. Lanté, M. Chafai, E. F. Raymond, A. R. Pereira, X. Mouska, S. Kootar, et al.	Subchronic glucocorticoid receptor inhibition rescues early episodic memory and synaptic plasticity deficits in a mouse model of Alzheimer's disease	2015	Neuropsychopharmacology 2015 Vol. 40 Issue 7 Pages 1772-81	x							x				x		x		x	x		x	x
J. Larson, G. Lynch, D. Games and P. Seubert	Alterations in synaptic transmission and long-term potentiation in hippocampal slices from young and aged PDAPP mice	1999	Brain Res 1999 Vol. 840 Issue 1-2 Pages 23-35	x							x				x		x			x		x	
C. C. Lee, C. P. Chang, C. J. Lin, H. L. Lai, Y. H. Kao, S. J. Cheng, et al.	Adenosine Augmentation Evoked by an ENT1 Inhibitor Improves Memory Impairment and Neuronal Plasticity in the APP/PS1 Mouse Model of Alzheimer's Disease	2018	Mol Neurobiol 2018 Vol. 55 Issue 12 Pages 8936-8952	x							x				x		x	x	x	x		x	x
K. Lee, H. Kim, K. An, O. B. Kwon, S. Park, J. H. Cha, et al.	Replenishment of microRNA-188-5p restores the synaptic and cognitive deficits in 5XFAD Mouse Model of Alzheimer's Disease	2016	Sci Rep 2016 Vol. 6 Pages 34433	x							x		x		x		x	x	x	x		x	
S. H. Lee, J. Kang, A. Ho, H. Watanabe, V. Y. Bolshakov and J. Shen	APP Family Regulates Neuronal Excitability and Synaptic Plasticity but Not Neuronal Survival	2020	Neuron 2020 Vol. 108 Issue 4 Pages 676-690.e8	x							x	x			x		x		x	x		x	
S. H. Lee, K. R. Kim, S. Y. Ryu, S. Son, H. S. Hong, I. Mook-Jung, et al.	Impaired short-term plasticity in mossy fiber synapses caused by mitochondrial dysfunction of dentate granule cells is the earliest synaptic deficit in a mouse model of Alzheimer's disease	2012	J Neurosci 2012 Vol. 32 Issue 17 Pages 5953-63	x		x	x				x				x		x			x		x	
M. Lei, H. Xu, Z. Li, Z. Wang, T. T. O'Malley, D. Zhang, et al.	Soluble Aβ oligomers impair hippocampal LTP by disrupting glutamatergic/GABAergic balance	2016	Neurobiol Dis 2016 Vol. 85 Pages 111-121	x							x				x	x	x			x			x

General information				Investigated Region(s)							Used Model				Investigated Neurotransmission		Experimental Modality			Electrophysiological Assessment		Genetic Modification(s)	Substance or Intervention Investigated
Author	Title	Year	Journal	CA1	CA2	CA3	DG	EC	Subiculum	Primary Hippocampal Cells	In Vivo/Ex Vivo Mouse	In Vivo/Ex Vivo Rat	In Vitro	Human	Excitation	Inhibition	Functional Assessment	Structural Assessment	Behavioral Experiments	Plasticity	Transmission only		
M. Lenz, A. Eichler, P. Kruse, C. Galanis, D. Kleidonas, G. Andrieux, et al.	The Amyloid Precursor Protein Regulates Synaptic Transmission at Medial Perforant Path Synapses	2023	J Neurosci 2023 Vol. 43 Issue 29 Pages 5290-5304				x	x					x		x		x	x		x		x	
S. L. Lesuis, P. J. Lucassen and H. J. Krugers	Early life stress amplifies fear responses and hippocampal synaptic potentiation in the APPswe/PS1dE9 Alzheimer mouse model	2021	Neuroscience 2021 Vol. 454 Pages 151-161	x							x				x		x		x	x		x	x
L. Li, X. K. Tong, M. Hosseini Kahnouei, D. Vallerand, E. Hamel and H. Girouard	Impaired Hippocampal Neurovascular Coupling in a Mouse Model of Alzheimer's Disease	2021	Front Physiol 2021 Vol. 12 Pages 715446	x							x				x		x			x		x	
P. P. Li, W. P. Wang, Z. H. Liu, S. F. Xu, W. W. Lu, L. Wang and X. L. Wang	Potassium 2-(1-hydroxypentyl)-benzoate promotes long-term potentiation in Aβ1-42-injected rats and APP/PS1 transgenic mice	2014	Acta Pharmacol Sin 2014 Vol. 35 Issue 7 Pages 869-78				x				x				x		x	x		x		x	x
S. Li, M. Jin, T. Koeglsperger, N. E. Shepardson, G. M. Shankar and D. J. Selkoe	Soluble Aβ oligomers inhibit long-term potentiation through a mechanism involving excessive activation of extrasynaptic NR2B-containing NMDA receptors	2011	J Neurosci 2011 Vol. 31 Issue 18 Pages 6627-38	x							x		x		x		x	x		x			x
S. Li, M. Jin, D. Zhang, T. Yang, T. Koeglsperger, H. Fu and D. J. Selkoe	Environmental novelty activates β2-adrenergic signaling to prevent the impairment of hippocampal LTP by Aβ oligomers	2013	Neuron 2013 Vol. 77 Issue 5 Pages 929-41	x							x		x		x		x	x		x			x
T. Li, J. J. Jiao, Q. Su, C. Hölscher, J. Zhang, X. D. Yan, et al.	A GLP-1/GIP/Gcg receptor triagonist improves memory behavior, as well as synaptic transmission, neuronal excitability and Ca(2+) homeostasis in 3xTg-AD mice	2020	Neuropharmacology 2020 Vol. 170 Pages 108042	x							x				x		x	x	x	x		x	x
W. Li, J. Yu, Y. Liu, X. Huang, N. Abumaria, Y. Zhu, et al.	Elevation of brain magnesium prevents synaptic loss and reverses cognitive deficits in Alzheimer's disease mouse model	2014	Mol Brain 2014 Vol. 7 Pages 65	x			x				x				x		x	x	x		x	x	x
Y. Li, K. Zhu, N. Li, X. Wang, X. Xiao, L. Li, et al.	Reversible GABAergic dysfunction involved in hippocampal hyperactivity predicts early-stage Alzheimer disease in a mouse model	2021	Alzheimers Res Ther 2021 Vol. 13 Issue 1 Pages 114	x							x				x	x	x	x	x		x	x	x
L. Liu, I. J. Orozco, E. Planel, Y. Wen, A. Bretteville, P. Krishnamurthy, et al.	A transgenic rat that develops Alzheimer's disease-like amyloid pathology, deficits in synaptic plasticity and cognitive impairment	2008	Neurobiol Dis 2008 Vol. 31 Issue 1 Pages 46-57	x								x			x		x		x	x		x	
S. J. Liu, R. Gasperini, L. Foa and D. H. Small	Amyloid-beta decreases cell-surface AMPA receptors by increasing intracellular calcium and phosphorylation of GluR2	2010	J Alzheimers Dis 2010 Vol. 21 Issue 2 Pages 655-66							x			x		x		x	x			x		x
S. Ludewig, U. Herrmann, K. Michaelsen-Preusse, K. Metzdorf, J. Just, C. Bold, et al.	APPsα rescues impaired Ca(2+) homeostasis in APP- and APLP2-deficient hippocampal neurons	2021	Proc Natl Acad Sci U S A 2021 Vol. 118 Issue 26							x			x		x		x	x		x		x	

General information				Investigated Region(s)							Used Model				Investigated Neurotransmission		Experimental Modality			Electrophysiological Assessment		Genetic Modification(s)	Substance or Intervention Investigated
Author	Title	Year	Journal	CA1	CA2	CA3	DG	EC	Subiculum	Primary Hippocampal Cells	In Vivo/Ex Vivo Mouse	In Vivo/Ex Vivo Rat	In Vitro	Human	Excitation	Inhibition	Functional Assessment	Structural Assessment	Behavioral Experiments	Plasticity	Transmission only		
H. Ma, S. Lesné, L. Kotilinek, J. V. Steidl-Nichols, M. Sherman, L. Younkin, et al.	Involvement of beta-site APP cleaving enzyme 1 (BACE1) in amyloid precursor protein-mediated enhancement of memory and activity-dependent synaptic plasticity	2007	Proc Natl Acad Sci U S A 2007 Vol. 104 Issue 19 Pages 8167-72	x							x				x		x		x	x		x	
T. Ma, Y. Chen, V. Vingtdeux, H. Zhao, B. Viollet, P. Marambaud and E. Klann	Inhibition of AMP-activated protein kinase signaling alleviates impairments in hippocampal synaptic plasticity induced by amyloid β	2014	J Neurosci 2014 Vol. 34 Issue 36 Pages 12230-8	x							x			x	x		x	x		x		x	x
T. Ma, X. Du, J. E. Pick, G. Sui, M. Brownlee and E. Klann	Glucagon-like peptide-1 cleavage product GLP-1(9-36) amide rescues synaptic plasticity and memory deficits in Alzheimer's disease model mice	2012	J Neurosci 2012 Vol. 32 Issue 40 Pages 13701-8	x		x					x				x		x			x		x	x
I. Maezawa, B. Zou, J. Di Lucente, W. S. Cao, C. Pascual, S. Weerasekara, et al.	The Anti-Amyloid-β and Neuroprotective Properties of a Novel Tricyclic Pyrone Molecule	2017	J Alzheimers Dis 2017 Vol. 58 Issue 2 Pages 559-574	x							x				x		x		x	x		x	x
M. Manczak, R. Kandimalla, X. Yin and P. H. Reddy	Hippocampal mutant APP and amyloid beta-induced cognitive decline, dendritic spine loss, defective autophagy, mitophagy and mitochondrial abnormalities in a mouse model of Alzheimer's disease	2018	Hum Mol Genet 2018 Vol. 27 Issue 8 Pages 1332-1342	x							x				x			x	x		x	x	
D. Mango and R. Nisticò	Role of ASIC1a in Aβ-induced synaptic alterations in the hippocampus	2018	Pharmacol Res 2018 Vol. 131 Pages 61-65	x							x				x		x			x		x	x
A. Martín-Belmonte, C. Aguado, R. Alfaro-Ruiz, J. L. Albasanz, M. Martín, A. E. Moreno-Martínez, et al.	The Density of Group I mGlu(5) Receptors Is Reduced along the Neuronal Surface of Hippocampal Cells in a Mouse Model of Alzheimer's Disease	2021	Int J Mol Sci 2021 Vol. 22 Issue 11	x			x				x				x			x			x	x	
A. Martín-Belmonte, C. Aguado, R. Alfaro-Ruíz, A. E. Moreno-Martínez, L. de la Ossa, J. Martínez-Hernández, et al.	Reduction in the neuronal surface of post and presynaptic GABA(B) receptors in the hippocampus in a mouse model of Alzheimer's disease	2020	Brain Pathol 2020 Vol. 30 Issue 3 Pages 554-575	x							x					x		x			x	x	
A. Martín-Belmonte, C. Aguado, R. Alfaro-Ruíz, A. E. Moreno-Martínez, L. de la Ossa, J. Martínez-Hernández, et al.	Density of GABA(B) Receptors Is Reduced in Granule Cells of the Hippocampus in a Mouse Model of Alzheimer's Disease	2020	Int J Mol Sci 2020 Vol. 21 Issue 7				x				x					x		x			x	x	
I. V. Martins, J. Rivers-Auty, S. M. Allan and C. B. Lawrence	Mitochondrial Abnormalities and Synaptic Loss Underlie Memory Deficits Seen in Mouse Models of Obesity and Alzheimer's Disease	2017	J Alzheimers Dis 2017 Vol. 55 Issue 3 Pages 915-932							x	x				x			x	x		x	x	x
P. L. McClean, J. Jalewa and C. Hölscher	Prophylactic liraglutide treatment prevents amyloid plaque deposition, chronic inflammation and memory impairment in APP/PS1 mice	2015	Behav Brain Res 2015 Vol. 293 Pages 96-106	x							x				x		x	x	x	x		x	x

General information				Investigated Region(s)							Used Model				Investigated Neurotransmission		Experimental Modality			Electrophysiological Assessment		Genetic Modification(s)	Substance or Intervention Investigated
Author	Title	Year	Journal	CA1	CA2	CA3	DG	EC	Subiculum	Primary Hippocampal Cells	In Vivo/Ex Vivo Mouse	In Vivo/Ex Vivo Rat	In Vitro	Human	Excitation	Inhibition	Functional Assessment	Structural Assessment	Behavioral Experiments	Plasticity	Transmission only		
E. Medawar, T. A. Benway, W. Liu, T. A. Hanan, P. Haslehurst, O. T. James, et al.	Effects of rising amyloidβ levels on hippocampal synaptic transmission, microglial response and cognition in APP(Swe)/PSEN1(M146V) transgenic mice	2019	EBioMedicine 2019 Vol. 39 Pages 422-435	x							x				x		x		x	x		x	
A. Mehr, M. Hick, S. Ludewig, M. Müller, U. Herrmann, J. von Engelhardt, et al.	Lack of APP and APLP2 in GABAergic Forebrain Neurons Impairs Synaptic Plasticity and Cognition	2020	Cereb Cortex 2020 Vol. 30 Issue 7 Pages 4044-4063	x							x		x		x	x	x	x	x	x		x	
Y. Meng, L. Ding, H. Zhang, W. Yin, Y. Yan and Y. Cao	Immunization of Tg-APPswe/PSEN1dE9 mice with Aβ3-10-KLH vaccine prevents synaptic deficits of Alzheimer's disease	2017	Behav Brain Res 2017 Vol. 332 Pages 64-70	x							x				x			x			x	x	x
B. Midthune, S. H. Tyan, J. J. Walsh, F. Sarsoza, S. Eggert, P. R. Hof, et al.	Deletion of the amyloid precursor-like protein 2 (APLP2) does not affect hippocampal neuron morphology or function	2012	Mol Cell Neurosci 2012 Vol. 49 Issue 4 Pages 448-55	x							x		x		x		x	x		x		x	
S. S. Min, J. An, J. H. Lee, G. H. Seol, J. H. Im, H. S. Kim, et al.	Neuregulin-1 prevents amyloid β-induced impairment of long-term potentiation in hippocampal slices via ErbB4	2011	Neurosci Lett 2011 Vol. 505 Issue 1 Pages 6-9	x							x				x		x			x			x
N. A. Morgenstern, D. Giacomini, G. Lombardi, E. M. Castaño and A. F. Schinder	Delayed dendritic development in newly generated dentate granule cells by cell-autonomous expression of the amyloid precursor protein	2013	Mol Cell Neurosci 2013 Vol. 56 Pages 298-306				x				x				x		x	x			x	x	
M. K. Müller, E. Jacobi, K. Sakimura, R. Malinow and J. von Engelhardt	NMDA receptors mediate synaptic depression, but not spine loss in the dentate gyrus of adult amyloid Beta (Aβ) overexpressing mice	2018	Acta Neuropathol Commun 2018 Vol. 6 Issue 1 Pages 110				x				x				x			x			x	x	
T. Nagata, T. Tomiyama, H. Mori, T. Yaguchi and T. Nishizaki	DCP-LA neutralizes mutant amyloid beta peptide-induced impairment of long-term potentiation and spatial learning	2010	Behav Brain Res 2010 Vol. 206 Issue 1 Pages 151-4	x								x			x		x		x	x			x
Y. Nakagami, S. Nishimura, T. Murasugi, T. Kubo, I. Kaneko, M. Meguro, et al.	A novel compound RS-0466 reverses beta-amyloid-induced cytotoxicity through the Akt signaling pathway in vitro	2002	Eur J Pharmacol 2002 Vol. 457 Issue 1 Pages 11-7	x								x	x		x		x			x			x
N. Nakanishi, S. D. Ryan, X. Zhang, A. Khan, T. Holland, E. G. Cho, et al.	Synaptic protein α1-takusan mitigates amyloid-β-induced synaptic loss via interaction with tau and postsynaptic density-95 at postsynaptic sites	2013	J Neurosci 2013 Vol. 33 Issue 35 Pages 14170-83							x			x		x		x	x			x	x	x
S. P. Navabi, A. Sarkaki, E. Mansouri, M. Badavi, A. Ghadiri and Y. Farbood	The effects of betulinic acid on neurobehavioral activity, electrophysiology and histological changes in an animal model of the Alzheimer's disease	2018	Behav Brain Res 2018 Vol. 337 Pages 99-106				x					x			x		x		x	x			x
M. N. Nenov, F. Laezza, S. J. Haidacher, Y. Zhao, R. G. Sadygov, J. M. Starkey, et al.	Cognitive enhancing treatment with a PPARγ agonist normalizes dentate granule cell presynaptic function in Tg2576 APP mice	2014	J Neurosci 2014 Vol. 34 Issue 3 Pages 1028-36				x				x				x		x				x	x	

General information				Investigated Region(s)							Used Model				Investigated Neurotransmission		Experimental Modality			Electrophysiological Assessment		Genetic Modification(s)	Substance or Intervention Investigated
Author	Title	Year	Journal	CA1	CA2	CA3	DG	EC	Subiculum	Primary Hippocampal Cells	In Vivo/Ex Vivo Mouse	In Vivo/Ex Vivo Rat	In Vitro	Human	Excitation	Inhibition	Functional Assessment	Structural Assessment	Behavioral Experiments	Plasticity	Transmission only		
V. Nimmrich, C. Grimm, A. Draguhn, S. Barghorn, A. Lehmann, H. Schoemaker, et al.	Amyloid beta oligomers (A beta(1-42) globulomer) suppress spontaneous synaptic activity by inhibition of P/Q-type calcium currents	2008	J Neurosci 2008 Vol. 28 Issue 4 Pages 788-97							x			x		x	x	x				x		x
V. Nimmrich, K. G. Reymann, M. Strassburger, U. H. Schöder, G. Gross, A. Hahn, et al.	Inhibition of calpain prevents NMDA-induced cell death and beta-amyloid-induced synaptic dysfunction in hippocampal slice cultures	2010	Br J Pharmacol 2010 Vol. 159 Issue 7 Pages 1523-31	x								x	x		x		x				x		x
I. Nomura, N. Kato, T. Kita and H. Takechi	Mechanism of impairment of long-term potentiation by amyloid beta is independent of NMDA receptors or voltage-dependent calcium channels in hippocampal CA1 pyramidal neurons	2005	Neurosci Lett 2005 Vol. 391 Issue 1-2 Pages 1-6	x								x			x		x			x			
I. Nomura, H. Takechi and N. Kato	Intraneuronally injected amyloid β inhibits long-term potentiation in rat hippocampal slices	2012	J Neurophysiol 2012 Vol. 107 Issue 9 Pages 2526-31	x								x			x		x			x			x
E. O'Hare, R. Jeggo, E. M. Kim, B. Barbour, J. S. Walczak, P. Palmer, et al.	Lack of support for bexarotene as a treatment for Alzheimer's disease	2016	Neuropharmacology 2016 Vol. 100 Pages 124-30	x							x	x			x		x		x	x		x	x
T. Ochiishi, M. Kaku, K. Kiyosue, M. Doi, T. Urabe, N. Hattori, et al.	New Alzheimer's disease model mouse specialized for analyzing the function and toxicity of intraneuronal Amyloid β oligomers	2019	Sci Rep 2019 Vol. 9 Issue 1 Pages 17368	x	x						x				x		x	x	x	x		x	
O. J. Olajide and C. A. Chapman	Amyloid-β (1-42) peptide induces rapid NMDA receptor-dependent alterations at glutamatergic synapses in the entorhinal cortex	2021	Neurobiol Aging 2021 Vol. 105 Pages 296-309					x				x			x		x	x			x		
T. G. Oliveira, R. B. Chan, H. Tian, M. Laredo, G. Shui, A. Staniszewski, et al.	Phospholipase d2 ablation ameliorates Alzheimer's disease-linked synaptic dysfunction and cognitive deficits	2010	J Neurosci 2010 Vol. 30 Issue 49 Pages 16419-28	x						x	x		x		x		x		x	x		x	
K. M. Olsen and M. Sheng	NMDA receptors and BAX are essential for Aβ impairment of LTP	2012	Sci Rep 2012 Vol. 2 Pages 225	x							x				x		x			x		x	x
T. Ondrejcak, I. Klyubin, N. W. Hu, T. T. O'Malley, G. T. Corbett, R. Winters, et al.	Tau and Amyloid β Protein in Patient-Derived Aqueous Brain Extracts Act Concomitantly to Disrupt Long-Term Potentiation in Vivo	2023	J Neurosci 2023 Vol. 43 Issue 32 Pages 5870-5879	x								x			x		x			x			
T. Ondrejcak, Q. Wang, J. N. Kew, D. J. Virley, N. Upton, R. Anwyl and M. J. Rowan	Activation of α7 nicotinic acetylcholine receptors persistently enhances hippocampal synaptic transmission and prevents Aß-mediated inhibition of LTP in the rat hippocampus	2012	Eur J Pharmacol 2012 Vol. 677 Issue 1-3 Pages 63-70				x					x			x		x			x			x
A. L. Orr, J. E. Hanson, D. Li, A. Klotz, S. Wright, D. Schenk, et al.	β-Amyloid inhibits E-S potentiation through suppression of cannabinoid receptor 1-dependent synaptic disinhibition	2014	Neuron 2014 Vol. 82 Issue 6 Pages 1334-45	x								x			x	x	x			x			
T. Oyelami, A. Bondt, I. V. den Wyngaert, K. V. Hoorde, L. Hoskens, H. Shaban, et al.	Age-dependent concomitant changes in synaptic dysfunction and GABAergic pathway in the APP/PS1 mouse model	2016	Acta Neurobiol Exp (Wars) 2016 Vol. 76 Issue 4 Pages 282-293	x							x				x	x	x			x		x	

General information				Investigated Region(s)							Used Model				Investigated Neurotransmission		Experimental Modality			Electrophysiological Assessment		Genetic Modification(s)	Substance or Intervention Investigated
**Author**	**Title**	**Year**	**Journal**	**CA1**	**CA2**	**CA3**	**DG**	**EC**	**Subiculum**	**Primary Hippocampal Cells**	**In Vivo/Ex Vivo Mouse**	**In Vivo/Ex Vivo Rat**	**In Vitro**	**Human**	**Excitation**	**Inhibition**	**Functional Assessment**	**Structural Assessment**	**Behavioral Experiments**	**Plasticity**	**Transmission only**		
J. J. Palop, J. Chin, E. D. Roberson, J. Wang, M. T. Thwin, N. Bien-Ly, et al.	Aberrant excitatory neuronal activity and compensatory remodeling of inhibitory hippocampal circuits in mouse models of Alzheimer's disease	2007	Neuron 2007 Vol. 55 Issue 5 Pages 697-711				x				x				x	x	x		x	x		x	
J. J. Palop, L. Mucke and E. D. Roberson	Quantifying biomarkers of cognitive dysfunction and neuronal network hyperexcitability in mouse models of Alzheimer's disease: depletion of calcium-dependent proteins and inhibitory hippocampal remodeling	2011	Methods Mol Biol 2011 Vol. 670 Pages 245-62				x				x				x			x			x		
J. Parodi, F. J. Sepúlveda, J. Roa, C. Opazo, N. C. Inestrosa and L. G. Aguayo	Beta-amyloid causes depletion of synaptic vesicles leading to neurotransmission failure	2010	J Biol Chem 2010 Vol. 285 Issue 4 Pages 2506-14							x			x		x	x	x	x			x		x
M. Pascual-Lucas, S. Viana da Silva, M. Di Scala, C. Garcia-Barroso, G. González-Aseguinolaza, C. Mulle, et al.	Insulin-like growth factor 2 reverses memory and synaptic deficits in APP transgenic mice	2014	EMBO Mol Med 2014 Vol. 6 Issue 10 Pages 1246-62	x		x				x	x		x	x	x		x	x	x		x	x	x
A. Patel, R. Kimura, W. Fu, R. Soudy, D. MacTavish, D. Westaway, et al.	Genetic Depletion of Amylin/Calcitonin Receptors Improves Memory and Learning in Transgenic Alzheimer's Disease Mouse Models	2021	Mol Neurobiol 2021 Vol. 58 Issue 10 Pages 5369-5382	x							x				x		x		x	x		x	
R. Piacentini, D. D. Li Puma, M. Mainardi, G. Lazzarino, B. Tavazzi, O. Arancio and C. Grassi	Reduced gliotransmitter release from astrocytes mediates tau-induced synaptic dysfunction in cultured hippocampal neurons	2017	Glia 2017 Vol. 65 Issue 8 Pages 1302-1316							x			x		x		x	x			x		x
G. Piccioni, N. Maisto, A. d'Ettorre, G. Strimpakos, R. Nisticò, V. Triaca and D. Mango	Switch to phagocytic microglia by CSFR1 inhibition drives amyloid-beta clearance from glutamatergic terminals rescuing LTP in acute hippocampal slices	2024	Transl Psychiatry 2024 Vol. 14 Issue 1 Pages 338	x							x				x		x			x			x
B. Portal, M. Södergren, I. B. T. Parés, R. Giraud, N. G. Metzendorf, G. Hultqvist, et al.	Early Astrocytic Dysfunction Is Associated with Mistuned Synapses as well as Anxiety and Depressive-Like Behavior in the AppNL-F Mouse Model of Alzheimer's Disease	2024	J Alzheimers Dis 2024 Vol. 100 Issue 3 Pages 1017-1037	x							x				x	x	x		x	x		x	x
H. G. Pourbadie, N. Naderi, N. Mehranfard, M. Janahmadi, F. Khodagholi and F. Motamedi	Preventing effect of L-type calcium channel blockade on electrophysiological alterations in dentate gyrus granule cells induced by entorhinal amyloid pathology	2015	PLoS One 2015 Vol. 10 Issue 2 Pages e0117555				x					x			x	x	x				x		x
P. A. Pousinha, X. Mouska, E. F. Raymond, C. Gwizdek, G. Dhib, G. Poupon, et al.	Physiological and pathophysiological control of synaptic GluN2B-NMDA receptors by the C-terminal domain of amyloid precursor protein	2017	Elife 2017 Vol. 6	x								x	x		x		x			x		x	
L. Pradier, V. Blanchard-Brégeon, A. Bohme, T. Debeir, J. Menager, P. Benoit, et al.	SAR228810: an antibody for protofibrillar amyloid β peptide designed to reduce the risk of amyloid-related imaging abnormalities (ARIA)	2018	Alzheimers Res Ther 2018 Vol. 10 Issue 1 Pages 117	x							x		x		x		x				x		x

General information				Investigated Region(s)							Used Model				Investigated Neurotransmission		Experimental Modality			Electrophysiological Assessment		Genetic Modification(s)	Substance or Intervention Investigated
Author	Title	Year	Journal	CA1	CA2	CA3	DG	EC	Subiculum	Primary Hippocampal Cells	In Vivo/Ex Vivo Mouse	In Vivo/Ex Vivo Rat	In Vitro	Human	Excitation	Inhibition	Functional Assessment	Structural Assessment	Behavioral Experiments	Plasticity	Transmission only		
K. A. Price, M. Varghese, A. Sowa, F. Yuk, H. Brautigam, M. E. Ehrlich and D. L. Dickstein	Altered synaptic structure in the hippocampus in a mouse model of Alzheimer's disease with soluble amyloid-β oligomers and no plaque pathology	2014	Mol Neurodegener 2014 Vol. 9 Pages 41	x							x				x			x			x	x	
C. Priller, T. Bauer, G. Mitteregger, B. Krebs, H. A. Kretzschmar and J. Herms	Synapse formation and function is modulated by the amyloid precursor protein	2006	J Neurosci 2006 Vol. 26 Issue 27 Pages 7212-21	x						x	x		x		x		x	x		x		x	
C. Priller, G. Mitteregger, S. Paluch, N. Vassallo, M. Staufenbiel, H. A. Kretzschmar, et al.	Excitatory synaptic transmission is depressed in cultured hippocampal neurons of APP/PS1 mice	2009	Neurobiol Aging 2009 Vol. 30 Issue 8 Pages 1227-37							x			x		x		x	x			x	x	
D. Puzzo, L. Privitera, E. Leznik, M. Fà, A. Staniszewski, A. Palmeri and O. Arancio	Picomolar amyloid-beta positively modulates synaptic plasticity and memory in hippocampus	2008	J Neurosci 2008 Vol. 28 Issue 53 Pages 14537-45	x							x				x		x		x	x			
Y. Qi, I. Klyubin, S. C. Harney, N. Hu, W. K. Cullen, M. K. Grant, et al.	Longitudinal testing of hippocampal plasticity reveals the onset and maintenance of endogenous human Aß-induced synaptic dysfunction in individual freely behaving pre-plaque transgenic rats: rapid reversal by anti-Aß agents	2014	Acta Neuropathol Commun 2014 Vol. 2 Pages 175	x								x			x		x			x		x	x
A. E. Ramírez, C. R. Pacheco, L. G. Aguayo and C. M. Opazo	Rapamycin protects against Aβ-induced synaptotoxicity by increasing presynaptic activity in hippocampal neurons	2014	Biochim Biophys Acta 2014 Vol. 1842 Issue 9 Pages 1495-501							x			x		x		x				x		x
G. Rammes, A. Hasenjäger, K. Sroka-Saidi, J. M. Deussing and C. G. Parsons	Therapeutic significance of NR2B-containing NMDA receptors and mGluR5 metabotropic glutamate receptors in mediating the synaptotoxic effects of β-amyloid oligomers on long-term potentiation (LTP) in murine hippocampal slices	2011	Neuropharmacology 2011 Vol. 60 Issue 6 Pages 982-90	x							x				x		x			x			
G. Rammes, F. Seeser, K. Mattusch, K. Zhu, L. Haas, M. Kummer, et al.	The NMDA receptor antagonist Radiprodil reverses the synaptotoxic effects of different amyloid-beta (Aβ) species on long-term potentiation (LTP)	2018	Neuropharmacology 2018 Vol. 140 Pages 184-192	x							x				x		x	x		x			x
S. K. Rao, J. M. Ross, F. E. Harrison, A. Bernardo, R. S. Reiserer, R. S. Reiserer, et al.	Differential proteomic and behavioral effects of long-term voluntary exercise in wild-type and APP-overexpressing transgenics	2015	Neurobiol Dis 2015 Vol. 78 Pages 45-55							x	x				x	x		x	x		x	x	x
C. R. Raymond, D. R. Ireland and W. C. Abraham	NMDA receptor regulation by amyloid-beta does not account for its inhibition of LTP in rat hippocampus	2003	Brain Res 2003 Vol. 968 Issue 2 Pages 263-72	x								x			x		x			x			
Z. Rezaei Asl, G. Sepehri and M. Salami	Probiotic treatment improves the impaired spatial cognitive performance and restores synaptic plasticity in an animal model of Alzheimer's disease	2019	Behav Brain Res 2019 Vol. 376 Pages 112183	x								x			x		x		x	x			x
H. C. Rice, D. de Malmazet, A. Schreurs, S. Frere, I. Van Molle, A. N. Volkov, et al.	Secreted amyloid-β precursor protein functions as a GABA(B)R1a ligand to modulate synaptic transmission	2019	Science 2019 Vol. 363 Issue 6423	x							x		x		x	x	x			x			x

General information				Investigated Region(s)							Used Model				Investigated Neurotransmission		Experimental Modality			Electrophysiological Assessment		Genetic Modification(s)	Substance or Intervention Investigated
Author	Title	Year	Journal	CA1	CA2	CA3	DG	EC	Subiculum	Primary Hippocampal Cells	In Vivo/Ex Vivo Mouse	In Vivo/Ex Vivo Rat	In Vitro	Human	Excitation	Inhibition	Functional Assessment	Structural Assessment	Behavioral Experiments	Plasticity	Transmission only		
J. G. Richards, G. A. Higgins, A. M. Ouagazzal, L. Ozmen, J. N. Kew, B. Bohrmann, et al.	PS2APP transgenic mice, coexpressing hPS2mut and hAPPswe, show age-related cognitive deficits associated with discrete brain amyloid deposition and inflammation	2003	J Neurosci 2003 Vol. 23 Issue 26 Pages 8989-9003				x		x		x				x		x	x	x	x		x	
K. Richetin, P. Petsophonsakul, L. Roybon, B. P. Guiard and C. Rampon	Differential alteration of hippocampal function and plasticity in females and males of the APPxPS1 mouse model of Alzheimer's disease	2017	Neurobiol Aging 2017 Vol. 57 Pages 220-231				x				x				x		x		x	x		x	
M. C. Richter, S. Ludewig, A. Winschel, T. Abel, C. Bold, L. R. Salzburger, et al.	Distinct in vivo roles of secreted APP ectodomain variants APPsα and APPsβ in regulation of spine density, synaptic plasticity, and cognition	2018	Embo j 2018 Vol. 37 Issue 11	x							x		x		x		x	x	x	x		x	x
U. M. Ricoy, P. Mao, M. Manczak, P. H. Reddy and M. E. Frerking	A transgenic mouse model for Alzheimer's disease has impaired synaptic gain but normal synaptic dynamics	2011	Neurosci Lett 2011 Vol. 500 Issue 3 Pages 212-5	x							x				x		x			x		x	
C. Ripoli, S. Cocco, D. D. Li Puma, R. Piacentini, A. Mastrodonato, F. Scala, et al.	Intracellular accumulation of amyloid-β (Aβ) protein plays a major role in Aβ-induced alterations of glutamatergic synaptic transmission and plasticity	2014	J Neurosci 2014 Vol. 34 Issue 38 Pages 12893-903	x							x		x		x		x	x		x			
E. D. Roberson, B. Halabisky, J. W. Yoo, J. Yao, J. Chin, F. Yan, et al.	Amyloid-β/Fyn-induced synaptic, network, and cognitive impairments depend on tau levels in multiple mouse models of Alzheimer's disease	2011	J Neurosci 2011 Vol. 31 Issue 2 Pages 700-11	x			x				x				x	x	x	x	x	x		x	
J. P. Roberts, S. A. Stokoe, M. F. Sathler, R. A. Nichols and S. Kim	Selective coactivation of α7- and α4β2-nicotinic acetylcholine receptors reverses beta-amyloid-induced synaptic dysfunction	2021	J Biol Chem 2021 Vol. 296 Pages 100402							x			x		x		x	x		x			
S. Roder, L. Danober, M. F. Pozza, K. Lingenhoehl, K. H. Wiederhold and H. R. Olpe	Electrophysiological studies on the hippocampus and prefrontal cortex assessing the effects of amyloidosis in amyloid precursor protein 23 transgenic mice	2003	Neuroscience 2003 Vol. 120 Issue 3 Pages 705-20	x							x				x		x			x		x	
M. Rolland, R. Powell, M. Jacquier-Sarlin, S. Boisseau, R. Reynaud-Dulaurier, J. Martinez-Hernandez, et al.	Effect of Aβ Oligomers on Neuronal APP Triggers a Vicious Cycle Leading to the Propagation of Synaptic Plasticity Alterations to Healthy Neurons	2020	J Neurosci 2020 Vol. 40 Issue 27 Pages 5161-5176	x							x		x		x		x	x		x		x	x
M. Ruiter, L. J. Herstel and C. J. Wierenga	Reduction of Dendritic Inhibition in CA1 Pyramidal Neurons in Amyloidosis Models of Early Alzheimer's Disease	2020	J Alzheimers Dis 2020 Vol. 78 Issue 3 Pages 951-964	x							x		x		x	x	x	x			x	x	
C. L. Russell, S. Semerdjieva, R. M. Empson, B. M. Austen, P. W. Beesley and P. Alifragis	Amyloid-β acts as a regulator of neurotransmitter release disrupting the interaction between synaptophysin and VAMP2	2012	PLoS One 2012 Vol. 7 Issue 8 Pages e43201	x								x	x		x		x	x		x			x
F. Sáez-Orellana, P. A. Godoy, C. Y. Bastidas, T. Silva-Grecchi, L. Guzmán, L. G. Aguayo and J. Fuentealba	ATP leakage induces P2XR activation and contributes to acute synaptic excitotoxicity induced by soluble oligomers of β-amyloid peptide in hippocampal neurons	2016	Neuropharmacology 2016 Vol. 100 Pages 116-23							x			x		x	x	x	x			x		x

General information				Investigated Region(s)							Used Model				Investigated Neurotransmission		Experimental Modality			Electrophysiological Assessment		Genetic Modification(s)	Substance or Intervention Investigated
Author	Title	Year	Journal	CA1	CA2	CA3	DG	EC	Subiculum	Primary Hippocampal Cells	In Vivo/Ex Vivo Mouse	In Vivo/Ex Vivo Rat	In Vitro	Human	Excitation	Inhibition	Functional Assessment	Structural Assessment	Behavioral Experiments	Plasticity	Transmission only		
G. Saleshando and J. J. O'Connor	SB203580, the p38 mitogen-activated protein kinase inhibitor blocks the inhibitory effect of beta-amyloid on long-term potentiation in the rat hippocampus	2000	Neurosci Lett 2000 Vol. 288 Issue 2 Pages 119-22				x					x			x		x			x			
I. Sánchez-Rodríguez, S. Djebari, S. Temprano-Carazo, D. Vega-Avelaira, R. Jiménez-Herrera, G. Iborra-Lázaro, et al.	Hippocampal long-term synaptic depression and memory deficits induced in early amyloidopathy are prevented by enhancing G-protein-gated inwardly rectifying potassium channel activity	2020	J Neurochem 2020 Vol. 153 Issue 3 Pages 362-376	x							x				x		x	x	x	x			x
I. Sánchez-Rodríguez, A. Gruart, J. M. Delgado-García, L. Jiménez-Díaz and J. D. Navarro-López	Role of GirK Channels in Long-Term Potentiation of Synaptic Inhibition in an In Vivo Mouse Model of Early Amyloid-β Pathology	2019	Int J Mol Sci 2019 Vol. 20 Issue 5	x							x				x	x	x			x			x
I. Sánchez-Rodríguez, S. Temprano-Carazo, A. Nájera, S. Djebari, J. Yajeya, A. Gruart, et al.	Activation of G-protein-gated inwardly rectifying potassium (Kir3/GirK) channels rescues hippocampal functions in a mouse model of early amyloid-β pathology	2017	Sci Rep 2017 Vol. 7 Issue 1 Pages 14658	x							x				x		x		x	x			x
J. L. Sanderson, R. K. Freund, J. A. Gorski and M. L. Dell'Acqua	β-Amyloid disruption of LTP/LTD balance is mediated by AKAP150-anchored PKA and Calcineurin regulation of Ca(2+)-permeable AMPA receptors	2021	Cell Rep 2021 Vol. 37 Issue 1 Pages 109786	x							x				x		x	x		x		x	
A. G. Sandoval-Hernández, L. Buitrago, H. Moreno, G. P. Cardona-Gómez and G. Arboleda	Role of Liver X Receptor in AD Pathophysiology	2015	PLoS One 2015 Vol. 10 Issue 12 Pages e0145467	x							x				x		x	x	x	x		x	x
D. Schlenzig, R. Rönicke, H. Cynis, H. H. Ludwig, E. Scheel, K. Reymann, et al.	N-Terminal pyroglutamate formation of Aβ38 and Aβ40 enforces oligomer formation and potency to disrupt hippocampal long-term potentiation	2012	J Neurochem 2012 Vol. 121 Issue 5 Pages 774-84	x							x		x		x		x			x			
C. Schmidt, E. Lepsverdize, S. L. Chi, A. M. Das, S. V. Pizzo, A. Dityatev and M. Schachner	Amyloid precursor protein and amyloid beta-peptide bind to ATP synthase and regulate its activity at the surface of neural cells	2008	Mol Psychiatry 2008 Vol. 13 Issue 10 Pages 953-69	x							x		x		x		x	x		x		x	x
G. R. Seabrook, D. W. Smith, B. J. Bowery, A. Easter, T. Reynolds, S. M. Fitzjohn, et al.	Mechanisms contributing to the deficits in hippocampal synaptic plasticity in mice lacking amyloid precursor protein	1999	Neuropharmacology 1999 Vol. 38 Issue 3 Pages 349-59	x							x				x	x	x	x		x		x	
H. J. Seo, J. E. Park, S. M. Choi, T. Kim, S. H. Cho, K. H. Lee, et al.	Inhibitory Neural Network's Impairments at Hippocampal CA1 LTP in an Aged Transgenic Mouse Model of Alzheimer's Disease	2021	Int J Mol Sci 2021 Vol. 22 Issue 2	x							x				x	x	x		x	x		x	x
F. J. Sepúlveda, H. Fierro, E. Fernandez, C. Castillo, R. W. Peoples, C. Opazo and L. G. Aguayo	Nature of the neurotoxic membrane actions of amyloid-β on hippocampal neurons in Alzheimer's disease	2014	Neurobiol Aging 2014 Vol. 35 Issue 3 Pages 472-81							x			x		x		x	x			x		x
F. J. Sepúlveda, C. Opazo and L. G. Aguayo	Alzheimer beta-amyloid blocks epileptiform activity in hippocampal neurons	2009	Mol Cell Neurosci 2009 Vol. 41 Issue 4 Pages 420-8							x			x		x		x	x			x		x

General information				Investigated Region(s)							Used Model				Investigated Neurotransmission		Experimental Modality			Electrophysiological Assessment		Genetic Modification(s)	Substance or Intervention Investigated
Author	Title	Year	Journal	CA1	CA2	CA3	DG	EC	Subiculum	Primary Hippocampal Cells	In Vivo/Ex Vivo Mouse	In Vivo/Ex Vivo Rat	In Vitro	Human	Excitation	Inhibition	Functional Assessment	Structural Assessment	Behavioral Experiments	Plasticity	Transmission only		
M. Shabani, M. Haghani, P. E. Tazangi, M. Bayat, S. M. Shid Moosavi and H. Ranjbar	Netrin-1 improves the amyloid-β-mediated suppression of memory and synaptic plasticity	2017	Brain Res Bull 2017 Vol. 131 Pages 107-116	x								x			x		x		x	x			x
S. Shahidi, S. S. Asl, A. Komaki and N. Hashemi-Firouzi	The effect of chronic stimulation of serotonin receptor type 7 on recognition, passive avoidance memory, hippocampal long-term potentiation, and neuronal apoptosis in the amyloid β protein treated rat	2018	Psychopharmacology (Berl) 2018 Vol. 235 Issue 5 Pages 1513-1525				x					x			x		x		x	x			x
S. Shahidi, N. Hashemi-Firouzi, S. S. Asl and A. Komaki	Serotonin type 6 receptor antagonist attenuates the impairment of long-term potentiation and memory induced by Abeta	2019	Behav Brain Res 2019 Vol. 364 Pages 205-212				x					x			x		x		x	x			x
S. Shahidi, S. Zargooshnia, S. S. Asl, A. Komaki and A. Sarihi	Influence of N-acetyl cysteine on beta-amyloid-induced Alzheimer's disease in a rat model: A behavioral and electrophysiological study	2017	Brain Res Bull 2017 Vol. 131 Pages 142-149				x					x			x		x		x	x			x
G. M. Shankar, B. L. Bloodgood, M. Townsend, D. M. Walsh, D. J. Selkoe and B. L. Sabatini	Natural oligomers of the Alzheimer amyloid-beta protein induce reversible synapse loss by modulating an NMDA-type glutamate receptor-dependent signaling pathway	2007	J Neurosci 2007 Vol. 27 Issue 11 Pages 2866-75	x									x		x		x	x			x		x
Y. Shen, M. Tian, Y. Zheng, F. Gong, A. K. Y. Fu and N. Y. Ip	Stimulation of the Hippocampal POMC/MC4R Circuit Alleviates Synaptic Plasticity Impairment in an Alzheimer's Disease Model	2016	Cell Rep 2016 Vol. 17 Issue 7 Pages 1819-1831	x		x					x		x		x		x	x		x		x	x
B. Shu, X. Zhang, G. Du, Q. Fu and L. Huang	MicroRNA-107 prevents amyloid-β-induced neurotoxicity and memory impairment in mice	2018	Int J Mol Med 2018 Vol. 41 Issue 3 Pages 1665-1672	x							x				x		x		x	x			x
J. P. Smith, V. Lal, D. Bowser, R. Cappai, C. L. Masters and G. D. Ciccotosto	Stimulus pattern dependence of the Alzheimer's disease amyloid-beta 42 peptide's inhibition of long term potentiation in mouse hippocampal slices	2009	Brain Res 2009 Vol. 1269 Pages 176-84	x							x				x		x			x			
W. M. Snow, K. Oikawa, J. Djordjevic and B. C. Albensi	Strain differences in hippocampal synaptic dysfunction in the TgCRND8 mouse model of Alzheimer's disease: Implications for improving translational capacity	2019	Mol Cell Neurosci 2019 Vol. 94 Pages 11-22	x							x				x		x			x		x	
A. Söderman, J. D. Mikkelsen, M. J. West, D. Z. Christensen and M. S. Jensen	Activation of nicotinic α(7) acetylcholine receptor enhances long term potentation in wild type mice but not in APP(swe)/PS1ΔE9 mice	2011	Neurosci Lett 2011 Vol. 487 Issue 3 Pages 325-9	x							x				x		x			x		x	x
P. Sompol, J. L. Furman, M. M. Pleiss, S. D. Kraner, I. A. Artiushin, S. R. Batten, et al.	Calcineurin/NFAT Signaling in Activated Astrocytes Drives Network Hyperexcitability in Aβ-Bearing Mice	2017	J Neurosci 2017 Vol. 37 Issue 25 Pages 6132-6148	x							x				x		x	x			x	x	
Y. Song, M. Hu, J. Zhang, Z. Q. Teng and C. Chen	A novel mechanism of synaptic and cognitive impairments mediated via microRNA-30b in Alzheimer's disease	2019	EBioMedicine 2019 Vol. 39 Pages 409-421	x			x				x		x	x	x		x	x	x	x		x	

General information				Investigated Region(s)							Used Model				Investigated Neurotransmission		Experimental Modality			Electrophysiological Assessment		Genetic Modification(s)	Substance or Intervention Investigated
Author	Title	Year	Journal	CA1	CA2	CA3	DG	EC	Subiculum	Primary Hippocampal Cells	In Vivo/Ex Vivo Mouse	In Vivo/Ex Vivo Rat	In Vitro	Human	Excitation	Inhibition	Functional Assessment	Structural Assessment	Behavioral Experiments	Plasticity	Transmission only		
L. Sosulina, M. Mittag, H. R. Geis, K. Hoffmann, I. Klyubin, Y. Qi, et al.	Hippocampal hyperactivity in a rat model of Alzheimer's disease	2021	J Neurochem 2021 Vol. 157 Issue 6 Pages 2128-2144	x								x			x	x	x				x	x	
J. P. Spencer, J. T. Brown, J. C. Richardson, A. D. Medhurst, S. S. Sehmi, A. R. Calver and A. D. Randall	Modulation of hippocampal excitability by 5-HT4 receptor agonists persists in a transgenic model of Alzheimer's disease	2004	Neuroscience 2004 Vol. 129 Issue 1 Pages 49-54	x							x	x			x		x				x	x	x
M. Srivareerat, T. T. Tran, K. H. Alzoubi and K. A. Alkadhi	Chronic psychosocial stress exacerbates impairment of cognition and long-term potentiation in beta-amyloid rat model of Alzheimer's disease	2009	Biol Psychiatry 2009 Vol. 65 Issue 11 Pages 918-26	x								x			x		x		x	x			
M. Srivareerat, T. T. Tran, S. Salim, A. M. Aleisa and K. A. Alkadhi	Chronic nicotine restores normal Aβ levels and prevents short-term memory and E-LTP impairment in Aβ rat model of Alzheimer's disease	2011	Neurobiol Aging 2011 Vol. 32 Issue 5 Pages 834-44	x								x			x		x		x	x			x
I. C. Stancu, L. Ris, B. Vasconcelos, C. Marinangeli, L. Goeminne, V. Laporte, et al.	Tauopathy contributes to synaptic and cognitive deficits in a murine model for Alzheimer's disease	2014	Faseb j 2014 Vol. 28 Issue 6 Pages 2620-31	x							x				x		x				x	x	
A. Stéphan, S. Laroche and S. Davis	Generation of aggregated beta-amyloid in the rat hippocampus impairs synaptic transmission and plasticity and causes memory deficits	2001	J Neurosci 2001 Vol. 21 Issue 15 Pages 5703-14				x					x			x		x			x			
V. Steubler, S. Erdinger, M. K. Back, S. Ludewig, D. Fässler, M. Richter, et al.	Loss of all three APP family members during development impairs synaptic function and plasticity, disrupts learning, and causes an autism-like phenotype	2021	Embo j 2021 Vol. 40 Issue 12 Pages e107471	x							x				x	x	x	x	x	x		x	
E. C. Suh, Y. J. Jung, Y. A. Kim, E. M. Park and K. E. Lee	A beta 25-35 induces presynaptic changes in organotypic hippocampal slice cultures	2008	Neurotoxicology 2008 Vol. 29 Issue 4 Pages 691-9	x		x	x						x		x			x			x		
E. C. Suh, Y. J. Jung, Y. A. Kim, E. M. Park, S. J. Lee and K. E. Lee	Knockout of Toll-like receptor 2 attenuates Aβ25-35-induced neurotoxicity in organotypic hippocampal slice cultures	2013	Neurochem Int 2013 Vol. 63 Issue 8 Pages 818-25			x							x		x			x			x	x	
B. Sun, B. Halabisky, Y. Zhou, J. J. Palop, G. Yu, L. Mucke and L. Gan	Imbalance between GABAergic and Glutamatergic Transmission Impairs Adult Neurogenesis in an Animal Model of Alzheimer's Disease	2009	Cell Stem Cell 2009 Vol. 5 Issue 6 Pages 624-33				x				x				x	x	x	x		x		x	x
M. K. Sun and D. L. Alkon	Impairment of hippocampal CA1 heterosynaptic transformation and spatial memory by beta-amyloid(25-35)	2002	J Neurophysiol 2002 Vol. 87 Issue 5 Pages 2441-9	x								x			x	x	x		x	x			
E. M. Szatmari, A. F. Oliveira, E. J. Sumner and R. Yasuda	Centaurin-α1-Ras-Elk-1 signaling at mitochondria mediates β-amyloid-induced synaptic dysfunction	2013	J Neurosci 2013 Vol. 33 Issue 12 Pages 5367-74	x							x		x		x		x	x			x	x	
S. Tabassum, A. Misrani, B. L. Tang, J. Chen, L. Yang and C. Long	Jujuboside A prevents sleep loss-induced disturbance of hippocampal neuronal excitability and memory impairment in young APP/PS1 mice	2019	Sci Rep 2019 Vol. 9 Issue 1 Pages 4512	x							x				x	x	x		x		x	x	x

General information				Investigated Region(s)							Used Model				Investigated Neurotransmission		Experimental Modality			Electrophysiological Assessment		Genetic Modification(s)	Substance or Intervention Investigated
Author	Title	Year	Journal	CA1	CA2	CA3	DG	EC	Subiculum	Primary Hippocampal Cells	In Vivo/Ex Vivo Mouse	In Vivo/Ex Vivo Rat	In Vitro	Human	Excitation	Inhibition	Functional Assessment	Structural Assessment	Behavioral Experiments	Plasticity	Transmission only		
R. H. Takahashi, E. Capetillo-Zarate, M. T. Lin, T. A. Milner and G. K. Gouras	Accumulation of intraneuronal β-amyloid 42 peptides is associated with early changes in microtubule-associated protein 2 in neurites and synapses	2013	PLoS One 2013 Vol. 8 Issue 1 Pages e51965	x							x				x			x			x	x	
H. Tamano, K. Ide, P. A. Adlard, A. I. Bush and A. Takeda	Involvement of hippocampal excitability in amyloid β-induced behavioral and psychological symptoms of dementia	2016	J Toxicol Sci 2016 Vol. 41 Issue 4 Pages 449-57	x		x					x				x				x		x		
A. Tamburri, A. Dudilot, S. Licea, C. Bourgeois and J. Boehm	NMDA-receptor activation but not ion flux is required for amyloid-beta induced synaptic depression	2013	PLoS One 2013 Vol. 8 Issue 6 Pages e65350	x									x		x		x				x		
D. Tampellini, N. Rahman, E. F. Gallo, Z. Huang, M. Dumont, E. Capetillo-Zarate, et al.	Synaptic activity reduces intraneuronal Abeta, promotes APP transport to synapses, and protects against Abeta-related synaptic alterations	2009	J Neurosci 2009 Vol. 29 Issue 31 Pages 9704-13	x							x		x		x		x	x			x	x	
V. T. Y. Tan, B. G. Mockett, S. M. Ohline, K. D. Parfitt, H. E. Wicky, K. Peppercorn, et al.	Lentivirus-mediated expression of human secreted amyloid precursor protein-alpha prevents development of memory and plasticity deficits in a mouse model of Alzheimer's disease	2018	Mol Brain 2018 Vol. 11 Issue 1 Pages 7	x							x		x		x		x		x	x		x	
C. J. Taylor, D. R. Ireland, I. Ballagh, K. Bourne, N. M. Marechal, P. R. Turner, et al.	Endogenous secreted amyloid precursor protein-alpha regulates hippocampal NMDA receptor function, long-term potentiation and spatial memory	2008	Neurobiol Dis 2008 Vol. 31 Issue 2 Pages 250-60				x					x			x		x		x	x			x
H. B. C. Taylor, N. J. Emptage and A. F. Jeans	Long-term depression links amyloid-β to the pathological hyperphosphorylation of tau	2021	Cell Rep 2021 Vol. 36 Issue 9 Pages 109638	x							x		x		x		x	x		x			
J. T. Ting, B. G. Kelley, T. J. Lambert, D. G. Cook and J. M. Sullivan	Amyloid precursor protein overexpression depresses excitatory transmission through both presynaptic and postsynaptic mechanisms	2007	Proc Natl Acad Sci U S A 2007 Vol. 104 Issue 1 Pages 353-8							x			x		x		x	x			x		
T. T. Tran, M. Srivareerat, I. A. Alhaider and K. A. Alkadhi	Chronic psychosocial stress enhances long-term depression in a subthreshold amyloid-beta rat model of Alzheimer's disease	2011	J Neurochem 2011 Vol. 119 Issue 2 Pages 408-16	x								x			x		x			x			x
E. Trillaud-Doppia and J. Boehm	The Amyloid Precursor Protein Intracellular Domain Is an Effector Molecule of Metaplasticity	2018	Biol Psychiatry 2018 Vol. 83 Issue 5 Pages 406-415	x									x		x		x			x		x	
F. Trinchese, S. Liu, I. Ninan, D. Puzzo, J. P. Jacob and O. Arancio	Cell cultures from animal models of Alzheimer's disease as a tool for faster screening and testing of drug efficacy	2004	J Mol Neurosci 2004 Vol. 24 Issue 1 Pages 15-21							x			x		x		x	x			x	x	
M. R. Tropea, D. D. Li Puma, M. Melone, W. Gulisano, O. Arancio, C. Grassi, et al.	Genetic deletion of α7 nicotinic acetylcholine receptors induces an age-dependent Alzheimer's disease-like pathology	2021	Prog Neurobiol 2021 Vol. 206 Pages 102154	x							x				x		x		x	x		x	

General information				Investigated Region(s)							Used Model				Investigated Neurotransmission		Experimental Modality			Electrophysiological Assessment		Genetic Modification(s)	Substance or Intervention Investigated
Author	Title	Year	Journal	CA1	CA2	CA3	DG	EC	Subiculum	Primary Hippocampal Cells	In Vivo/Ex Vivo Mouse	In Vivo/Ex Vivo Rat	In Vitro	Human	Excitation	Inhibition	Functional Assessment	Structural Assessment	Behavioral Experiments	Plasticity	Transmission only		
L. Trujillo-Estrada, J. C. Dávila, E. Sánchez-Mejias, R. Sánchez-Varo, A. Gomez-Arboledas, M. Vizuete, et al.	Early neuronal loss and axonal/presynaptic damage is associated with accelerated amyloid-β accumulation in AβPP/PS1 Alzheimer's disease mice subiculum	2014	J Alzheimers Dis 2014 Vol. 42 Issue 2 Pages 521-41						x		x				x	x		x			x	x	
S. T. Tsai, S. Y. Chen, S. Z. Lin and G. F. Tseng	Rostral intralaminar thalamic deep brain stimulation ameliorates memory deficits and dendritic regression in β-amyloid-infused rats	2020	Brain Struct Funct 2020 Vol. 225 Issue 2 Pages 751-761							x		x			x		x	x	x	x			x
M. van den Berg, M. H. Adhikari, M. Verschuuren, I. Pintelon, T. Vasilkovska, J. Van Audekerke, et al.	Altered basal forebrain function during whole-brain network activity at pre- and early-plaque stages of Alzheimer's disease in TgF344-AD rats	2022	Alzheimers Res Ther 2022 Vol. 14 Issue 1 Pages 148					x		x		x			x	x	x	x				x	
E. Varga, G. Juhász, Z. Bozsó, B. Penke, L. Fülöp and V. Szegedi	Abeta(1-42) enhances neuronal excitability in the CA1 via NR2B subunit-containing NMDA receptors	2014	Neural Plast 2014 Vol. 2014 Pages 584314	x							x				x		x			x			
J. Y. Vargas, J. Ahumada, M. S. Arrázola, M. Fuenzalida and N. C. Inestrosa	WASP-1, a canonical Wnt signaling potentiator, rescues hippocampal synaptic impairments induced by Aβ oligomers	2015	Exp Neurol 2015 Vol. 264 Pages 14-25	x							x		x		x		x			x		x	x
A. Volianskis, R. Køstner, M. Mølgaard, S. Hass and M. S. Jensen	Episodic memory deficits are not related to altered glutamatergic synaptic transmission and plasticity in the CA1 hippocampus of the APPswe/PS1δE9-deleted transgenic mice model of ß-amyloidosis	2010	Neurobiol Aging 2010 Vol. 31 Issue 7 Pages 1173-87	x							x				x		x	x	x	x		x	
B. Wang, Z. Wang, L. Sun, L. Yang, H. Li, A. L. Cole, et al.	The amyloid precursor protein controls adult hippocampal neurogenesis through GABAergic interneurons	2014	J Neurosci 2014 Vol. 34 Issue 40 Pages 13314-25				x				x		x		x	x	x	x	x		x	x	x
H. W. Wang, J. F. Pasternak, H. Kuo, H. Ristic, M. P. Lambert, B. Chromy, et al.	Soluble oligomers of beta amyloid (1-42) inhibit long-term potentiation but not long-term depression in rat dentate gyrus	2002	Brain Res 2002 Vol. 924 Issue 2 Pages 133-40				x					x			x		x				x		
J. Wang, Y. He, X. Chen, L. Huang, J. Li, Z. You, et al.	Metabotropic glutamate receptor 5 (mGluR5) is associated with neurodegeneration and amyloid deposition in Alzheimer's disease: A [(18)F]PSS232 PET/MRI study	2024	Alzheimers Res Ther 2024 Vol. 16 Issue 1 Pages 9							x				x	x			x					
K. Wang, W. Sun, L. Zhang, W. Guo, J. Xu, S. Liu, et al.	Oleanolic Acid Ameliorates Aβ25-35 Injection-induced Memory Deficit in Alzheimer's Disease Model Rats by Maintaining Synaptic Plasticity	2018	CNS Neurol Disord Drug Targets 2018 Vol. 17 Issue 5 Pages 389-399	x								x			x		x	x		x			x
L. Wang, H. Kang, Y. Li, Y. Shui, R. Yamamoto, T. Sugai and N. Kato	Cognitive recovery by chronic activation of the large-conductance calcium-activated potassium channel in a mouse model of Alzheimer's disease	2015	Neuropharmacology 2015 Vol. 92 Pages 8-15	x							x				x		x		x	x		x	x
Q. Wang, I. Klyubin, S. Wright, I. Griswold-Prenner, M. J. Rowan and R. Anwyl	Alpha v integrins mediate beta-amyloid induced inhibition of long-term potentiation	2008	Neurobiol Aging 2008 Vol. 29 Issue 10 Pages 1485-93	x			x					x			x		x			x			

General information				Investigated Region(s)							Used Model				Investigated Neurotransmission		Experimental Modality			Electrophysiological Assessment		Genetic Modification(s)	Substance or Intervention Investigated
Author	Title	Year	Journal	CA1	CA2	CA3	DG	EC	Subiculum	Primary Hippocampal Cells	In Vivo/Ex Vivo Mouse	In Vivo/Ex Vivo Rat	In Vitro	Human	Excitation	Inhibition	Functional Assessment	Structural Assessment	Behavioral Experiments	Plasticity	Transmission only		
Q. Wang, D. M. Walsh, M. J. Rowan, D. J. Selkoe and R. Anwyl	Block of long-term potentiation by naturally secreted and synthetic amyloid beta-peptide in hippocampal slices is mediated via activation of the kinases c-Jun N-terminal kinase, cyclin-dependent kinase 5, and p38 mitogen-activated protein kinase as well as metabotropic glutamate receptor type 5	2004	J Neurosci 2004 Vol. 24 Issue 13 Pages 3370-8				x					x			x		x			x			x
S. Wang, J. Huang, Y. Chen, Y. Liang, L. Chen, D. Ye, et al.	Qifu-yin activates the Keap1/Nrf2/ARE signaling and ameliorates synaptic injury and oxidative stress in APP/PS1 mice	2024	J Ethnopharmacol 2024 Vol. 333 Pages 118497	x							x				x			x	x		x	x	x
X. H. Wang, L. Li, C. Hölscher, Y. F. Pan, X. R. Chen and J. S. Qi	Val8-glucagon-like peptide-1 protects against Aβ1-40-induced impairment of hippocampal late-phase long-term potentiation and spatial learning in rats	2010	Neuroscience 2010 Vol. 170 Issue 4 Pages 1239-48	x								x			x		x		x	x			x
X. L. Wang, Y. X. Deng, Y. M. Gao, Y. T. Dong, F. Wang, Z. Z. Guan, et al.	Activation of α7 nAChR by PNU-282987 improves synaptic and cognitive functions through restoring the expression of synaptic-associated proteins and the CaM-CaMKII-CREB signaling pathway	2020	Aging (Albany NY) 2020 Vol. 12 Issue 1 Pages 543-570	x	x	x	x				x		x		x			x	x		x	x	
Y. Wang, N. H. Greig, Q. S. Yu and M. P. Mattson	Presenilin-1 mutation impairs cholinergic modulation of synaptic plasticity and suppresses NMDA currents in hippocampus slices	2009	Neurobiol Aging 2009 Vol. 30 Issue 7 Pages 1061-8	x							x				x		x			x		x	
Y. Wang, L. Wu, J. Li, D. Fang, C. Zhong, J. X. Chen and S. S. Yan	Synergistic exacerbation of mitochondrial and synaptic dysfunction and resultant learning and memory deficit in a mouse model of diabetic Alzheimer's disease	2015	J Alzheimers Dis 2015 Vol. 43 Issue 2 Pages 451-63	x							x				x		x		x	x		x	x
Z. Wang, B. Wang, L. Yang, Q. Guo, N. Aithmitti, Z. Songyang and H. Zheng	Presynaptic and postsynaptic interaction of the amyloid precursor protein promotes peripheral and central synaptogenesis	2009	J Neurosci 2009 Vol. 29 Issue 35 Pages 10788-801							x			x		x			x			x		
W. Wei, L. N. Nguyen, H. W. Kessels, H. Hagiwara, S. Sisodia and R. Malinow	Amyloid beta from axons and dendrites reduces local spine number and plasticity	2010	Nat Neurosci 2010 Vol. 13 Issue 2 Pages 190-6	x									x		x			x		x		x	
P. J. Welsby, M. J. Rowan and R. Anwyl	Beta-amyloid blocks high frequency stimulation induced LTP but not nicotine enhanced LTP	2007	Neuropharmacology 2007 Vol. 53 Issue 1 Pages 188-95				x				x	x			x		x			x			x
D. J. Whitcomb, E. L. Hogg, P. Regan, T. Piers, P. Narayan, G. Whitehead, et al.	Intracellular oligomeric amyloid-beta rapidly regulates GluA1 subunit of AMPA receptor in the hippocampus	2015	Sci Rep 2015 Vol. 5 Pages 10934	x								x	x		x		x				x		
J. Witton, J. T. Brown, M. W. Jones and A. D. Randall	Altered synaptic plasticity in the mossy fibre pathway of transgenic mice expressing mutant amyloid precursor protein	2010	Mol Brain 2010 Vol. 3 Pages 32			x					x				x		x			x		x	

General information				Investigated Region(s)							Used Model				Investigated Neurotransmission		Experimental Modality			Electrophysiological Assessment		Genetic Modification(s)	Substance or Intervention Investigated
Author	Title	Year	Journal	CA1	CA2	CA3	DG	EC	Subiculum	Primary Hippocampal Cells	In Vivo/Ex Vivo Mouse	In Vivo/Ex Vivo Rat	In Vitro	Human	Excitation	Inhibition	Functional Assessment	Structural Assessment	Behavioral Experiments	Plasticity	Transmission only		
G. M. Wu and X. Y. Hou	Oligomerized Abeta25-35 induces increased tyrosine phosphorylation of NMDA receptor subunit 2A in rat hippocampal CA1 subfield	2010	Brain Res 2010 Vol. 1343 Pages 186-93	x								x			x			x			x		x
J. Wu, R. Anwyl and M. J. Rowan	beta-Amyloid-(1-40) increases long-term potentiation in rat hippocampus in vitro	1995	Eur J Pharmacol 1995 Vol. 284 Issue 3 Pages R1-3				x						x		x		x			x			
M. N. Wu, Y. X. He, F. Guo and J. S. Qi	Alpha4beta2 nicotinic acetylcholine receptors are required for the amyloid beta protein-induced suppression of long-term potentiation in rat hippocampal CA1 region in vivo	2008	Brain Res Bull 2008 Vol. 77 Issue 2-3 Pages 84-90	x								x			x		x			x			x
R. Wykes, A. Kalmbach, M. Eliava and J. Waters	Changes in the physiology of CA1 hippocampal pyramidal neurons in preplaque CRND8 mice	2012	Neurobiol Aging 2012 Vol. 33 Issue 8 Pages 1609-23	x							x				x		x	x			x	x	
E. Xia, F. Xu, C. Hu, J. P. P. Kumal, X. Tang, D. Mao, et al.	Young Blood Rescues the Cognition of Alzheimer's Model Mice by Restoring the Hippocampal Cholinergic Circuit	2019	Neuroscience 2019 Vol. 417 Pages 57-69	x							x				x			x	x		x	x	x
C. Xing, Y. Yin, R. Chang, X. He and Z. Xie	A role of insulin-like growth factor 1 in beta amyloid-induced disinhibition of hippocampal neurons	2005	Neurosci Lett 2005 Vol. 384 Issue 1-2 Pages 93-7							x			x			x	x	x			x		x
T. Yang, J. K. Knowles, Q. Lu, H. Zhang, O. Arancio, L. A. Moore, et al.	Small molecule, non-peptide p75 ligands inhibit Abeta-induced neurodegeneration and synaptic impairment	2008	PLoS One 2008 Vol. 3 Issue 11 Pages e3604							x			x		x		x	x		x			x
Y. Yang, W. G. Ji, Y. J. Zhang, L. P. Zhou, H. Chen, N. Yang and Z. R. Zhu	Riluzole ameliorates soluble Aβ(1-42)-induced impairments in spatial memory by modulating the glutamatergic/GABAergic balance in the dentate gyrus	2021	Prog Neuropsychopharmacol Biol Psychiatry 2021 Vol. 108 Pages 110077				x					x			x	x	x		x	x			x
Y. Yang, W. G. Ji, Z. R. Zhu, Y. L. Wu, Z. Y. Zhang and S. C. Qu	Rhynchophylline suppresses soluble Aβ(1-42)-induced impairment of spatial cognition function via inhibiting excessive activation of extrasynaptic NR2B-containing NMDA receptors	2018	Neuropharmacology 2018 Vol. 135 Pages 100-112				x					x			x		x	x	x	x			
W. Yao, H. J. Zou, D. Sun and S. Q. Ren	Aβ induces acute depression of excitatory glutamatergic synaptic transmission through distinct phosphatase-dependent mechanisms in rat CA1 pyramidal neurons	2013	Brain Res 2013 Vol. 1515 Pages 88-97	x								x			x		x				x		x
H. Ye, S. Jalini, S. Mylvaganam and P. Carlen	Activation of large-conductance Ca(2+)-activated K(+) channels depresses basal synaptic transmission in the hippocampal CA1 area in APP (swe/ind) TgCRND8 mice	2010	Neurobiol Aging 2010 Vol. 31 Issue 4 Pages 591-604	x							x				x		x				x	x	x
Q. Yu, H. Liu, S. Sang, L. Chen, Y. Zhao, Y. Wang and C. Zhong	Thiamine deficiency contributes to synapse and neural circuit defects	2018	Biol Res 2018 Vol. 51 Issue 1 Pages 35							x			x		x			x			x		x

General information				Investigated Region(s)							Used Model				Investigated Neurotransmission		Experimental Modality			Electrophysiological Assessment		Genetic Modification(s)	Substance or Intervention Investigated
Author	Title	Year	Journal	CA1	CA2	CA3	DG	EC	Subiculum	Primary Hippocampal Cells	In Vivo/Ex Vivo Mouse	In Vivo/Ex Vivo Rat	In Vitro	Human	Excitation	Inhibition	Functional Assessment	Structural Assessment	Behavioral Experiments	Plasticity	Transmission only		
Q. Yu, Y. Wang, F. Du, S. Yan, G. Hu, N. Origlia, et al.	Overexpression of endophilin A1 exacerbates synaptic alterations in a mouse model of Alzheimer's disease	2018	Nat Commun 2018 Vol. 9 Issue 1 Pages 2968	x							x		x		x		x	x	x	x		x	x
D. Zare, M. A. Rajizadeh, M. Maneshian, H. Jonaidi, V. Sheibani, M. Asadi-Shekaari, et al.	Inhibition of protease-activated receptor 1 (PAR1) ameliorates cognitive performance and synaptic plasticity impairments in animal model of Alzheimer's diseases	2021	Psychopharmacology (Berl) 2021 Vol. 238 Issue 6 Pages 1645-1656	x								x			x		x		x	x		x	x
D. Zhang, A. J. Mably, D. M. Walsh and M. J. Rowan	Peripheral Interventions Enhancing Brain Glutamate Homeostasis Relieve Amyloid β- and TNFα- Mediated Synaptic Plasticity Disruption in the Rat Hippocampus	2017	Cereb Cortex 2017 Vol. 27 Issue 7 Pages 3724-3735	x								x			x		x			x			x
J. Zhang, M. Hu, Z. Teng, Y. P. Tang and C. Chen	Synaptic and cognitive improvements by inhibition of 2-AG metabolism are through upregulation of microRNA-188-3p in a mouse model of Alzheimer's disease	2014	J Neurosci 2014 Vol. 34 Issue 45 Pages 14919-33				x				x				x		x	x		x		x	
J. Zhang, C. Wang, T. Deng, Z. Xue, X. Chen, L. Chang and Q. Wang	The preventive effect of NR2B and NR2D-containing NMDAR antagonists on Aβ-induced LTP disruption in the dentate gyrus of rats	2013	Metab Brain Dis 2013 Vol. 28 Issue 4 Pages 697-704				x					x			x		x			x			x
J. F. Zhang, J. S. Qi and J. T. Qiao	Protein kinase C mediates amyloid beta-protein fragment 31-35-induced suppression of hippocampal late-phase long-term potentiation in vivo	2009	Neurobiol Learn Mem 2009 Vol. 91 Issue 3 Pages 226-34	x								x			x		x			x			x
W. Zhang, J. Miao, J. Hao, Z. Li, J. Xu, R. Liu, et al.	Protective effect of S14G-humanin against beta-amyloid induced LTP inhibition in mouse hippocampal slices	2009	Peptides 2009 Vol. 30 Issue 6 Pages 1197-202	x							x				x		x			x			x
X. Zhang, U. Herrmann, S. W. Weyer, M. Both, U. C. Müller, M. Korte and A. Draguhn	Hippocampal network oscillations in APP/APLP2-deficient mice	2013	PLoS One 2013 Vol. 8 Issue 4 Pages e61198	x		x					x				x		x			x		x	
X. Zhang, Y. Mei, Y. He, D. Wang, J. Wang, X. Wei, et al.	Ablating Adult Neural Stem Cells Improves Synaptic and Cognitive Functions in Alzheimer Models	2021	Stem Cell Reports 2021 Vol. 16 Issue 1 Pages 89-105				x				x				x	x	x		x	x		x	x
Z. Zhang, R. Chen, W. An, C. Wang, G. Liao, X. Dong, et al.	A novel acetylcholinesterase inhibitor and calcium channel blocker SCR-1693 improves Aβ25-35-impaired mouse cognitive function	2016	Psychopharmacology (Berl) 2016 Vol. 233 Issue 4 Pages 599-613	x							x		x		x		x		x	x			x
G. Zhao, H. L. Liu, H. Zhang and X. J. Tong	Treadmill exercise enhances synaptic plasticity, but does not alter β-amyloid deposition in hippocampi of aged APP/PS1 transgenic mice	2015	Neuroscience 2015 Vol. 298 Pages 357-66				x				x				x				x	x		x	
W. H. Zhi, Y. Y. Zeng, Z. H. Lu, W. J. Qu, W. X. Chen, L. Chen and L. Chen	Simvastatin exerts antiamnesic effect in Aβ25-35 -injected mice	2014	CNS Neurosci Ther 2014 Vol. 20 Issue 3 Pages 218-26	x							x				x		x		x	x			x

General information				Investigated Region(s)							Used Model				Investigated Neurotransmission		Experimental Modality			Electrophysiological Assessment		Genetic Modification(s)	Substance or Intervention Investigated
**Author**	**Title**	**Year**	**Journal**	**CA1**	**CA2**	**CA3**	**DG**	**EC**	**Subiculum**	**Primary Hippocampal Cells**	**In Vivo/Ex Vivo Mouse**	**In Vivo/Ex Vivo Rat**	**In Vitro**	**Human**	**Excitation**	**Inhibition**	**Functional Assessment**	**Structural Assessment**	**Behavioral Experiments**	**Plasticity**	**Transmission only**		
R. Zhou and P. Bickler	Interaction of Isoflurane, Tumor Necrosis Factor-α and β-Amyloid on Long-term Potentiation in Rat Hippocampal Slices	2017	Anesth Analg 2017 Vol. 124 Issue 2 Pages 582-587	x								x			x		x			x			x
Y. Zhou, D. Luo, J. Shi, X. Yang, W. Xu, W. Gao, et al.	Loganin alleviated cognitive impairment in 3×Tg-AD mice through promoting mitophagy mediated by optineurin	2023	J Ethnopharmacol 2023 Vol. 312 Pages 116455							x	x				x			x		x		x	x
H. Zhu, H. Yan, N. Tang, X. Li, P. Pang, H. Li, et al.	Impairments of spatial memory in an Alzheimer's disease model via degeneration of hippocampal cholinergic synapses	2017	Nat Commun 2017 Vol. 8 Issue 1 Pages 1676				x				x				x		x		x	x		x	
