# Supplementary material for: A synapse perspective on the function of the amyloid precursor protein
Source: Sci Prog. 2025 Jul 30;108(3):00368504251360728. doi: 10.1177/00368504251360728 (PMC12317227; doi:10.1177/00368504251360728)
Supplement: sj-pdf-2-sci-10.1177_00368504251360728 - Supplemental material for A synapse perspective on the function of the amyloid precursor protein [file sj-pdf-2-sci-10.1177_00368504251360728.pdf]

Literature Research for CA1

General Information				Used Model						Genetic Modification		Investigated Neurotransmission		Signalling Pathway	Functional Analysis			Structural Analysis		Behavioral Experiments	Plasticity			Investigated APP fragments and domains	Investigated Substance, Intervention or Condition
Authors	Title	Year	Journal	In Vivo Rat	In Vivo Mouse	Ex Vivo Rat	Ex Vivo Mouse	In Vitro	Human	Transgen Line	APP-Related Viral Modifications	Excitiation	Inhibition		Field Potential Recordings	Whole Cell Recordings	Imaging	Light Microscopy	Electron Microscopy		LTP	LTD	STP		
E. Abramov, I. Dolev, H. Fogel, G. D. Ciccotosto, E. Ruff and I. Slutsky	Amyloid-β as a positive endogenous regulator of release probability at hippocampal synapses	2009	Nature Neuroscience	x				Primary Cells		APP-KO		x	x		x	x	x						x		Thiorphan
S. Alfonso, H. W. Kessels, C. C. Banos, T. R. Chan, E. T. Lin, G. Kumaravel, et al.	Synapto-depressive effects of amyloid β require PICK1	2014	Eur J Neurosci		x		x	OTC, Primary Cells		PICK1-KO	APP-CT100	x		PICK1		x		x						Aβ1-42	BIO922
S. Alves, G. Churlaud, M. Audrain, K. Michaelsen-Preusse, R. Fol, B. Souchet, et al.	Interleukin-2 improves amyloid pathology, synaptic failure and memory in Alzheimer's disease mice	2017	Brain		x				x	APPswe/PS1ΔE9		x		IL2, JAK/STAT3	x			x		x	x				
A. Androuin, B. Potier, U. V. Nägerl, D. Cattaert, L. Danglot, M. Thierry, et al.	Evidence for altered dendritic spine compartmentalization in Alzheimer's disease and functional effects in a mouse model	2018	Acta Neuropathol		x		x		x	APPxPS1-KI, PS1-KI		x			x	x			x	x	x				
J. Arévalo-Serrano, J. M. Sanz-Anquela and A. Gonzalo-Ruiz	β-amyloid peptide-induced modifications in α7 nicotinic acetylcholine receptor immunoreactivity in the hippocampus of the rat: relationship with GABAergic and calcium-binding proteins perikarya	2008	Brain Res Bull			x							x					x						Aβ1-40	
A. E. Barry, I. Klyubin, J. M. Mc Donald, A. J. Mably, M. A. Farrell, M. Scott, et al.	Alzheimer's disease brain-derived amyloid-β-mediated inhibition of LTP in vivo is prevented by immunotargeting cellular prion protein	2011	J Neurosci	x								x		PrPc	x						x			human Aβ	
L. Biasetti, S. Rey, M. Fowler, A. Ratnayaka, K. Fennell, C. Smith, et al.	Elevated amyloid β disrupts the nanoscale organization and function of synaptic vesicle pools in hippocampal neurons	2023	Cereb Cortex				x	Primary Cells		APPSw,Ind		x					x		x					Aβ1-42	
J. M. Billard and T. Freret	Improved NMDA Receptor Activation by the Secreted Amyloid-Protein Precursor-α in Healthy Aging: A Role for D-Serine?	2022	Int J Mol Sci		x							x		D-serine	x						x		x	sAPPα	
Z. Bozso, B. Penke, D. Simon, I. Laczkó, G. Juhász, V. Szegedi, et al.	Controlled in situ preparation of Ab(1–42) oligomers from the isopeptide ''iso-Ab(1–42)'', physicochemical and biological characterization	2010	Peptides	x		x						x			x					x	x			Aβ1-42	
H. Breyhan, O. Wirths, K. Duan, A. Marcello, J. Rettig and T. A. Bayer	APP/PS1KI bigenic mice develop early synaptic deficits and hippocampus atrophy	2009	Acta Neuropathol				x			APP/PS1-KI		x			x						x				
M. Bürge, S. Kratzer, C. Mattusch, C. Hofmann, M. Kreuzer, C. G. Parsons and G. Rammes	The anaesthetic xenon partially restores an amyloid β-induced impairment in murine hippocampal synaptic plasticity	2019	Neuropharmacology				x					x			x						x			Aβ1-40, Aβ1-42	Xenon, S-ketamine, Radiprodil
C. Cai, L. Wang, S. Li, S. Lou, J. L. Luo, D. Y. Fu and T. Chen	Ras Inhibitor Lonafarnib Rescues Structural and Functional Impairments of Synapses of Aβ(1-42) Mice via α7nAChR-Dependent BDNF Upregulation	2022	J Neurosci		x		x					x		H-RAS	x			x		x	x		x	Aβ1-42	Lonafarnib
H. Y. Cai, C. Hölscher, X. H. Yue, S. X. Zhang, X. H. Wang, F. Qiao, et al.	Lixisenatide rescues spatial memory and synaptic plasticity from amyloid β protein-induced impairments in rats	2014	Neuroscience	x								x		GSK3b	x					x	x			Aβ25-35	Lixisenatide
B. Calvo-Flores Guzmán, S. Kim, B. Chawdhary, K. Peppercorn, W. P. Tate, H. J. Waldvogel, et al.	Amyloid-β(1-42) -Induced Increase in GABAergic Tonic Conductance in Mouse Hippocampal CA1 Pyramidal Cells	2020	Molecules		x		x						x			x		x		x				Aβ1-42	
V. Cavallucci, N. Berretta, A. Nobili, R. Nisticò, N. B. Mercuri and M. D'Amelio	Calcineurin inhibition rescues early synaptic plasticity deficits in a mouse model of Alzheimer's disease	2013	Neuromolecular Med				x			Tg2576		x			x							x			FK506
K. Ceyzériat, L. Ben Haim, A. Denizot, D. Pommier, M. Matos, O. Guillemaud, et al.	Modulation of astrocyte reactivity improves functional deficits in mouse models of Alzheimer's disease	2018	Acta Neuropathol Commun		x		x			APP/PS1ΔE9, 3xTg		x		JAK2-STAT3	x			x		x	x				
S. Chakroborty, C. Briggs, M. B. Miller, I. Goussakov, C. Schneider, J. Kim, et al.	Stabilizing ER Ca2+ channel function as an early preventative strategy for Alzheimer's disease	2012	PLoS One				x			3xTg-AD, TASTPM		x		RyR	x	x	x	x			x		x		Dantrolen
S. Chakroborty, J. Kim, C. Schneider, A. R. West and G. E. Stutzmann	Nitric oxide signaling is recruited as a compensatory mechanism for sustaining synaptic plasticity in Alzheimer's disease mice	2015	J Neurosci				x			3xTg-AD		x		RyR, NO	x		x				x	x	x		NOS-Inhibitor
L. Chen, K. Yamada, T. Nabeshima and M. Sokabe	α7 Nicotinic acetylcholine receptor as a target to rescue deficit in hippocampal LTP induction in β-amyloid infused rats	2006	Neuropharmacology	x		x						x			x		x				x			Aβ1-40, Aβ40-1	
L. Chen, Z. Zhuang, H. Duan, D. Lv, S. Hong, P. Chen, et al.	Corilagin improves cognitive impairment in APP/PS1 mice by reducing Aβ generation and enhancing synaptic plasticity	2024	Eur J Pharmacol		x					APP/PS1		x						x	x	x					Corilagin
M. Chen, J. Wang, J. Jiang, X. Zheng, N. J. Justice, K. Wang, et al.	APP modulates KCC2 expression and function in hippocampal GABAergic inhibition	2017	Elife				x	Primary Cells		APP-KO, sAPPβ-KI, hAPP695-KI, C99-KI			x	KCC, tyrosine		x		x							
Y. Chen and T. Behnisch	The role of γ-secretase in hippocampal synaptic transmission and activity-dependent synaptic plasticity	2013	Neurosci Lett			x						x			x						x				DAPT/Compound E
S. A. Chong, I. Benilova, H. Shaban, B. De Strooper, H. Devijver, D. Moechars, et al.	Synaptic dysfunction in hippocampus of transgenic mouse models of Alzheimer's disease: a multi-electrode array study	2011	Neurobiol Dis				x			APP.V717I, APP.V717I×Tau.P301L		x			x						x			Aβ1-42/Aβ1-40	
C. Colussi, G. Aceto, C. Ripoli, A. Bertozzi, D. D. Li Puma, E. Paccosi, et al.	Cytoplasmic HDAC4 recovers synaptic function in the 3×Tg mouse model of Alzheimer's disease	2023	Neuropathol Appl Neurobiol					Primary Cells, OTC		3x Tg-AD		x				x		x							
D. A. Costello and C. E. Herron	The role of c-Jun N-terminal kinase in the A β-mediated impairment of LTP and regulation of synaptic transmission in the hippocampus	2004	Neuropharmacology			x						x		JNK	x						x		x	Aβ25-35	JNK-inhibitor
D. A. Costello, D. M. O'Leary and C. E. Herron	Agonists of peroxisome proliferator-activated receptor-gamma attenuate the Aβ-mediated impairment of LTP in the hippocampus in vitro	2005	Neuropharmacology			x						x		PPARγ	x						x		x	Aβ1-40	PPARγ agonists
D. M. Cummings, W. Liu, E. Portelius, S. Bayram, M. Yasvoina, S. H. Ho, et al.	First effects of rising amyloid-β in transgenic mouse brain: synaptic transmission and gene expression	2015	Brain				x			TASTPM		x				x		x					x		

General Information				Used Model						Genetic Modification		Investigated Neurotransmission		Signalling Pathway	Functional Analysis			Structural Analysis		Behavioral Experiments	Plasticity			Investigated APP fragments and domains	Investigated Substance, Intervention or Condition
Authors	Title	Year	Journal	In Vivo Rat	In Vivo Mouse	Ex Vivo Rat	Ex Vivo Mouse	In Vitro	Human	Transgen Line	APP-Related Viral Modifications	Excitation	Inhibition		Field Potential Recordings	Whole Cell Recordings	Imaging	Light Microscopy	Electron Microscopy		LTP	LTD	STP		
D. Del Prete, F. Lombino, X. Liu and L. D'Adamio	APP is cleaved by Bace1 in pre-synaptic vesicles and establishes a pre-synaptic interactome, via its intracellular domain, with molecular complexes that regulate pre-synaptic vesicles functions	2014	PLoS One				x					x							x						
M. C. Dinamarca, M. Di Luca, J. A. Godoy and N. C. Inestrosa	The soluble extracellular fragment of neuroligin-1 targets Aβ oligomers to the postsynaptic region of excitatory synapses	2015	Biochem Biophys Res Commun			x		OTC				x			x	x		x			x			Aβ1-40, Aβ1-42	
G. H. Doherty, D. Beccano-Kelly, S. D. Yan, F. J. Gunn-Moore and J. Harvey	Leptin prevents hippocampal synaptic disruption and neuronal cell death induced by amyloid β	2013	Neurobiol Aging			x		Primary Cells				x		STAT3	x			x			x	x		Aβ1-42	Leptin
H. Dong, M. V. Martin, S. Chambers and J. G. Csernansky	Spatial relationship between synapse loss and β-amyloid deposition in Tg2576 mice	2007	J Comp Neurol				x			Tg2576		x						x	x						
J. Dunot, S. Moreno, C. Gandin, P. A. Pousinha, M. Amici, J. Dupuis, et al.	APP fragment controls both ionotropic and non-ionotropic signaling of NMDA receptors	2024	Neuron		x	x	x	Primary Cells		APPdelETA		x			x	x	x	x		x		x	x	Aη	MK801
M. S. Durakoglugil, Y. Chen, C. L. White, E. T. Kavalali and J. Herz	Reelin signaling antagonizes β-amyloid at the synapse	2009	Proc Natl Acad Sci U S A				x					x			x	x					x			Aβ1-42, Aβ25-35	Reelin
P. Dutar and B. Potier	Susceptibility to Aβo and TBOA of LTD and Extrasynaptic NMDAR-Dependent Tonic Current in the Aged Rat Hippocampus	2019	Neurochem Res			x						x			x							x		Aβ	
P. Esmaeili Tazangi, S. M. Moosavi, M. Shabani and M. Haghani	Erythropoietin improves synaptic plasticity and memory deficits by decrease of the neurotransmitter release probability in the rat model of Alzheimer's disease	2015	Pharmacol Biochem Behav	x		x						x			x					x	x		x	Aβ25-35	Erythropoetin
E. Faldini, T. Ahmed, L. Bueé, D. Blum and D. Balschun	Tau- but not Aβ -pathology enhances NMDAR-dependent depotentiation in AD-mouse models	2019	Acta Neuropathol Commun				x			APP/PS1-21		x			x						x				
T. Fanutza, D. Del Prete, M. J. Ford, P. E. Castillo and L. D'Adamio	APP and APLP2 interact with the synaptic release machinery and facilitate transmitter release at hippocampal synapses	2015	Elife				x			APP-KO, APP/APLP2-KO		x			x	x		x					x	AICD	
S. M. Fitzjohn, F. Kuenzi, R. A. Morton, T. W. Rosahl, H. Lewis, D. Smith, et al.	A study of long-term potentiation in transgenic mice over-expressing mutant forms of both amyloid precursor protein and presenilin-1	2010	Mol Brain				x			APP695swe, APP695swe x PS1A246E		x			x						x		x		
S. M. Fitzjohn, R. A. Morton, F. Kuenzi, C. H. Davies, G. R. Seabrook and G. L. Collingridge	Similar levels of long-term potentiation in amyloid precursor protein -null and wild-type mice in the CA1 region of picrotoxin treated slices	2000	Neurosci Lett				x			APP-KO		x	x		x	x					x				
S. M. Fitzjohn, R. A. Morton, F. Kuenzi, T. W. Rosahl, M. Shearman, H. Lewis, et al.	Age-related impairment of synaptic transmission but normal long-term potentiation in transgenic mice that overexpress the human APP695SWE mutant form of amyloid precursor protein	2001	J Neurosci				x			APP695swe, APP695swe x PS1A246E		x			x						x		x		
H. Fogel, S. Frere, O. Segev, S. Bharill, I. Shapira, N. Gazit, et al.	APP homodimers transduce an amyloid-β-mediated increase in release probability at excitatory synapses	2014	Cell Rep				x	Primary Cells		APP-KO		x		APP/Go-Protein Signaling Complex			x	x						Aβ1-40	
G. Fonar, B. Polis, D. S. Sams, A. Levi, A. Malka, N. Bal, et al.	Modified Snake α-Neurotoxin Averts β-Amyloid Binding to α7 Nicotinic Acetylcholine Receptor and Reverses Cognitive Deficits in Alzheimer's Disease Mice	2021	Mol Neurobiol		x		x			3 x Tg		x			x					x	x		x	Aβ25-35	Snake cobratoxin
D. B. Freir, D. A. Costello and C. E. Herron	A β 25-35-induced depression of long-term potentiation in area CA1 in vivo and in vitro is attenuated by verapamil	2003	J Neurophysiol	x								x			x						x			Aβ25-35	Verapamil, Diltiacem
D. B. Freir, C. Holscher and C. E. Herron	Blockade of long-term potentiation by β-amyloid peptides in the CA1 region of the rat hippocampus in vivo	2001	J Neurophysiol	x								x			x						x			Aβ15-25, Aβ25-35, Aβ35-25	
A. R. Fusilier, J. A. Davis, J. R. Paul, S. D. Yates, L. J. McMeekin, L. K. Goode, et al.	Dysregulated clock gene expression and abnormal diurnal regulation of hippocampal inhibitory transmission and spatial memory in amyloid precursor protein transgenic mice	2021	Neurobiol Dis		x		x			Tg-SwDI			x		x	x		x		x					
V. A. Gault and C. Hölscher	GLP-1 agonists facilitate hippocampal LTP and reverse the impairment of LTP induced by β-amyloid	2008	Eur J Pharmacol	x								x			x						x			Aβ25-35	GLP1
V. A. Gault and C. Hölscher	Protease-resistant glucose-dependent insulinotropic polypeptide agonists facilitate hippocampal LTP and reverse the impairment of LTP induced by β-amyloid	2008	J Neurophysiol	x								x			x						x			Aβ25-35	GIP, N-AcGIP
C. Gauthier-Umaña, J. Muñoz-Cabrera, M. Valderrama, A. Múnera and M. O. Nava-Mesa	Acute Effects of Two Different Species of Amyloid-β on Oscillatory Activity and Synaptic Plasticity in the Commissural CA3-CA1 Circuit of the Hippocampus	2020	Neural Plast	x								x			x						x			Aβ25-35, Aβ1-40	
S. Gelman, J. Palma, G. Tombaugh and A. Ghavami	Differences in Synaptic Dysfunction Between rTg4510 and APP/PS1 Mouse Models of Alzheimer's Disease	2018	J Alzheimers Dis				x			rTg4510 , APPswe/PS1M146L		x			x						x		x		
S. Gengler, V. A. Gault, P. Harriott and C. Hölscher	Impairments of hippocampal synaptic plasticity induced by aggregated β-amyloid (25-35) are dependent on stimulation-protocol and genetic background	2007	Exp Brain Res			x						x			x						x			Aβ25-35	
S. Gengler, A. Hamilton and C. Hölscher	Synaptic plasticity in the hippocampus of a APP/PS1 mouse model of Alzheimer's disease is impaired in old but not young mice	2010	PLoS One		x					APP/PS1-21		x			x						x		x		
J. Giacchino, J. R. Criado, D. Games and S. Henriksen	In vivo synaptic transmission in young and aged amyloid precursor protein transgenic mice	2000	Brain Res		x					PD/APP		x			x						x	x			
B. Gong, Z. Cao, P. Zheng, O. V. Vitolo, S. Liu, A. Staniszewski, et al.	Ubiquitin hydrolase Uch-L1 rescues β-amyloid-induced decreases in synaptic function and contextual memory	2006	Cell		x		x			APP/PS1		x		PKA, CREB	x			x		x	x			Aβ1-42	LDN
B. Gong, O. V. Vitolo, F. Trinchese, S. Liu, M. Shelanski and O. Arancio	Persistent improvement in synaptic and cognitive functions in an Alzheimer mouse model after rolipram treatment	2004	J Clin Invest		x		x			APP/PS1		x		PKA, CREB	x			x		x	x				Rolipram

General Information				Used Model						Genetic Modification		Investigated Neurotransmission		Signalling Pathway	Functional Analysis			Structural Analysis		Behavioral Experiments	Plasticity			Investigated APP fragments and domains	Investigated Substance, Intervention or Condition
Authors	Title	Year	Journal	In Vivo Rat	In Vivo Mouse	Ex Vivo Rat	Ex Vivo Mouse	In Vitro	Human	Transgen Line	APP-Related Viral Modifications	Excitiation	Inhibition		Field Potential Recordings	Whole Cell Recordings	Imaging	Light Microscopy	Electron Microscopy		LTP	LTD	STP		
Y. Goto, T. Niidome, H. Hongo, A. Akaike, T. Kihara and H. Sugimoto	Impaired muscarinic regulation of excitatory synaptic transmission in the APPswe/PS1dE9 mouse model of Alzheimer's disease	2008	Eur J Pharmacol				x			APPswe/PS1ΔE9		x			x								x		Physiostigmin
Z. Gu and J. L. Yakel	Timing-dependent septal cholinergic induction of dynamic hippocampal synaptic plasticity	2011	Neuron			x						x			x						x		x	Aβ1-42	
W. Gulisano, M. Melone, D. D. Li Puma, M. R. Tropea, A. Palmeri, O. Arancio, et al.	The effect of amyloid-β peptide on synaptic plasticity and memory is influenced by different isoforms, concentrations, and aggregation status	2018	Neurobiol Aging		x		x					x			x					x	x			Aβ1-42, Aβ1-40	
W. Gulisano, M. Melone, C. Ripoli, M. R. Tropea, D. D. Li Puma, S. Giunta, et al.	Neuromodulatory Action of Picomolar Extracellular Aβ42 Oligomers on Presynaptic and Postsynaptic Mechanisms Underlying Synaptic Function and Memory	2019	J Neurosci		x		x	OTC		APP-KO		x		NO - cGMP - PKG	x	x			x	x	x		x	Aβ1-42	
I. Gureviciene, S. Ikonen, K. Gurevicius, A. Sarkaki, T. van Groen, R. Pussinen, et al.	Normal induction but accelerated decay of LTP in APP + PS1 transgenic mice	2004	Neurobiol Dis		x		x			APP695swe/PS1		x			x					x	x		x		
W. N. Han, C. Hölscher, L. Yuan, W. Yang, X. H. Wang, M. N. Wu and J. S. Qi	Liraglutide protects against amyloid-β protein-induced impairment of spatial learning and memory in rats	2013	Neurobiol Aging	x								x			x					x	x		x	Aβ25-35	Liraglutide
A. Harmeier, C. Wozny, B. R. Rost, L. M. Munter, H. Hua, O. Georgiev, et al.	Role of amyloid-β glycine 33 in oligomerization, toxicity, and neuronal plasticity	2009	J Neurosci			x		Primary Cells				x			x						x			Aβ1-42, Aβ1-42 G33	
D. Hefter, M. Kaiser, S. W. Weyer, I. E. Papageorgiou, M. Both, O. Kann, et al.	Amyloid Precursor Protein Protects Neuronal Network Function after Hypoxia via Control of Voltage-Gated Calcium Channels	2016	J Neurosci				x			APP-KO, APPsα-KI		x			x	x	x						x		Hypoxia
D. Hermann, M. Both, U. Ebert, G. Gross, H. Schoemaker, A. Draguhn, et al.	Synaptic transmission is impaired prior to plaque formation in amyloid precursor protein-overexpressing mice without altering behaviorally-correlated sharp wave-ripple complexes	2009	Neuroscience				x			Tg2576		x			x								x		
D. Hermann, M. Mezler, M. K. Müller, K. Wicke, G. Gross, A. Draguhn, et al.	Synthetic Aβ oligomers (Aβ(1-42) globulomer) modulate presynaptic calcium currents: prevention of Aβ-induced synaptic deficits by calcium channel blockers	2013	Eur J Pharmacol					OTC				x			x									Aβ1-42	
M. Hick, U. Herrmann, S. W. Weyer, J. P. Mallm, J. A. Tschäpe, M. Borgers, et al.	Acute function of secreted amyloid precursor protein fragment APPsα in synaptic plasticity	2015	Acta Neuropathol		x		x			NexCre - APP/APLP2-KO		x			x	x		x		x	x				
H. Hillen, S. Barghorn, A. Striebinger, B. Labkovsky, R. Müller, V. Nimmrich, et al.	Generation and therapeutic efficacy of highly oligomer-specific β-amyloid antibodies	2010	J Neurosci		x			Primary Cells, OTC		APP-L		x			x			x		x					Aβ-oligo antibody
C. Holscher, S. Gengler, V. A. Gault, P. Harriott and H. A. Mallot	Soluble β-amyloid[25-35] reversibly impairs hippocampal synaptic plasticity and spatial learning	2007	Eur J Pharmacol	x								x			x					x	x			Aβ25-35	
A. Y. Hsia, E. Masliah, L. McConlogue, G. Q. Yu, G. Tatsuno, K. Hu, et al.	Plaque-independent disruption of neural circuits in Alzheimer's disease mouse models	1999	Proc Natl Acad Sci				x			APPswe		x			x	x		x			x				
N. W. Hu, I. Klyubin, R. Anwyl and M. J. Rowan	GluN2B subunit-containing NMDA receptor antagonists prevent Aβ-mediated synaptic plasticity disruption in vivo	2009	Proc Natl Acad Sci	x								x		TNFR1	x						x			Aβ1-42	
N. W. Hu, A. J. Nicoll, D. Zhang, A. J. Mably, T. O'Malley, S. A. Purro, et al.	mGlu5 receptors and cellular prion protein mediate amyloid-β-facilitated synaptic long-term depression in vivo	2014	Nat Commun	x								x			x						x	x		Aβ1-42	Anti-PrP Antibody
Y. S. Hu, P. Xu, G. Pigino, S. T. Brady, J. Larson and O. Lazarov	Complex environment experience rescues impaired neurogenesis, enhances synaptic plasticity, and attenuates neuropathology in familial Alzheimer's disease-linked APPswe/PS1DeltaE9 mice	2010	Faseb j				x			APPswe/PS1ΔE9		x			x						x				Environmental Enrichment
S. Huang, H. Tong, M. Lei, M. Zhou, W. Guo, G. Li, et al.	Astrocytic glutamatergic transporters are involved in Aβ-induced synaptic dysfunction	2018	Brain Res				x					x			x						x			Aβ1-42	TFB- and DL-TBOA
T. Isono, N. Yamashita, M. Obara, T. Araki, F. Nakamura, Y. Kamiya, et al.	Amyloid-β_{25-35} induces impairment of cognitive function and long-term potentiation through phosphorylation of collapsin response mediator protein 2	2013	Neurosci Res		x		x					x		CRMP2	x					x	x			Aβ25-35	
W. Jing, F. Guo, L. Cheng, J. F. Zhang and J. S. Qi	Arginine vasopressin prevents amyloid β protein-induced impairment of long-term potentiation in rat hippocampus in vivo	2009	Neurosci Lett	x								x			x						x			Aβ25-35	AVP
T. Jolas, X. S. Zhang, Q. Zhang, G. Wong, R. Del Vecchio, L. Gold and T. Priestley	Long-term potentiation is increased in the CA1 area of the hippocampus of APP(swe/ind) CRND8 mice	2002	Neurobiol Dis		x		x			CRND8		x	x		x	x		x			x				
J. H. Jung, K. An, O. B. Kwon, H. S. Kim and J. H. Kim	Pathway-specific alteration of synaptic plasticity in Tg2576 mice	2011	Mol Cells				x			Tg2576		x			x						x				
K. S. Kaleka and N. Z. Gerges	Neurogranin restores amyloid β-mediated synaptic transmission and long-term potentiation deficits	2016	Exp Neurol					OTC			APPswe/Ind-IRES-GFP	x		Ng - CAMKII	x	x					x			Aβ1-42	Neurogranin
F. Kamenetz, T. Tomita, H. Hsieh, G. Seabrook, D. Borchelt, T. Iwatsubo, et al	APP processing and synaptic function	2003	Neuron					OTC		APPswe		x			x	x		x			x			Aβ1-40, Aβ1-42	
J. Kim, S. Kim, H. Kim, I. W. Hwang, S. Bae, S. Karki, et al.	MDGA1 negatively regulates amyloid precursor protein-mediated synapse inhibition in the hippocampus	2022	Proc Natl Acad Sci		x		x	Primary Cells, OTC		APPf/f, MDGA1f/f, MDGA2f/f	MDGA1 MAM, APP, Nlgn2	x	x	MDGA1		x		x		x					
J. H. Kim, R. Anwyl, Y. H. Suh, M. B. Djamgoz and M. J. Rowan	Use-dependent effects of amyloidogenic fragments of (β)-amyloid precursor protein on synaptic plasticity in rat hippocampus in vivo	2001	J Neurosci	x								x			x						x	x		Aβ1-42, C-term. βAPP	
R. Kimura, L. Devi and M. Ohno	Partial reduction of BACE1 improves synaptic plasticity, recent and remote memories in Alzheimer's disease transgenic mice	2010	J Neurochem		x		x			5XFAD APP/PS1 (Tg6799), BACE+/- 5XFAD+/-		x		BDNF-TrkB	x					x	x				
R. Kimura, D. MacTavish, J. Yang, D. Westaway and J. H. Jhamandas	β amyloid-induced depression of hippocampal long-term potentiation is mediated through the amylin receptor	2012	J Neurosci				x			CRND8		x		Amylin Receptor	x						x			Aβ1-42	h-Amylin, AC253

General Information				Used Model						Genetic Modification		Investigated Neurotransmission		Signalling Pathway	Functional Analysis			Structural Analysis		Behavioral Experiments	Plasticity			Investigated APP fragments and domains	Investigated Substance, Intervention or Condition
Authors	Title	Year	Journal	In Vivo Rat	In Vivo Mouse	Ex Vivo Rat	Ex Vivo Mouse	In Vitro	Human	Transgen Line	APP-Related Viral Modifications	Excitiation	Inhibition		Field Potential Recordings	Whole Cell Recordings	Imaging	Light Microscopy	Electron Microscopy		LTP	LTD	STP		
R. Kimura, D. MacTavish, J. Yang, D. Westaway and J. H. Jhamandas	Pramlintide Antagonizes β Amyloid (Aβ)- and Human Amylin-Induced Depression of Hippocampal Long-Term Potentiation	2017	Mol Neurobiol				x			CRND8		x		Amylin Receptor	x						x			Aβ1-42	h-Amylin, Pramlintide
R. Kimura and M. Ohno	Impairments in remote memory stabilization precede hippocampal synaptic and cognitive failures in 5XFAD Alzheimer mouse model	2009	Neurobiol Dis		x		x			5XFAD APP/PS1		x			x					x	x		x		
M. Klevanski, U. Herrmann, S. W. Weyer, R. Fol, N. Cartier, D. P. Wolfer, et al.	The APP Intracellular Domain Is Required for Normal Synaptic Morphology, Synaptic Plasticity, and Hippocampus-Dependent Behavior	2015	J Neurosci		x		x			APLP2-KO, APPCT15-DM, APP/APLP2-DKO		x			x			x		x	x				
I. Klyubin, T. Ondrejcak, J. Hayes, W. K. Cullen, A. J. Mably, D. M. Walsh and M. J. Rowan	Neurotransmitter receptor and time dependence of the synaptic plasticity disrupting actions of Alzheimer's disease Aβ in vivo	2014	Philos Trans R Soc Lond B Biol Sci	x								x			x						x			Aβ1-42	Metoctramin
M. Knobloch, M. Farinelli, U. Konietzko, R. M. Nitsch and I. M. Mansuy	Aβ oligomer-mediated long-term potentiation impairment involves protein phosphatase 1-dependent mechanisms	2007	J Neurosci				x			arcAβ, APPswe/PS1		x		PP1	x						x			Aβ1-42	Tautomycin
S. Kootar, M. L. Frandemiche, G. Dhib, X. Mouska, T. Lorivel, G. Poupon-Silvestre, et al.	Identification of an acute functional cross-talk between amyloid-β and glucocorticoid receptors at hippocampal excitatory synapses	2018	Neurobiol Dis		x		x	Primary Cells				x		GR	x			x			x	x		Aβ1-42	C13
P. Koppensteiner, F. Trinchese, M. Fà, D. Puzzo, W. Gulisano, S. Yan, et al.	Time-dependent reversal of synaptic plasticity induced by physiological concentrations of oligomeric Aβ42: an early index of Alzheimer's disease	2016	Sci Rep		x		x	Primary Cells				x		p38-MAPK	x	x		x		x	x			Aβ1-42	SB203580
D. J. Koss, B. D. Drever, S. Stoppelkamp, G. Riedel and B. Platt	Age-dependent changes in hippocampal synaptic transmission and plasticity in the PLB1Triple Alzheimer mouse	2013	Cell Mol Life Sci				x			PLB1Triple		x			x			x			x	x	x		
S. Lam, A. S. Hérard, S. Boluda, F. Petit, S. Eddarkaoui, K. Cambon, et al.	Pathological changes induced by Alzheimer's brain inoculation in amyloid-β plaque-bearing mice	2022	Acta Neuropathol Commun		x		x			APPswe/PS1ΔE9		x						x		x				human Aβ	
F. Lanté, M. Chafai, E. F. Raymond, A. R. Pereira, X. Mouska, S. Kootar, et al.	Subchronic glucocorticoid receptor inhibition rescues early episodic memory and synaptic plasticity deficits in a mouse model of Alzheimer's disease	2015	Neuropsychopharmacology		x		x			Tg2576		x		GR	x	x				x	x	x			RU486
J. Larson, G. Lynch, D. Games and P. Seubert	Alterations in synaptic transmission and long-term potentiation in hippocampal slices from young and aged PDAPP mice	1999	Brain Res				x			PD/APP		x			x						x		x		
C. C. Lee, C. P. Chang, C. J. Lin, H. L. Lai, Y. H. Kao, S. J. Cheng, et al.	Adenosine Augmentation Evoked by an ENT1 Inhibitor Improves Memory Impairment and Neuronal Plasticity in the APP/PS1 Mouse Model of Alzheimer's Disease	2018	Mol Neurobiol		x		x			APPswe/PS1ΔE9		x		PKA, GSK3β	x			x		x	x				J4 (ENT1 inhibitor)
K. Lee, H. Kim, K. An, O. B. Kwon, S. Park, J. H. Cha, et al.	Replenishment of microRNA-188-5p restores the synaptic and cognitive deficits in 5XFAD Mouse Model of Alzheimer's Disease	2016	Sci Rep		x		x	Primary Cells		5XFAD		x		CREB	x	x		x		x	x			Aβ1-42	
S. H. Lee, J. Kang, A. Ho, H. Watanabe, V. Y. Bolshakov and J. Shen	APP Family Regulates Neuronal Excitability and Synaptic Plasticity but Not Neuronal Survival	2020	Neuron		x		x			APPxAPLP1xAPLP2-KO		x			x	x				x	x		x		
S. H. Lee, K. R. Kim, S. Y. Ryu, S. Son, H. S. Hong, I. Mook-Jung, et al.	Impaired short-term plasticity in mossy fiber synapses caused by mitochondrial dysfunction of dentate granule cells is the earliest synaptic deficit in a mouse model of Alzheimer's disease	2012	J Neurosci				x			Tg2576		x					x							Aβ1-42, Aβ1-40	
M. Lei, H. Xu, Z. Li, Z. Wang, T. T. O'Malley, D. Zhang, et al.	Soluble Aβ oligomers impair hippocampal LTP by disrupting glutamatergic/GABAergic balance	2016	Neurobiol Dis				x					x	x		x	x					x			Aβ(1–40)S26C	Levetiracetam, Topiramat
S. L. Lesuis, P. J. Lucassen and H. J. Krugers	Early life stress amplifies fear responses and hippocampal synaptic potentiation in the APPswe/PS1dE9 Alzheimer mouse model	2021	Neuroscience		x		x			APPswe/PS1ΔE9		x			x					x	x				Early life stress
L. Li, X. K. Tong, M. Hosseini Kahnouei, D. Vallerand, E. Hamel and H. Girouard	Impaired Hippocampal Neurovascular Coupling in a Mouse Model of Alzheimer's Disease	2021	Front Physiol				x			APPswe, APPInd		x			x						x		x		
S. Li, M. Jin, T. Koeglsperger, N. E. Shepardson, G. M. Shankar and D. J. Selkoe	Soluble Aβ oligomers inhibit long-term potentiation through a mechanism involving excessive activation of extrasynaptic NR2B-containing NMDA receptors	2011	J Neurosci				x	Primary Cells				x		p38-MAPK, ERK1,2, CREB	x	x	x	x			x			Aβ	
S. Li, M. Jin, D. Zhang, T. Yang, T. Koeglsperger, H. Fu and D. J. Selkoe	Environmental novelty activates β2-adrenergic signaling to prevent the impairment of hippocampal LTP by Aβ oligomers	2013	Neuron				x	Primary Cells				x			x						x			Aβ	Enriched Environment
T. Li, J. J. Jiao, Q. Su, C. Hölscher, J. Zhang, X. D. Yan, et al.	A GLP-1/GIP/Gcg receptor triagonist improves memory behavior, as well as synaptic transmission, neuronal excitability and Ca(2+) homeostasis in 3xTg-AD mice	2020	Neuropharmacology		x		x			3xTg		x				x	x	x		x	x				GLP-1/GIP/Gcg receptor triagonist
W. Li, J. Yu, Y. Liu, X. Huang, N. Abumaria, Y. Zhu, et al.	Elevation of brain magnesium prevents synaptic loss and reverses cognitive deficits in Alzheimer's disease mouse model	2014	Mol Brain		x		x			APPswe/PS1ΔE9		x		CREB	x	x		x	x	x				Aβ1-42	Magnesium
Y. Li, K. Zhu, N. Li, X. Wang, X. Xiao, L. Li, et al.	Reversible GABAergic dysfunction involved in hippocampal hyperactivity predicts early-stage Alzheimer disease in a mouse model	2021	Alzheimers Res Ther				x			5XFAD		x	x			x		x	x						
L. Liu, I. J. Orozco, E. Planel, Y. Wen, A. Bretteville, P. Krishnamurthy, et al.	A transgenic rat that develops Alzheimer's disease-like amyloid pathology, deficits in synaptic plasticity and cognitive impairment	2008	Neurobiol Dis	x		x				APP/ PS1 mut		x			x					x	x				
H. Ma, S. Lesné, L. Kotilinek, J. V. Steidl-Nichols, M. Sherman, L. Younkin, et al.	Involvement of β-site APP cleaving enzyme 1 (BACE1) in amyloid precursor protein-mediated enhancement of memory and activity-dependent synaptic plasticity	2007	Proc Natl Acad Sci		x		x			TgAPP, Tg-BACE KO, Tg2576		x			x					x	x				
T. Ma, Y. Chen, V. Vingtdeux, H. Zhao, B. Viollet, P. Marambaud and E. Klann	Inhibition of AMP-activated protein kinase signaling alleviates impairments in hippocampal synaptic plasticity induced by amyloid β	2014	J Neurosci				x			APP/PS1		x		AMPK, eEF2K	x						x			Aβ1-42	

General Information				Used Model						Genetic Modification		Investigated Neurotransmission		Signalling Pathway	Functional Analysis			Structural Analysis		Behavioral Experiments	Plasticity			Investigated APP fragments and domains	Investigated Substance, Intervention or Condition
Authors	Title	Year	Journal	In Vivo Rat	In Vivo Mouse	Ex Vivo Rat	Ex Vivo Mouse	In Vitro	Human	Transgen Line	APP-Related Viral Modifications	Excitiation	Inhibition		Field Potential Recordings	Whole Cell Recordings	Imaging	Light Microscopy	Electron Microscopy		LTP	LTD	STP		
T. Ma, X. Du, J. E. Pick, G. Sui, M. Brownlee and E. Klann	Glucagon-like peptide-1 cleavage product GLP-1(9-36) amide rescues synaptic plasticity and memory deficits in Alzheimer's disease model mice	2012	J Neurosci		x		x			APP/PS1		x		GSK3β	x					x	x			Aβ1-42	GLP-1(9-36)
I. Maezawa, B. Zou, J. Di Lucente, W. S. Cao, C. Pascual, S. Weerasekara, et al.	The Anti-Amyloid-β and Neuroprotective Properties of a Novel Tricyclic Pyrone Molecule	2017	J Alzheimers Dis		x		x			Tg6799 5xFAD		x			x					x	x			Aβ	TP70
M. Manczak, R. Kandimalla, X. Yin and P. H. Reddy	Hippocampal mutant APP and amyloid β-induced cognitive decline, dendritic spine loss, defective autophagy, mitophagy and mitochondrial abnormalities in a mouse model of Alzheimer's disease	2018	Hum Mol Genet		x		x			Tg2576		x						x		x					
D. Mango and R. Nisticò	Role of ASIC1a in Aβ-induced synaptic alterations in the hippocampus	2018	Pharmacol Res				x			Tg2576		x		ASIC (acid sensitive ion channels)		x						x		Aβ1-42	PcTx1
A. Martín-Belmonte, C. Aguado, R. Alfaro-Ruiz, J. L. Albasanz, M. Martín, A. E. Moreno-Martínez, et al.	The Density of Group I mGlu(5) Receptors Is Reduced along the Neuronal Surface of Hippocampal Cells in a Mouse Model of Alzheimer's Disease	2021	Int J Mol Sci		x					APP/PS1		x							x						
A. Martín-Belmonte, C. Aguado, R. Alfaro-Ruíz, A. E. Moreno-Martínez, L. de la Ossa, J. Martínez-Hernández, et al.	Reduction in the neuronal surface of post and presynaptic GABA(B) receptors in the hippocampus in a mouse model of Alzheimer's disease	2020	Brain Pathol		x					APP/PS1			x					x	x						
P. L. McClean, J. Jalewa and C. Hölscher	Prophylactic liraglutide treatment prevents amyloid plaque deposition, chronic inflammation and memory impairment in APP/PS1 mice	2015	Behav Brain Res		x					APPswe/PS1ΔE9		x			x			x		x	x		x		Liraglutide
E. Medawar, T. A. Benway, W. Liu, T. A. Hanan, P. Haslehurst, O. T. James, et al.	Effects of rising amyloidβ levels on hippocampal synaptic transmission, microglial response and cognition in APP(Swe)/PSEN1(M146V) transgenic mice	2019	EBioMedicine		x		x			TASTPM		x	x		x	x		x		x	x		x		
A. Mehr, M. Hick, S. Ludewig, M. Müller, U. Herrmann, J. von Engelhardt, et al.	Lack of APP and APLP2 in GABAergic Forebrain Neurons Impairs Synaptic Plasticity and Cognition	2020	Cereb Cortex		x		x	Primary Cells		APPflox/flox/APLP2-/-/Dlxcre+		x	x		x	x		x		x	x				
Y. Meng, L. Ding, H. Zhang, W. Yin, Y. Yan and Y. Cao	Immunization of Tg-APPswe/PSEN1dE9 mice with Aβ3-10-KLH vaccine prevents synaptic deficits of Alzheimer's disease	2017	Behav Brain Res				x			APP/PS1		x						x	x						Aβ3-10-KLH (vaccine)
B. Midthune, S. H. Tyan, J. J. Walsh, F. Sarsoza, S. Eggert, P. R. Hof, et al.	Deletion of the amyloid precursor-like protein 2 (APLP2) does not affect hippocampal neuron morphology or function	2012	Mol Cell Neurosci				x	Primary Cells		APLP2-/-		x			x			x			x				
S. S. Min, J. An, J. H. Lee, G. H. Seol, J. H. Im, H. S. Kim, et al.	Neuregulin-1 prevents amyloid β-induced impairment of long-term potentiation in hippocampal slices via ErbB4	2011	Neurosci Lett				x					x		Neuregulin 1 - ErbB4	x						x			Aβ1-42	Neuregulin
T. Nagata, T. Tomiyama, H. Mori, T. Yaguchi and T. Nishizaki	DCP-LA neutralizes mutant amyloid β peptide-induced impairment of long-term potentiation and spatial learning	2010	Behav Brain Res	x								x			x					x	x			Aβ1-42E22Δ, Aβ1-42	DCP-LA
Y. Nakagami, S. Nishimura, T. Murasugi, T. Kubo, I. Kaneko, M. Meguro, et al.	A novel compound RS-0466 reverses β-amyloid-induced cytotoxicity through the Akt signaling pathway in vitro	2002	Eur J Pharmacol			x		Primary Cells				x		Akt	x						x			Aβ1-42, Aβ1-40	RS-0466
V. Nimmrich, K. G. Reymann, M. Strassburger, U. H. Schöder, G. Gross, A. Hahn, et al.	Inhibition of calpain prevents NMDA-induced cell death and β-amyloid-induced synaptic dysfunction in hippocampal slice cultures	2010	Br J Pharmacol			x		OTC				x			x									Aβ	A-705253
I. Nomura, N. Kato, T. Kita and H. Takechi	Mechanism of impairment of long-term potentiation by amyloid β is independent of NMDA receptors or voltage-dependent calcium channels in hippocampal CA1 pyramidal neurons	2005	Neurosci Lett			x						x				x					x		x	Aβ1-42	
I. Nomura, H. Takechi and N. Kato	Intraneuronally injected amyloid β inhibits long-term potentiation in rat hippocampal slices	2012	J Neurophysiol			x						x				x					x			Aβ1-42, Aβ1-40	SB203580
E. O'Hare, R. Jeggo, E. M. Kim, B. Barbour, J. S. Walczak, P. Palmer, et al.	Lack of support for bexarotene as a treatment for Alzheimer's disease	2016	Neuropharmacology	x	x	x				5XFAD		x			x					x	x			Aβ1-42, Aβ1-40	Bexarotene
T. Ochiishi, M. Kaku, K. Kiyosue, M. Doi, T. Urabe, N. Hattori, et al.	New Alzheimer's disease model mouse specialized for analyzing the function and toxicity of intraneuronal Amyloid β oligomers	2019	Sci Rep		x		x			Aβ1-42-GFP		x			x			x	x	x	x				
T. G. Oliveira, R. B. Chan, H. Tian, M. Laredo, G. Shui, A. Staniszewski, et al.	Phospholipase d2 ablation ameliorates Alzheimer's disease-linked synaptic dysfunction and cognitive deficits	2010	J Neurosci		x		x	Primary Cells		Tg2576/pld2-/-		x		PLD2	x					x	x				
K. M. Olsen and M. Sheng	NMDA receptors and BAX are essential for Aβ impairment of LTP	2012	Sci Rep				x			BAX-/-		x			x						x	x		Aβ1-42	Ro25-6981
T. Ondrejcak, I. Klyubin, N. W. Hu, T. T. O'Malley, G. T. Corbett, R. Winters, et al.	Tau and Amyloid β Protein in Patient-Derived Aqueous Brain Extracts Act Concomitantly to Disrupt Long-Term Potentiation in Vivo	2023	J Neurosci	x								x			x						x			Aβ1-42	
A. L. Orr, J. E. Hanson, D. Li, A. Klotz, S. Wright, D. Schenk, et al.	β-Amyloid inhibits E-S potentiation through suppression of cannabinoid receptor 1-dependent synaptic disinhibition	2014	Neuron			x						x	x		x	x					x			Aβ1-42	
T. Oyelami, A. Bondt, I. V. den Wyngaert, K. V. Hoorde, L. Hoskens, H. Shaban, et al.	Age-dependent concomitant changes in synaptic dysfunction and GABAergic pathway in the APP/PS1 mouse model	2016	Acta Neurobiol Exp				x			APP/PS1		x	x		x						x		x		
M. Pascual-Lucas, S. Viana da Silva, M. Di Scala, C. Garcia-Barroso, G. González-Aseguinolaza, C. Mulle, et al.	Insulin-like growth factor 2 reverses memory and synaptic deficits in APP transgenic mice	2014	EMBO Mol Med		x		x	Primary Cells	x	Tg2576		x				x		x		x					IGF2
A. Patel, R. Kimura, W. Fu, R. Soudy, D. MacTavish, D. Westaway, et al.	Genetic Depletion of Amylin/Calcitonin Receptors Improves Memory and Learning in Transgenic Alzheimer's Disease Mouse Models	2021	Mol Neurobiol		x		x			TgCRND8, 5xFAD		x			x					x	x				Amylin-KO
G. Piccioni, N. Maisto, A. d'Ettorre, G. Strimpakos, R. Nisticò, V. Triaca and D. Mango	Switch to phagocytic microglia by CSFR1 inhibition drives amyloid-β clearance from glutamatergic terminals rescuing LTP in acute hippocampal slices	2024	Transl Psychiatry				x					x			x	x		x			x			Aβ1-42	PLX3397, Minocycline

General Information				Used Model						Genetic Modification		Investigated Neurotransmission		Signalling Pathway	Functional Analysis			Structural Analysis		Behavioral Experiments	Plasticity			Investigated APP fragments and domains	Investigated Substance, Intervention or Condition
Authors	Title	Year	Journal	In Vivo Rat	In Vivo Mouse	Ex Vivo Rat	Ex Vivo Mouse	In Vitro	Human	Transgen Line	APP-Related Viral Modifications	Excitiation	Inhibition		Field Potential Recordings	Whole Cell Recordings	Imaging	Light Microscopy	Electron Microscopy		LTP	LTD	STP		
B. Portal, M. Södergren, I. B. T. Parés, R. Giraud, N. G. Metzendorf, G. Hultqvist, et al.	Early Astrocytic Dysfunction Is Associated with Mistuned Synapses as well as Anxiety and Depressive-Like Behavior in the AppNL-F Mouse Model of Alzheimer's Disease	2024	J Alzheimers Dis		x		x			APP NL-F		x	x		x	x				x	x				Deprenyl, Ketamine
P. A. Pousinha, X. Mouska, E. F. Raymond, C. Gwizdek, G. Dhib, G. Poupon, et al.	Physiological and pathophysiological control of synaptic GluN2B-NMDA receptors by the C-terminal domain of amyloid precursor protein	2017	Elife			x		Primary Cells			AICD-KI	x			x	x					x	x		AICD	
L. Pradier, V. Blanchard-Brégeon, A. Bohme, T. Debeir, J. Menager, P. Benoit, et al.	SAR228810: an antibody for protofibrillar amyloid β peptide designed to reduce the risk of amyloid-related imaging abnormalities (ARIA)	2018	Alzheimers Res Ther		x		x	Primary Cells				x				x								Aβ1-42	SAR228810
K. A. Price, M. Varghese, A. Sowa, F. Yuk, H. Brautigam, M. E. Ehrlich and D. L. Dickstein	Altered synaptic structure in the hippocampus in a mouse model of Alzheimer's disease with soluble amyloid-β oligomers and no plaque pathology	2014	Mol Neurodegener				x			APP E693Q		x						x							
C. Priller, T. Bauer, G. Mitteregger, B. Krebs, H. A. Kretzschmar and J. Herms	Synapse formation and function is modulated by the amyloid precursor protein	2006	J Neurosci				x	Primary Cells		APP-KO		x				x		x							
D. Puzzo, L. Privitera, E. Leznik, M. Fà, A. Staniszewski, A. Palmeri and O. Arancio	Picomolar amyloid-β positively modulates synaptic plasticity and memory in hippocampus	2008	J Neurosci		x		x					x			x	x				x	x			Aβ1-42	
Y. Qi, I. Klyubin, S. C. Harney, N. Hu, W. K. Cullen, M. K. Grant, et al.	Longitudinal testing of hippocampal plasticity reveals the onset and maintenance of endogenous human Aβ-induced synaptic dysfunction in individual freely behaving pre-plaque transgenic rats: rapid reversal by anti-Aβ agents	2014	Acta Neuropathol Commun	x		x				APP751+Swe+India na		x			x	x					x		x		
G. Rammes, A. Hasenjäger, K. Sroka-Saidi, J. M. Deussing and C. G. Parsons	Therapeutic significance of NR2B-containing NMDA receptors and mGluR5 metabotropic glutamate receptors in mediating the synaptotoxic effects of β-amyloid oligomers on long-term potentiation (LTP) in murine hippocampal slices	2011	Neuropharmacology				x					x			x	x					x			Aβ1-42	
G. Rammes, F. Seeser, K. Mattusch, K. Zhu, L. Haas, M. Kummer, et al.	The NMDA receptor antagonist Radiprodil reverses the synaptotoxic effects of different amyloid-β (Aβ) species on long-term potentiation (LTP)	2018	Neuropharmacology				x					x			x	x		x			x			Aβ1-42, Aβ1-40	Radiprodil
C. R. Raymond, D. R. Ireland and W. C. Abraham	NMDA receptor regulation by amyloid-β does not account for its inhibition of LTP in rat hippocampus	2003	Brain Res			x						x			x						x	x		Aβ1-40	
Z. Rezaei Asl, G. Sepehri and M. Salami	Probiotic treatment improves the impaired spatial cognitive performance and restores synaptic plasticity in an animal model of Alzheimer's disease	2019	Behav Brain Res	x								x			x					x	x			Aβ1-42	Probiotics
H. C. Rice, D. de Malmazet, A. Schreurs, S. Frere, I. Van Molle, A. N. Volkov, et al.	Secreted amyloid-β precursor protein functions as a GABA(B)R1a ligand to modulate synaptic transmission	2019	Science		x		x	Primary Cells		APP/APLP1-KO		x	x		x	x	x						x	sAPPα	APP 17 mer
M. C. Richter, S. Ludewig, A. Winschel, T. Abel, C. Bold, L. R. Salzburger, et al.	Distinct in vivo roles of secreted APP ectodomain variants APPsα and APPsβ in regulation of spine density, synaptic plasticity, and cognition	2018	Embo j		x		x	Primary Cells		APP/APLP2-KO		x			x			x		x	x		x	sAPPα (+β)	BTX
U. M. Ricoy, P. Mao, M. Manczak, P. H. Reddy and M. E. Frerking	A transgenic mouse model for Alzheimer's disease has impaired synaptic gain but normal synaptic dynamics	2011	Neurosci Lett				x			APPswe/PS1A246E		x			x								x		
C. Ripoli, S. Cocco, D. D. Li Puma, R. Piacentini, A. Mastrodonato, F. Scala, et al.	Intracellular accumulation of amyloid-β (Aβ) protein plays a major role in Aβ-induced alterations of glutamatergic synaptic transmission and plasticity	2014	J Neurosci				x	Primary Cells				x			x	x		x			x			Aβ1-42	
E. D. Roberson, B. Halabisky, J. W. Yoo, J. Yao, J. Chin, F. Yan, et al.	Amyloid-β/Fyn-induced synaptic, network, and cognitive impairments depend on tau levels in multiple mouse models of Alzheimer's disease	2011	J Neurosci		x		x			TASD41/Fyn/Tau-/-, hAPPJ9/Tau-/-, SOD1G93A/Tau-/-, NTG/Tau+/+		x	x	Fyn	x	x		x		x	x				
S. Roder, L. Danober, M. F. Pozza, K. Lingenhoehl, K. H. Wiederhold and H. R. Olpe	Electrophysiological studies on the hippocampus and prefrontal cortex assessing the effects of amyloidosis in amyloid precursor protein 23 transgenic mice	2003	Neuroscience		x		x			APP23(-/+)		x			x						x				
M. Rolland, R. Powell, M. Jacquier-Sarlin, S. Boisseau, R. Reynaud-Dulaurier, J. Martinez-Hernandez, et al.	Effect of Aβ Oligomers on Neuronal APP Triggers a Vicious Cycle Leading to the Propagation of Synaptic Plasticity Alterations to Healthy Neurons	2020	J Neurosci				x	Primary Cells		APP-KO	hAPPswe-HA-EYFP	x			x	x		x			x			Aβ1-42	4G8 antibody
M. Ruiter, L. J. Herstel and C. J. Wierenga	Reduction of Dendritic Inhibition in CA1 Pyramidal Neurons in Amyloidosis Models of Early Alzheimer's Disease	2020	J Alzheimers Dis				x	OTC		APPNL-F-G/GAD65-GFP		x	x			x		x						Aβ1-42	
C. L. Russell, S. Semerdjieva, R. M. Empson, B. M. Austen, P. W. Beesley and P. Alifragis	Amyloid-β acts as a regulator of neurotransmitter release disrupting the interaction between synaptophysin and VAMP2	2012	PLoS One			x		Primary Cells				x		Syn/VAMP2	x			x			x			Aβ1-42	6.00E+10
I. Sánchez-Rodríguez, S. Djebari, S. Temprano-Carazo, D. Vega-Avelaira, R. Jiménez-Herrera, G. Iborra-Lázaro, et al.	Hippocampal long-term synaptic depression and memory deficits induced in early amyloidopathy are prevented by enhancing G-protein-gated inwardly rectifying potassium channel activity	2020	J Neurochem		x		x					x		GirK	x			x		x	x	x		Aβ1-42	ML297
I. Sánchez-Rodríguez, A. Gruart, J. M. Delgado-García, L. Jiménez-Díaz and J. D. Navarro-López	Role of GirK Channels in Long-Term Potentiation of Synaptic Inhibition in an In Vivo Mouse Model of Early Amyloid-β Pathology	2019	Int J Mol Sci		x							x	x	GirK	x						x			Aβ1-42	ML297
I. Sánchez-Rodríguez, S. Temprano-Carazo, A. Nájera, S. Djebari, J. Yajeya, A. Gruart, et al.	Activation of G-protein-gated inwardly rectifying potassium (Kir3/GirK) channels rescues hippocampal functions in a mouse model of early amyloid-β pathology	2017	Sci Rep		x							x		GirK	x					x	x		x	Aβ1-42	ML297

General Information				Used Model						Genetic Modification		Investigated Neurotransmission		Signalling Pathway	Functional Analysis			Structural Analysis		Behavioral Experiments	Plasticity			Investigated APP fragments and domains	Investigated Substance, Intervention or Condition
Authors	Title	Year	Journal	In Vivo Rat	In Vivo Mouse	Ex Vivo Rat	Ex Vivo Mouse	In Vitro	Human	Transgen Line	APP-Related Viral Modifications	Excitiation	Inhibition		Field Potential Recordings	Whole Cell Recordings	Imaging	Light Microscopy	Electron Microscopy		LTP	LTD	STP		
J. L. Sanderson, R. K. Freund, J. A. Gorski and M. L. Dell'Acqua	β-Amyloid disruption of LTP/LTD balance is mediated by AKAP150-anchored PKA and Calcineurin regulation of Ca(2+)-permeable AMPA receptors	2021	Cell Rep				x			AKAP150ΔPIX, AKAP150ΔPKA		x		AKAP-CaN, AKAP-PKA	x	x		x			x			Aβ1-42	
A. G. Sandoval-Hernández, L. Buitrago, H. Moreno, G. P. Cardona-Gómez and G. Arboleda	Role of Liver X Receptor in AD Pathophysiology	2015	PLoS One		x		x			3x Tg-AD		x		LXR	x			x		x	x			Aβ1-42	GW3965
D. Schlenzig, R. Rönicke, H. Cynis, H. H. Ludwig, E. Scheel, K. Reymann, et al.	N-Terminal pyroglutamate formation of Aβ38 and Aβ40 enforces oligomer formation and potency to disrupt hippocampal long-term potentiation	2012	J Neurochem				x					x			x						x			Aβ1-38, Aβ1-40, pGlu-Aβ3-38, pGlu-Aβ3-40, Aβ1-42, pGlu-Aβ1-42, ADan, pGlu-ADan	
C. Schmidt, E. Lepsverdize, S. L. Chi, A. M. Das, S. V. Pizzo, A. Dityatev and M. Schachner	Amyloid precursor protein and amyloid β-peptide bind to ATP synthase and regulate its activity at the surface of neural cells	2008	Mol Psychiatry				x	Primary Cells		APP23		x		ATP synthase subunit α	x			x			x			Aβ1-40	Picrotoxin, IF1
G. R. Seabrook, D. W. Smith, B. J. Bowery, A. Easter, T. Reynolds, S. M. Fitzjohn, et al.	Mechanisms contributing to the deficits in hippocampal synaptic plasticity in mice lacking amyloid precursor protein	1999	Neuropharmacology				x			APP-KO		x	x		x	x		x			x		x		
H. J. Seo, J. E. Park, S. M. Choi, T. Kim, S. H. Cho, K. H. Lee, et al.	Inhibitory Neural Network's Impairments at Hippocampal CA1 LTP in an Aged Transgenic Mouse Model of Alzheimer's Disease	2021	Int J Mol Sci		x		x			5xFAD		x	x	NRG1-ErbB4	x					x	x				PD158780
M. Shabani, M. Haghani, P. E. Tazangi, M. Bayat, S. M. Shid Moosavi and H. Ranjbar	Netrin-1 improves the amyloid-β-mediated suppression of memory and synaptic plasticity	2017	Brain res Bull	x								x			x					x	x		x	Aβ1-42	Netrin 1
G. M. Shankar, B. L. Bloodgood, M. Townsend, D. M. Walsh, D. J. Selkoe and B. L. Sabatini	Natural oligomers of the Alzheimer amyloid-β protein induce reversible synapse loss by modulating an NMDA-type glutamate receptor-dependent signaling pathway	2007	J Neurosci					OTC				x		Calcineurin-cofilin		x	x	x						Aβ	FK506, S3D
Y. Shen, M. Tian, Y. Zheng, F. Gong, A. K. Y. Fu and N. Y. Ip	Stimulation of the Hippocampal POMC/MC4R Circuit Alleviates Synaptic Plasticity Impairment in an Alzheimer's Disease Model	2016	Cell Rep				x	Primary Cells		APP/PS1, POMCcre, Mc4rtb		x		POMC/MC4R-PKA/CREB	x	x		x			x			Aβ	D-Tyr
B. Shu, X. Zhang, G. Du, Q. Fu and L. Huang	MicroRNA-107 prevents amyloid-β-induced neurotoxicity and memory impairment in mice	2018	Int J Mol Sci		x		x					x		BDNF-TrkB and AKT	x					x	x			Aβ1-42	miR-107 mimic
J. P. Smith, V. Lal, D. Bowser, R. Cappai, C. L. Masters and G. D. Ciccotosto	Stimulus pattern dependence of the Alzheimer's disease amyloid-β 42 peptide's inhibition of long term potentiation in mouse hippocampal slices	2009	Brain Res				x					x			x						x		x	Aβ1-42	
W. M. Snow, K. Oikawa, J. Djordjevic and B. C. Albensi	Strain differences in hippocampal synaptic dysfunction in the TgCRND8 mouse model of Alzheimer's disease: Implications for improving translational capacity	2019	Mol Cell Neurosci				x			TgCRND8		x			x						x		x		
A. Söderman, J. D. Mikkelsen, M. J. West, D. Z. Christensen and M. S. Jensen	Activation of nicotinic α(7) acetylcholine receptor enhances long term potentation in wild type mice but not in APP(swe)/PS1ΔE9 mice	2011	Neurosci Lett				x			APPswe/PS1ΔE9		x			x						x				Nicotine, SSR180711
P. Sompol, J. L. Furman, M. M. Pleiss, S. D. Kraner, I. A. Artiushin, S. R. Batten, et al.	Calcineurin/NFAT Signaling in Activated Astrocytes Drives Network Hyperexcitability in Aβ-Bearing Mice	2017	J Neurosci		x		x			5XFAD		x			x	x		x		x					
Y. Song, M. Hu, J. Zhang, Z. Q. Teng and C. Chen	A novel mechanism of synaptic and cognitive impairments mediated via microRNA-30b in Alzheimer's disease	2019	EBioMedicine		x		x	Primary Cells	x	5XFAD		x		NFkB	x			x		x	x				
L. Sosulina, M. Mittag, H. R. Geis, K. Hoffmann, I. Klyubin, Y. Qi, et al.	Hippocampal hyperactivity in a rat model of Alzheimer's disease	2021	J Neurochem	x		x				McGill-R-Thy1-APP		x	x			x	x								
J. P. Spencer, J. T. Brown, J. C. Richardson, A. D. Medhurst, S. S. Sehmi, A. R. Calver and A. D. Randall	Modulation of hippocampal excitability by 5-HT4 receptor agonists persists in a transgenic model of Alzheimer's disease	2004	Neuroscience			x	x			TASTPM		x			x										Prucalopride and SL65.0155, GR113808
M. Srivareerat, T. T. Tran, K. H. Alzoubi and K. A. Alkadhi	Chronic psychosocial stress exacerbates impairment of cognition and long-term potentiation in β-amyloid rat model of Alzheimer's disease	2009	Biol Psychiatry	x								x			x					x	x			Aβ1-40/Aβ1-42	
M. Srivareerat, T. T. Tran, S. Salim, A. M. Aleisa and K. A. Alkadhi	Chronic nicotine restores normal Aβ levels and prevents short-term memory and E-LTP impairment in Aβ rat model of Alzheimer's disease	2011	Neurobiol Aging	x								x			x					x	x			Aβ1-40/Aβ1-42	Nicotine
I. C. Stancu, L. Ris, B. Vasconcelos, C. Marinangeli, L. Goeminne, V. Laporte, et al.	Tauopathy contributes to synaptic and cognitive deficits in a murine model for Alzheimer's disease	2014	Faseb j				x			5xFAD		x			x					x					
V. Steubler, S. Erdinger, M. K. Back, S. Ludewig, D. Fässler, M. Richter, et al.	Loss of all three APP family members during development impairs synaptic function and plasticity, disrupts learning, and causes an autism-like phenotype	2021	Embo j		x		x			NexCre cTKO		x	x		x	x		x		x	x				
E. C. Suh, Y. J. Jung, Y. A. Kim, E. M. Park and K. E. Lee	A β 25-35 induces presynaptic changes in organotypic hippocampal slice cultures	2008	Neurotoxicology					OTC				x						x	x					Aβ25-35	
M. K. Sun and D. L. Alkon	Impairment of hippocampal CA1 heterosynaptic transformation and spatial memory by β-amyloid(25-35)	2002	J Neurophysiology	x		x						x	x			x				x	x			Aβ25-35	
E. M. Szatmari, A. F. Oliveira, E. J. Sumner and R. Yasuda	Centaurin-α1-Ras-Elk-1 signaling at mitochondria mediates β-amyloid-induced synaptic dysfunction	2013	J Neurosci				x	Primary Cells, OTC		APPswe		x		Cent1A-ras-ERK, Elk1		x		x						Aβ1-42	
S. Tabassum, A. Misrani, B. L. Tang, J. Chen, L. Yang and C. Long	Jujuboside A prevents sleep loss-induced disturbance of hippocampal neuronal excitability and memory impairment in young APP/PS1 mice	2019	Sci Rep		x		x			APP/PS1		x	x	CamKII, CREB		x				x					Jujuboside A
R. H. Takahashi, E. Capetillo-Zarate, M. T. Lin, T. A. Milner and G. K. Gouras	Accumulation of intraneuronal β-amyloid 42 peptides is associated with early changes in microtubule-associated protein 2 in neurites and synapses	2013	PLoS One		x					APPswe		x						x	x						
H. Tamano, K. Ide, P. A. Adlard, A. I. Bush and A. Takeda	Involvement of hippocampal excitability in amyloid β-induced behavioral and psychological symptoms of dementia	2016	J Toxicol Sci		x		x					x								x				Aβ1-42	

General Information				Used Model						Genetic Modification		Investigated Neurotransmission		Signalling Pathway	Functional Analysis			Structural Analysis		Behavioral Experiments	Plasticity			Investigated APP fragments and domains	Investigated Substance, Intervention or Condition
Authors	Title	Year	Journal	In Vivo Rat	In Vivo Mouse	Ex Vivo Rat	Ex Vivo Mouse	In Vitro	Human	Transgen Line	APP-Related Viral Modifications	Excitiation	Inhibition		Field Potential Recordings	Whole Cell Recordings	Imaging	Light Microscopy	Electron Microscopy		LTP	LTD	STP		
A. Tamburri, A. Dudilot, S. Licea, C. Bourgeois and J. Boehm	NMDA-receptor activation but not ion flux is required for amyloid-β induced synaptic depression	2013	PLoS One					OTC				x				x								Aβ1-42	
D. Tampellini, N. Rahman, E. F. Gallo, Z. Huang, M. Dumont, E. Capetillo-Zarate, et al.	Synaptic activity reduces intraneuronal Aβ, promotes APP transport to synapses, and protects against Aβ-related synaptic alterations	2009	J Neurosci				x	Primary Cells		Tg19959, Tg2576		x					x	x							
V. T. Y. Tan, B. G. Mockett, S. M. Ohline, K. D. Parfitt, H. E. Wicky, K. Peppercorn, et al.	Lentivirus-mediated expression of human secreted amyloid precursor protein-α prevents development of memory and plasticity deficits in a mouse model of Alzheimer's disease	2018	Mol Brain		x		x	Primary Cells		B6C3-Tg (APPswe, PSEN1ΔE9)85Dbo/ Mmjax	sAPP	x			x					x	x		x	sAPPα	
H. B. C. Taylor, N. J. Emptage and A. F. Jeans	Long-term depression links amyloid-β to the pathological hyperphosphorylation of tau	2021	Cell Rep				x	OTC				x			x	x		x				x		Aβ1-42	
T. T. Tran, M. Srivareerat, I. A. Alhaider and K. A. Alkadhi	Chronic psychosocial stress enhances long-term depression in a subthreshold amyloid-β rat model of Alzheimer's disease	2011	J Neurochem	x								x			x							x		Aβ1-42	Chronic Stress
E. Trillaud-Doppia and J. Boehm	The Amyloid Precursor Protein Intracellular Domain Is an Effector Molecule of Metaplasticity	2018	Biol Psychiatry					OTC		APPExt-APLP2TM-APPICD		x		Ryr		x					x	x		AICD	
M. R. Tropea, D. D. Li Puma, M. Melone, W. Gulisano, O. Arancio, C. Grassi, et al.	Genetic deletion of α7 nicotinic acetylcholine receptors induces an age-dependent Alzheimer's disease-like pathology	2021	Prog Neurobiol		x		x			α7-KO		x			x					x	x				M3.2
E. Varga, G. Juhász, Z. Bozsó, B. Penke, L. Fülöp and V. Szegedi	Aβ(1-42) enhances neuronal excitability in the CA1 via NR2B subunit-containing NMDA receptors	2014	Neural Plast				x					x			x						x			Aβ1-42	
J. Y. Vargas, J. Ahumada, M. S. Arrázola, M. Fuenzalida and N. C. Inestrosa	WASP-1, a canonical Wnt signaling potentiator, rescues hippocampal synaptic impairments induced by Aβ oligomers	2015	Exp Neurol				x	Primary Cells		APPswe/PS1ΔE9		x			x						x				WASP1
A. Volianskis, R. Køstner, M. Mølgaard, S. Hass and M. S. Jensen	Episodic memory deficits are not related to altered glutamatergic synaptic transmission and plasticity in the CA1 hippocampus of the APPswe/PS1δE9-deleted transgenic mice model of β-amyloidosis	2010	Neurobiol Aging		x		x			APPswe/PS1ΔE9		x			x			x		x	x				
K. Wang, W. Sun, L. Zhang, W. Guo, J. Xu, S. Liu, et al.	Oleanolic Acid Ameliorates Aβ25-35 Injection-induced Memory Deficit in Alzheimer's Disease Model Rats by Maintaining Synaptic Plasticity	2018	CNS Neurol Disord Drug Targets	x								x			x				x		x			Aβ25-35	Oleic acid
L. Wang, H. Kang, Y. Li, Y. Shui, R. Yamamoto, T. Sugai and N. Kato	Cognitive recovery by chronic activation of the large-conductance calcium-activated potassium channel in a mouse model of Alzheimer's disease	2015	Neuropharmacology		x		x			3xTg		x			x					x	x				Isopimaric acid
Q. Wang, I. Klyubin, S. Wright, I. Griswold-Prenner, M. J. Rowan and R. Anwyl	α v integrins mediate β-amyloid induced inhibition of long-term potentiation	2008	Neurobiol Aging	x		x						x			x						x			Aβ1-42	
S. Wang, J. Huang, Y. Chen, Y. Liang, L. Chen, D. Ye, et al.	Qifu-yin activates the Keap1/Nrf2/ARE signaling and ameliorates synaptic injury and oxidative stress in APP/PS1 mice	2024	J Ethnopharmacol		x		x			APP/PS1		x						x	x	x					Qifu-yin
X. H. Wang, L. Li, C. Hölscher, Y. F. Pan, X. R. Chen and J. S. Qi	Val8-glucagon-like peptide-1 protects against Aβ1-40-induced impairment of hippocampal late-phase long-term potentiation and spatial learning in rats	2010	Neuroscience	x								x			x					x	x			Aβ1-40	Val8-GLP-1(7–36)
X. L. Wang, Y. X. Deng, Y. M. Gao, Y. T. Dong, F. Wang, Z. Z. Guan, et al.	Activation of α7 nAChR by PNU-282987 improves synaptic and cognitive functions through restoring the expression of synaptic-associated proteins and the CaM-CaMKII-CREB signaling pathway	2020	Aging		x		x	Primary Cells		APP/PS1_DT		x		CaMKII-CREB				x	x	x				Aβ1–42	
Y. Wang, N. H. Greig, Q. S. Yu and M. P. Mattson	Presenilin-1 mutation impairs cholinergic modulation of synaptic plasticity and suppresses NMDA currents in hippocampus slices	2009	Neurobiol Aging				x			3×TgAD		x			x						x				
Y. Wang, L. Wu, J. Li, D. Fang, C. Zhong, J. X. Chen and S. S. Yan	Synergistic exacerbation of mitochondrial and synaptic dysfunction and resultant learning and memory deficit in a mouse model of diabetic Alzheimer's disease	2015	J Alzheimers Dis		x		x			V717F, K670N,M671L		x			x					x	x		x		Diabetes
W. Wei, L. N. Nguyen, H. W. Kessels, H. Hagiwara, S. Sisodia and R. Malinow	Amyloid β from axons and dendrites reduces local spine number and plasticity	2010	Nat Neurosci					OTC			APPtdtomato	x						x							
D. J. Whitcomb, E. L. Hogg, P. Regan, T. Piers, P. Narayan, G. Whitehead, et al.	Intracellular oligomeric amyloid-β rapidly regulates GluA1 subunit of AMPA receptor in the hippocampus	2015	Sci Rep				x	OTC				x		PKA		x								Aβ1-42	
G. M. Wu and X. Y. Hou	Oligomerized Aβ25-35 induces increased tyrosine phosphorylation of NMDA receptor subunit 2A in rat hippocampal CA1 subfield	2010	Brain Res			x						x		src kinase				x						Aβ25-35	Amantadine, PP2
M. N. Wu, Y. X. He, F. Guo and J. S. Qi	α4β2 nicotinic acetylcholine receptors are required for the amyloid β protein-induced suppression of long-term potentiation in rat hippocampal CA1 region in vivo	2008	Brain Res Bull	x								x			x						x		x	Aβ25-35, Aβ31-35, Aβ1-40	Epibatidine, dy-hydro-β-erythroidine
R. Wykes, A. Kalmbach, M. Eliava and J. Waters	Changes in the physiology of CA1 hippocampal pyramidal neurons in preplaque CRND8 mice	2012	Neurobiol Aging				x			CRND8		x				x	x	x							
E. Xia, F. Xu, C. Hu, J. P. P. Kumal, X. Tang, D. Mao, et al.	Young Blood Rescues the Cognition of Alzheimer's Model Mice by Restoring the Hippocampal Cholinergic Circuit	2019	Neuroscience		x		x			APPswe/PSEN1ΔE9		x						x	x	x					Young Blood Serum
W. Yao, H. J. Zou, D. Sun and S. Q. Ren	Aβ induces acute depression of excitatory glutamatergic synaptic transmission through distinct phosphatase-dependent mechanisms in rat CA1 pyramidal neurons	2013	Brain Res			x						x		protein phosphatase 2B, 2A and 1		x								Aβ1-40	Okadaic Acid, FK506
H. Ye, S. Jalini, S. Mylvaganam and P. Carlen	Activation of large-conductance Ca(2+)-activated K(+) channels depresses basal synaptic transmission in the hippocampal CA1 area in APP (swe/ind) TgCRND8 mice	2010	Neurobiol Aging				x			TgCRND8		x			x										BK channel blockers

General Information				Used Model						Genetic Modification		Investigated Neurotransmission		Signalling Pathway	Functional Analysis			Structural Analysis		Behavioral Experiments	Plasticity			Investigated APP fragments and domains	Investigated Substance, Intervention or Condition
Authors	Title	Year	Journal	In Vivo Rat	In Vivo Mouse	Ex Vivo Rat	Ex Vivo Mouse	In Vitro	Human	Transgen Line	APP-Related Viral Modifications	Excitiation	Inhibition		Field Potential Recordings	Whole Cell Recordings	Imaging	Light Microscopy	Electron Microscopy		LTP	LTD	STP		
Q. Yu, Y. Wang, F. Du, S. Yan, G. Hu, N. Origlia, et al.	Overexpression of endophilin A1 exacerbates synaptic alterations in a mouse model of Alzheimer's disease	2018	Nat Commun		x		x	Primary Cells		Sh3gl2, mAPP		x		p38 MAP kinase			x	x		x	x			Aβ1-42	SB203580
D. Zare, M. A. Rajizadeh, M. Maneshian, H. Jonaidi, V. Sheibani, M. Asadi-Shekaari, et al.	Inhibition of protease-activated receptor 1 (PAR1) ameliorates cognitive performance and synaptic plasticity impairments in animal model of Alzheimer's diseases	2021	Psychopharmacology	x								x			x					x	x		x	Aβ1–42	SCH
D. Zhang, A. J. Mably, D. M. Walsh and M. J. Rowan	Peripheral Interventions Enhancing Brain Glutamate Homeostasis Relieve Amyloid β- and TNFα- Mediated Synaptic Plasticity Disruption in the Rat Hippocampus	2017	Cereb Cortex	x								x			x						x			Aβ1–42	Oxaloacetate
J. F. Zhang, J. S. Qi and J. T. Qiao	Protein kinase C mediates amyloid β-protein fragment 31-35-induced suppression of hippocampal late-phase long-term potentiation in vivo	2009	Neurobiol Learn Mem	x								x		PKC	x						x			Aβ31–35, Aβ25–35	Chelerythrine, PMA
W. Zhang, J. Miao, J. Hao, Z. Li, J. Xu, R. Liu, et al.	Protective effect of S14G-humanin against β-amyloid induced LTP inhibition in mouse hippocampal slices	2009	Peptides				x					x		CREB	x						x			Aβ25–35	S14G-humanin
X. Zhang, U. Herrmann, S. W. Weyer, M. Both, U. C. Müller, M. Korte and A. Draguhn	Hippocampal network oscillations in APP/APLP2-deficient mice	2013	PLoS One				x			APP-KO, APPsα-KI, APPsa/sa/APLP2KO /KO		x			x								x		
Z. Zhang, R. Chen, W. An, C. Wang, G. Liao, X. Dong, et al.	A novel acetylcholinesterase inhibitor and calcium channel blocker SCR-1693 improves Aβ25-35-impaired mouse cognitive function	2016	Psychopharmacology		x		x	Primary Cells				x		CaMKII, ERK, CREB, and Akt	x					x	x			Aβ25–35	SCR-1693
W. H. Zhi, Y. Y. Zeng, Z. H. Lu, W. J. Qu, W. X. Chen, L. Chen and L. Chen	Simvastatin exerts antiamnesic effect in Aβ25-35 -injected mice	2014	CNS Neurosci Ther		x		x					x		Akt, ERK	x					x	x			Aβ25–35	Simvastatin
R. Zhou and P. Bickler	Interaction of Isoflurane, Tumor Necrosis Factor-α and β-Amyloid on Long-term Potentiation in Rat Hippocampal Slices	2017	Anesth Analg			x						x			x						x		x	Aβ1-42	Isofluran
