# Supplementary material for: A synapse perspective on the function of the amyloid precursor protein
Source: Sci Prog. 2025 Jul 30;108(3):00368504251360728. doi: 10.1177/00368504251360728 (PMC12317227; doi:10.1177/00368504251360728)
Supplement: sj-pdf-3-sci-10.1177_00368504251360728 - Supplemental material for A synapse perspective on the function of the amyloid precursor protein [file sj-pdf-3-sci-10.1177_00368504251360728.pdf]

The literature search was conducted using the database PubMed on September 29, 2024. As search terms in PubMed advanced search builder the following words were used: amyloid precursor protein AND synaptic transmission AND hippocampus NOT review NOT systematic review NOT meta-analysis. Based on inclusion and exclusion criteria as well as full text availability, 309 papers remained for further analysis and these papers were screened for their key features. These features are depicted in the tables of this document. Since the CA1 region is the focus of this review, the papers conducting experiments in this region, are depicted in more detail.

Inclusion Criteria	Exclusion Criteria
Original research article<br>Peer-reviewed<br>Full text availability<br>English<br>Analysis of functional or structural synaptic features	Brain regions other than hippocampus<br>Computational-only work<br>No baseline experiments
